# Supplementary material for: Ethical Dilemmas at the Beginning and End of Life: A Needs-Based, Experience-Informed, Small-Group, Case-Based Curriculum for Pediatric Residents
Source: MedEdPORTAL. 2020 Apr 3;16:10895. doi: 10.15766/mep_2374-8265.10895 (PMC7187913; doi:10.15766/mep_2374-8265.10895)
Supplement: Supplementary file 1 — Medically Provided Fluids Nutrition PowerPoint.pptxMedically Provided Fluids Nutrition Instructor Guide.docxMedically Provided Fluids Nutrition Handout.docxMedically Provided Fluids Nutrition Assessment Questions.docxFutility and Goals of Care PowerPoint.pptxFutility and Goals of Care Instructor Guide.docxFutility and Goals of Care Handout.docxFutility and Goals of Care Assessment Questions.docxEthical Issues in Neonatology PowerPoint.pptxEthical Issues in Neonatology Instructor Guide.docxEthical Issues in Neonatology Assessment Questions.docx [file mep-16-10895-s001.zip › _Educational_Summary_Report_10895.pdf]

MedEdPORTAL® The AAMC Journal of Teaching and Learning Resources

Original Publication Open Access

Ethical Dilemmas at the Beginning and End of Life: A Needs-Based, Experience-Informed, Small-Group, Case-Based Curriculum for Pediatric Residents

Lori A. Herbst, MD*, Jennifer deSante-Bertkau, MD, MBE

*Corresponding Author: lori.herbst@cchmc.org

Abstract

Introduction: Pediatric residents are faced with ethical dilemmas in beginning- and end-of-life situations throughout their training. These situations are innately challenging, yet despite recommendations that residents receive training in ethics and end-of-life domains, they continue to report the need for additional training. To address these concerns, we developed an interactive and reflective palliative care and medical ethics curriculum including sessions focusing on ethical dilemmas at the beginning and end of life. **Methods:** This module includes a trio of case-based, small-group discussions on artificial nutrition and hydration, futility, and ethical considerations in neonatology. Content was developed based on a needs assessment, input from local experts, and previously published material. Trainees completed assessments of comfort and understanding before and after each session. **Results:** The module was attended and assessed by an average of 27 trainees per session, including residents and medical students. Knowledge of ethical considerations improved after individual sessions, with 86% of trainees reporting understanding ethical considerations involved in the decision to withdraw or withhold medically provided nutrition and hydration and 67% of trainees reporting understanding the use of the term *futility*. Trainee comfort in providing counseling or recommendations regarding specific ethical issues demonstrated a trend toward improvement but did not reach statistical significance. **Discussion:** We successfully implemented this innovative module, which increased trainees' comfort with end-of-life care and ethical conflicts. Future studies should focus on the trainees' ability to implement these skills in clinical practice.

Keywords
Pediatrics, Medical Ethics, Ethics/Bioethics, Palliative Care, Terminal Care, End of Life, Futility, Neonatology, Artificial Nutrition, Hospice, Palliative Medicine, Neonatal-Perinatal Medicine

Educational Objectives

By the completion of this module, learners will be able to:

1. Describe ethical challenges encountered in beginning- and end-of-life care.
2. Report increased comfort navigating ethical conflicts and dilemmas.
3. Construct an approach to ethical conflicts and dilemmas including utilization of outside resources when needed.

Citation:
Herbst LA, deSante-Bertkau J. Ethical dilemmas at the beginning and end of life: a needs-based, experience-informed, small-group, case-based curriculum for pediatric residents. *MedEdPORTAL*. 2020;16:10895. https://doi.org/10.15766/mep_2374-8265.10895

Introduction

Some of the most challenging aspects of medicine are the grey areas, particularly those involving ethical dilemmas at the beginning and end of life. While medical ethics and palliative medicine are two specialties available to provide support to both families and providers at many institutions, residents are often the frontline providers for the care of these patients in the hospital. As such, they frequently have to navigate challenging internal ethical dilemmas (not knowing what to do) and the resulting interpersonal conflict (when people disagree about what to do) that can develop in these scenarios. In many instances, residents face these ethical challenges at the extremes of life without explicit training on ways to approach such encounters.[1-5]

Since 1987, ethical decision making has been part of the American Board of Pediatrics (ABP) Certifying Exam.[6] In 1997, the Accreditation Counsel for Graduate Medical Education (ACGME) began requiring a structured curriculum in medical ethics

Copyright © 2020 Herbst and deSante-Bertkau. This is an open-access publication distributed under the terms of the Creative Commons Attribution license.

for all pediatric residency programs.[7] Physicians face ethical challenges, and the ACGME recognized that trainees should demonstrate adherence to ethical principles as a component of professionalism.[8] Despite these requirements, surveys of pediatric residency program directors revealed significant variability in how ethics and professionalism were taught in pediatric residency programs.[7,9] Some of the most recent data come from a 2009 survey of pediatric program directors in which 80% of respondents reported using lectures to teach ethics and 72% used seminars based on real cases. Despite this, only 29% of respondents reported being knowledgeable about the topics covered in their ethics curriculum.[7] In 2011, the American Academy of Pediatrics (AAP) published a bioethics teaching guide for pediatric residents.[10] However, based on a 2013 survey of case-based pediatric program directors, many respondents were unaware of resources to teach ethics and professionalism.[9] Given the significant variation in how and what pediatric residents are taught regarding medical ethics, it is not surprising that a majority of recent pediatric residency graduates reported gaps in their ethics education and knowledge.[5,11,12] Residents similarly have reported minimal training regarding discussions about death and end-of-life care,[4,13] which makes addressing ethical dilemmas at the end of life even more challenging.

Although information on residents' perceptions of ethical dilemmas at the beginning and end of life is not extensive, what data are available indicate that these issues rank highly among scenarios where residents request more education and guidance. A 2005 survey asked internal medicine residents to rank the situations for which they would most likely request an ethics consultation. "Medical Futility" and "Artificial Nutrition & Hydration" were two of the highest-ranking situations, particularly among senior residents.[14] Another survey of medical students and residents asked respondents to assess their level of comfort in dealing with clinical ethics issues. Less than 60% of respondents stated that they were comfortable with issues regarding forgoing life-sustaining treatments and medical futility.[5] Additionally, while data specific to residents are sparse, neonatal intensive care unit (NICU) fellows have reported gaps in their ethics training, with one study demonstrating that only 37% of fellows felt their ethics education was very good/excellent and that 30% felt they had received no ethics education.[15] Practicing pediatricians also have reported lack of confidence addressing these complex ethical challenges, with only 19% of pediatricians feeling confident addressing withdrawal of artificial nutrition and hydration and 32% feeling confident discussing attempting resuscitation for a preterm infant on the margin of viability.[3] Additionally, only 29% of pediatricians felt confident addressing life-sustaining therapies for infants with severe neurocognitive disabilities, 30% of pediatricians felt confident withdrawing mechanical ventilation, and 51% of pediatricians felt confident having do-not-resuscitate discussions, all of which are topics commonly encountered in the NICU or when addressing futility.[3]

To bridge this gap, we have developed, implemented, and evaluated an interactive, case-based, longitudinal palliative care and medical ethics curriculum that includes targeted modules on ethical dilemmas at the beginning and end of life. Previously published curricula focusing on ethical dilemmas at the beginning and end of life in pediatrics are limited. We were unable to identify any pediatric curricula specifically on withholding or withdrawing medically provided nutrition and hydration; however, prior published curricula have included this topic within broader discussions of end-of-life care.[16,17] Curricula focused on ethical challenges in the NICU have utilized standardized patient encounters, an approach that was not feasible to implement within our general residency curriculum.[18,19] The AAP Section on Bioethics offers a curriculum that includes a session entitled Brain Death, Permanent Vegetative State, and Medical Futility.[10] Recently, a comprehensive pediatric palliative care curriculum utilizing online modules and in-person conferences and including a section on medical ethics was published.[20] Our curriculum is unique in the content it addresses and in that it offers targeted sessions that can be easily incorporated within a traditional residency curriculum for pediatric residents and medical students rotating on pediatric clerkships.

Methods

Curriculum Design and Implementation

We developed our longitudinal curriculum using Kern's six-step approach.[21] Our residents completed a targeted needs assessment initially focused on end-of-life educational needs, highlighting the need for additional education focused on end-of-life care and the ethical issues that arise in these scenarios.[22]

We utilized results from the needs assessment, the AAP bioethics residency curriculum,[10] personal experience and training, and input from local experts in palliative care and medical ethics to develop content. The module was divided into three individual sessions and fit within a broader 18-month curriculum that aligned with the structure of the general pediatric resident curriculum. This allowed sessions to repeat during the course of residency to increase the opportunity among residents for exposure to all sessions.

Copyright © 2020 Herbst and deSante-Bertkau. This is an open-access publication distributed under the terms of the Creative Commons Attribution license.

We obtained support from the pediatric residency program and chief residents to implement this curriculum during morning report sessions given the ACGME requirements for education on medical ethics and gaps in the current curriculum. We chose to utilize a small-group discussion format to incorporate adult learning strategies based on feedback from the residency program and chief residents. Once we had garnered support from the pediatric residency program leadership, the chief residents proved integral to the ongoing success of the program and to scheduling the sessions on an annual basis.

Subjects and Session Structure

The Ethical Dilemmas at the Beginning and End of Life module included three sessions: Medically Provided Fluids and Nutrition (Appendices A-D), Futility and Goals of Care (Appendices E-H), and Ethical Issues in Neonatology (Appendices I-K). We targeted all learners rotating on inpatient pediatric services, including interns, residents (postgraduate years 2-5), and third- and fourth-year medical students. Learners were divided into small groups based on their current clinical rotation, which ensured all groups included interns, senior residents, and medical students (when present). Small-group discussion utilized a problem-based learning format that had been successfully employed in other case discussions in the pediatric residency curriculum. Attending physicians with board certification in palliative care or a master's degree in medical ethics led PowerPoint lectures to provide content and guide small-group discussions led by the senior residents. All guidance regarding formal palliative care and ethical frameworks was included in the PowerPoints (Appendices A, E, & I) and instructor directions (Appendices B, F, & J). Hospital medicine (HM) attending physicians on service at the time of the session were present to serve as facilitators based on their experiences as attendings facing these challenges. Two or three times during each session, we posed questions to the audience, at times including additional handouts (Appendices C & G), for small-group discussion. Residents had access to a flip chart to document key points from their discussion. At the end of the small-group discussions, individual groups were asked to report highlights to the entire audience and obtain feedback from the presenters.

Assessment Tools

We developed assessment tools to evaluate learner understanding and comfort for each individual session within the module as there was a lack of standard assessment tools in medical ethics education. The evaluations for each session were developed by the session leader based on the session content and specific learning objectives. The evaluations asked learners to indicate their level of training and contained a mixture of 5-point Likert-scale questions, multiple-choice questions, and/or true-false questions. Content experts piloted the evaluations prior to their use by the residents. Learners were asked to complete both pre- and postsession evaluations for each session (Appendices D, H, & K), and a majority of learners did so.

Institutional review board approval for this educational intervention was obtained. Participation in the educational session was required for all residents as part of the standard morning report curriculum, but completion of the evaluations was voluntary. Written informed consent was waived by the institutional review board, and consent was implied by completion of the evaluations. All evaluations were anonymous.

Data Analysis

Responses from the pre- and postsession Likert-scale questions evaluating learning objectives for the morning report case-based small-group discussions were dichotomized as learners who agreed that the session met an objective (agree and strongly agree) and others (strongly disagree, disagree, and neutral). They were analyzed using chi-square analysis. Subgroup analysis based on the level of learner training was also performed. We were unable to analyze multiple-choice and true-false questions due to discrepancies in what content the questions measured; however, we utilized these questions to improve future lectures in this series.

Results

Although the number and type of learner (i.e., level of training) varied with each session, the average number of learners who attended each of the three Ethical Dilemmas at the Beginning and End of Life sessions and completed the evaluations was 27. Incomplete evaluation forms were excluded from data analysis. The average number of residents who attended each session was 19, of whom 13 were interns. Medical students accounted for an average of 11 learners per session attended and participated in the Futility and Goals of Care session and the Medically Provided Fluids and Nutrition session. We chose to analyze subgroups of medical students, all residents, and interns to better understand which learners gained the most benefit from these sessions. Learners who did not report their level of training were analyzed with the all-learners group only.

The number of learners expressing comfort with or understanding of learning objectives increased for most objectives across sessions and across all learner subgroups. More than two-thirds of learners reported understanding of ethical considerations

Copyright © 2020 Herbst and deSante-Bertkau. This is an open-access publication distributed under the terms of the Creative Commons Attribution license.

addressed in the sessions, but only a third to a half of learners felt comfortable making treatment recommendations or counseling patients regarding ethical issues. Medical students reported similar levels of comfort aside from comfort counseling families on decisions to withdraw or withhold medically provided nutrition/hydration. Only one student reported feeling comfortable counseling on this topic both before and after the session (Table).

Discussion

We successfully created an innovative module focused on ethical dilemmas at the beginning and end of life as part of a broader palliative care and medical ethics curriculum, thus fulfilling an identified need among our pediatric residents and also meeting the educational needs of medical students.

Residents at our institution[22] confirmed previously identified gaps in medical ethics education.[3,5,7,11,12,14,15] This module is a valuable addition to formal medical ethics education, which is recognized as a requirement by the ACGME, ABP, and AAP.[7,8] It adds to previously published curricula utilizing problem-based learning to address futility in the adult setting[23] by providing content specific to pediatrics. Additionally, it utilizes case-based learning to explore ethical considerations in neonatology, which may be more accessible for some learning environments than previously published curricula utilizing standardized patients.[18,19] Finally, it adds a novel component by featuring a focused session on the ethical challenges surrounding medically provided nutrition and hydration at the end of life.

Improvement in self-assessment scores after the completion of each individual session occurred to varying degrees. At baseline, less than 50% of learners, and in most cases less than 25%, felt comfortable with the skill or knowledge assessed, which highlights the need for ethics training surrounding the beginning and end of life. While not all self-assessment questions reached statistical significance, there was a trend toward improved understanding or comfort for all areas assessed. Not only did topics focused on understanding general ethical considerations improve, but the percentage of learners reporting comfort or understanding was between 64% and 86% following the sessions, confirming that these modules were able to provide a baseline knowledge of ethical considerations to a majority of the learners. In contrast, while a greater number of learners felt comfortable counseling or making recommendations regarding ethical issues following the sessions, the percentage of all learners feeling comfortable remained between 36% and 57% following the sessions. This suggests an additional need for hands-on practice with patients that small-group discussions could not simulate. This pattern was highlighted by the Medically Provided Fluids and Nutrition session, where residents reported understanding the risks and benefits of medically provided nutrition and hydration at the end of life and the ethical considerations surrounding withdrawal or

Table. Improvement in Learner Comfort on Session Objectives

	All Residents			Interns			Medical Students			All Learners		
	% Agree			% Agree			% Agree			% Agree		
Session and Question	Pre	Post	*p*	Pre	Post	*p*	Pre	Post	*p*	Pre	Post	*p*
Medically Provided Fluids and Nutrition[a]												
I feel comfortable describing the benefits and burdens of medically provided nutrition/hydration at the end of life.	24.0	77.9	<.01	11.8	64.7	.02	18.2	45.4	.17	22.2	66.7	<.01
I feel comfortable counseling families on the decision to withdraw or withhold medically provided nutrition/hydration at the end of life.	24.0	48.0	.08	11.8	35.3	.10	9.1	9.1	1.00	19.4	36.1	.11
I understand the ethical considerations involved in the decision to withdraw or withhold medically provided hydration/nutrition.	48.0	88.0	<.01	47.1	82.4	.03	36.4	81.8	.03	44.4	86.1	<.01
I understand the distinction between medical intervention and basic patient care.	44.0	68.0	.09	47.1	64.7	.30	45.5	54.6	.67	44.4	63.9	.10
Futility and Goals of Care[b]												
I am comfortable that I understand how the term futility is used in a clinical context.	17.7	58.8	.01	25.0	75.0	.01	0.0	81.8	<.01	10.0	66.7	<.01
I am comfortable recommending a treatment plan based on a patient or family's goals of care.	23.5	58.8	.04	33.3	75.0	.04	9.1	45.5	.06	20.0	53.3	<.01
Ethical Issues in Neonatology[c]												
I understand when it is appropriate not to offer or withhold certain medical interventions.	21.4	57.1	.05	11.1	55.6	.05						
I understand what information goes into a prognostic assessment in the periviable period.	14.3	85.7	<.01	0.0	77.8	<.01						

[a]All residents: $N = 25$, interns: $N = 17$, medical students: $N = 11$, all learners: $N = 36$.
[b]All residents: $N = 17$, interns: $N = 12$, medical students: $N = 11$, all learners: $N = 30$.
[c]All residents: $N = 14$, interns: $N = 9$, medical students: $N = 0$.

Copyright © 2020 Herbst and deSante-Bertkau. This is an open-access publication distributed under the terms of the Creative Commons Attribution license.

withholding nutrition but lacked comfort counseling families on these decisions.

While this module was developed primarily to address a gap in resident education, our experience demonstrates that this gap exists for medical students as well. Although we could not compare improvement across subgroups due to numbers, we noticed similar trends in improvement for sessions where medical students participated, except for the question focused on counseling families on the decision to withdraw or withhold medically provided nutrition/hydration. We feel that the lack of improvement for this objective was likely due to the compounded effect of a lack of clinical experience and a complicated ethical topic. The overall improvement in medical student comfort and knowledge also indicates that our curriculum could be successfully implemented with other learners outside of pediatric residencies.

Implementation Lessons Learned

Overall, the Ethical Dilemmas at the Beginning and End of Life module as part of a broader palliative care and medical ethics curriculum was well received by the residency program leadership as well as individual residents and medical students. One challenge we faced following initial curriculum development and implementation was the breadth of content that could be included when educating on palliative care and medical ethics.[22] We therefore chose to develop a focused morning report curriculum from the most high-yield topics based on input from local content experts and a resident needs assessment. This allowed us to capture the largest number of residents at each individual session compared to embedding sessions within specific rotations or electives that may have had only a few learners at any given time. Additionally, we felt that any overlap based on rotation-specific curricula was likely useful for reinforcing the concepts given the generally uncommon occurrence of these ethical dilemmas.

In addition to the improvement in understanding and comfort reported on evaluations, we felt that the amount of engagement spent during discussions was indicative of learners' interest in the topic. We found that if our sessions had not been constrained by the start of morning rounds, we could have spent longer in discussion. One pitfall regarding case-based discussion that we quickly learned to avoid was allowing learners to pick their own groups as this led students, interns, and seniors to often be segregated. We found that upper-level residents were important in each group as they had the most experience and could serve as facilitators. We subsequently chose to assign tables based on current clinical rotation so that all tables included all levels of learners.

The lecturer for each session had advanced training in either medical ethics or palliative care. While this was an added benefit, we believe that an attending with clinical experience and interest in the field could serve as a facilitator. As part of our sessions, our HM attendings helped guide conversation since they often had participated in similar cases even if they were not content experts. The use of general HM attendings or experienced senior residents was critical to the success of the small-group discussions given that we did not have enough experts in palliative care and ethics to lead all the discussions.

Limitations and Future Directions

The results were limited by small sample size. While our residency program has over 200 combined and categorical pediatric residents and between 12 and 20 medical students rotating on inpatient wards per month, conference attendance at each session was limited by learners' clinical schedules and competing responsibilities. Specifically, trainees on outpatient rotations frequently were in clinic during this time. Additionally, residents could have had a heavier inpatient load in the winter and no medical students rotating in December, which was likely why Ethical Issues in Neonatology had particularly low attendance. To address attendance concerns, the pediatric residency program repeats the resident curriculum, including this module, every 18 months so that each resident has at least two opportunities to attend every session during training. To improve attendance, future work should focus on learner attendance not just during these sessions but for morning report in general.

Generalizability of this module to other institutions may be limited based on the structure of the resident curriculum, the availability of content experts, the legal environment, and the experience of the individual institution. To minimize the impact of structural differences, the individual sessions can be completed in 30-60 minutes based on time allotted for discussion. Additionally, if the education structure utilizes half-day sessions, the entire module can be completed at one time. A half-day session would provide the additional benefit of taking the learners out of their clinical rotations and, therefore, minimizing the number unable to attend due to clinical obligations. The need for multiple content experts would also be minimized as sessions are designed for residents to lead small-group discussions and then solicit feedback form the group and content experts present. Finally, because the cases were developed based on experiences faced at our

Copyright © 2020 Herbst and deSante-Bertkau. This is an open-access publication distributed under the terms of the Creative Commons Attribution license.

institution, some case discussions might need to be adjusted based on the presence or absence of futility policies or state laws regarding the withdrawal of medically provided nutrition and hydration. However, the critical ethics concepts can be addressed through other case discussions that incorporate local policies and laws.

There were several limitations regarding the assessment tools utilized. First, the assessment tools were not validated as this was a novel curriculum and questions were developed from session learning objectives. Second, we assessed the learners only immediately before or after the session. This would not account for knowledge decay, which may occur given the infrequency that learners experience these ethical dilemmas. Third, we were unable to assess knowledge or demonstrate how residents and medical students applied these skills to their medical practice. Finally, we were unable to elicit significant qualitative feedback from open-ended assessment questions. However, anecdotal feedback suggests that the residents especially valued the small-group discussions led by HM attendings. The overall amount of discussion and our frequent need to cut off discussions due to time constraints also demonstrated the residents' engagement in the curriculum.

While the results suggest that our curriculum was successful in achieving our learning objectives for many learners, future work should focus on the development of curricula that allow residents to demonstrate their ability to process ethical challenges and provide relevant recommendations and information to patients and families. Simulation or role-play would allow learners to operationalize the knowledge they acquire in the small-group sessions. Assessment tools should be developed that assess learner knowledge acquisition and can be applied to standardized patients or real-life situations. Additionally, longitudinal experiences should be considered to explore retention of knowledge over time.

Conclusions

As part of a comprehensive longitudinal curriculum focused on pediatric palliative care and medical ethics, this module on ethical dilemmas at the beginning and end of life improved self-reported comfort and attitudes for residents and medical students rotating on pediatrics. The module helps fulfill the recommendations of the ACGME, the ABP, and the AAP that residents should receive education on palliative care and medical ethics during residency. Further studies should evaluate resident knowledge of these topics as well as how residents utilize this knowledge in their clinical practice.

Appendices

A. Medically Provided Fluids Nutrition PowerPoint.pptx
B. Medically Provided Fluids Nutrition Instructor Guide.docx
C. Medically Provided Fluids Nutrition Handout.docx
D. Medically Provided Fluids Nutrition Assessment Questions.docx
E. Futility and Goals of Care PowerPoint.pptx
F. Futility and Goals of Care Instructor Guide.docx
G. Futility and Goals of Care Handout.docx
H. Futility and Goals of Care Assessment Questions.docx
I. Ethical Issues in Neonatology PowerPoint.pptx
J. Ethical Issues in Neonatology Instructor Guide.docx
K. Ethical Issues in Neonatology Assessment Questions.docx

All appendices are peer reviewed as integral parts of the Original Publication.

Lori A. Herbst, MD: Assistant Professor, Department of Pediatrics, Division of Hospital Medicine, Cincinnati Children's Hospital Medical Center; Assistant Professor, Department of Pediatrics, Division of Hospital Medicine, University of Cincinnati College of Medicine; Volunteer Assistant Professor, Department of General Internal Medicine, University of Cincinnati College of Medicine

Jennifer deSante-Bertkau, MD, MBE: Assistant Professor, Department of Pediatrics, Division of Hospital Medicine, Cincinnati Children's Hospital Medical Center; Assistant Professor, Department of Pediatrics, Division of Hospital Medicine, University of Cincinnati College of Medicine; Assistant Professor, Department of Pediatrics, Ethics Center, Cincinnati Children's Hospital Medical Center; Assistant Professor, Department of Pediatrics, Ethics Center, University of Cincinnati College of Medicine

Acknowledgments
Thanks to Dr. Armand Antommaria, Jan Borgman, and Marc Zumhagen for assistance with the content development and acting as facilitators for sessions. Thanks to Dr. Jennifer O'Toole for her guidance regarding development of the assessment tools and Dr. Lilliam Ambroggio for her guidance on statistical analysis.

Disclosures
None to report.

Funding/Support
None to report.

Ethical Approval
The Cincinnati Children's Hospital Medical Center Institutional Review Board approved this study.

Copyright © 2020 Herbst and deSante-Bertkau. This is an open-access publication distributed under the terms of the Creative Commons Attribution license.

References

1. Baker JN, Torkildson C, Baillargeon JG, Olney CA, Kane JR. National survey of pediatric residency program directors and residents regarding education in palliative medicine and end-of-life care. *J Palliat Med*. 2007;10(2):420-429. https://doi.org/10.1089/jpm.2006.0135
2. Hollingsworth CE, Wesley C, Huckridge J, Finn GM, Griksaitis MJ. Impact of child death on paediatric trainees. *Arch Dis Child*. 2018;103(1):14-18. https://doi.org/10.1136/archdischild-2017-313544
3. Kesselheim JC, Johnson J, Joffe S. Pediatricians' reports of their education in ethics. *Arch Pediatr Adolesc Med*. 2008;162(4): 368-373. https://doi.org/10.1001/archpedi.162.4.368
4. McCabe ME, Hunt EA, Serwint JR. Pediatric residents' clinical and educational experiences with end-of-life care. *Pediatrics*. 2008; 121(4):e731-e737. https://doi.org/10.1542/peds.2007-1657
5. Silverman HJ, Dagenais J, Gordon-Lipkin E, et al. Perceived comfort level of medical students and residents in handling clinical ethics issues. *J Med Ethics*. 2013;39(1):55-58. https://doi.org/10.1136/medethics-2011-100300
6. American Board of Pediatrics, Medical Ethics Subcommittee. Teaching and evaluation of interpersonal skills and ethical decision making in pediatrics. *Pediatrics*. 1987;79(5):829-833.
7. Lang CW, Smith PJ, Ross LF. Ethics and professionalism in the pediatric curriculum: a survey of pediatric program directors. *Pediatrics*. 2009;124(4):1143-1151. https://doi.org/10.1542/peds.2009-0658
8. *ACGME Program Requirements for Graduate Medical Education in Pediatrics*. Accreditation Council for Graduate Medical Education website. https://www.acgme.org/Portals/0/PFAssets/ProgramRequirements/320_Pediatrics_2019.pdf?ver=2019-06-18-155134-967. Published July 1, 2019.
9. Cook AF, Sobotka SA, Ross LF. Teaching and assessment of ethics and professionalism: a survey of pediatric program directors. *Acad Pediatr*. 2013;13(6):570-576. https://doi.org/10.1016/j.acap.2013.07.009
10. Bioethics case-based teaching guides for resident training. American Academy of Pediatrics website. https://www.aap.org/en-us/continuing-medical-education/Bioethics-Cased-Based-Teaching-Guides/Pages/Bioethics-Case-Based-Teaching-Guides.aspx
11. Cook AF, Ross LF. Young physicians' recall about pediatric training in ethics and professionalism and its practical utility. *J Pediatr*. 2013;163(4):1196-1201. https://doi.org/10.1016/j.jpeds.2013.04.006
12. Kesselheim JC, Najita J, Morley D, Bair E, Joffe S. Ethics knowledge of recent paediatric residency graduates: the role of residency ethics curricula. *J Med Ethics*. 2016;42(12):809-814. https://doi.org/10.1136/medethics-2016-103625
13. Michelson KN, Ryan AD, Jovanovic B, Frader J. Pediatric residents' and fellows' perspectives on palliative care education. *J Palliat Med*. 2009;12(5):451-457. https://doi.org/10.1089/jpm.2008.0263
14. Fernandes R, Hashimoto S, Masaki K. Enhancing residents' training in medical ethics: an exploratory study assessing attitudes of internal medicine residents. *Hawaii Med J*. 2008;67(12):317-321.
15. Cummings CL, Geis GM, Kesselheim JC, Sayeed S. Ethics and professionalism education during neonatal–perinatal fellowship training in the United States. *J Perinatol*. 2015;35(10):875-879. https://doi.org/10.1038/jp.2015.70
16. Keefer P, Pituch K, Murphy T, et al. A child's last hours—multidisciplinary training in end-of-life care for professionals working in children's hospitals: newborn with lethal congenital anomalies. *MedEdPORTAL*. 2015;11:10108. https://doi.org/10.15766/mep_2374-8265.10108
17. Marks A, Hollenkamp E, Bradman S, et al. A child's last hours—multidisciplinary training in end-of-life care in children's hospitals: school-aged child with terminal cancer. *MedEdPORTAL*. 2016;12:10371. https://doi.org/10.15766/mep_2374-8265.10371
18. Daboval T, Ferretti E, Rohde K, Muirhead P, Moore G. Neonatal ethics teaching program—scenario-oriented learning in ethics: antenatal consultation at the limit of viability. *MedEdPORTAL*. 2015;11:10043. https://doi.org/10.15766/mep_2374-8265.10043
19. Moore G, Ferretti E, Rohde K, Muirhead P, Daboval T. Neonatal ethics teaching program—scenario-oriented learning in ethics: critically ill newborn in the neonatal intensive care unit. *MedEdPORTAL*. 2015;11:10083. https://doi.org/10.15766/mep_2374-8265.10083
20. Friedrichsdorf SJ, Remke S, Hauser J, et al. Development of a pediatric palliative care curriculum and dissemination model: Education in Palliative and End-of-Life Care (EPEC) Pediatrics. *J Pain Symptom Manage*. 2019;58(4):707-720.e3. https://doi.org/10.1016/j.jpainsymman.2019.06.008
21. Kern DE, Thomas PA, Hughes MT, eds. *Curriculum Development for Medical Education: A Six-Step Approach*. 2nd ed. Baltimore, MD: Johns Hopkins University Press; 2009.
22. Wilson PM, Herbst LA, Gonzalez-del-Rey J. Development and implementation of an end-of-life curriculum for pediatric residents. *Am J Hosp Palliat Care*. 2018;35(11):1439-1445. https://doi.org/10.1177/1049909118786870
23. Gaffney MM, Cottingham AH. Four medical ethics team-based learning modules. *MedEdPORTAL*. 2012;8:9268. https://doi.org/10.15766/mep_2374-8265.9268

Received: April 26, 2019
Accepted: November 5, 2019
Published: April 3, 2020

Copyright © 2020 Herbst and deSante-Bertkau. This is an open-access publication distributed under the terms of the Creative Commons Attribution license.
