# Supplementary material for: Ethical Dilemmas at the Beginning and End of Life: A Needs-Based, Experience-Informed, Small-Group, Case-Based Curriculum for Pediatric Residents
Source: MedEdPORTAL. 2020 Apr 3;16:10895. doi: 10.15766/mep_2374-8265.10895 (PMC7187913; doi:10.15766/mep_2374-8265.10895)
Supplement: Supplementary file 1 — Medically Provided Fluids Nutrition PowerPoint.pptxMedically Provided Fluids Nutrition Instructor Guide.docxMedically Provided Fluids Nutrition Handout.docxMedically Provided Fluids Nutrition Assessment Questions.docxFutility and Goals of Care PowerPoint.pptxFutility and Goals of Care Instructor Guide.docxFutility and Goals of Care Handout.docxFutility and Goals of Care Assessment Questions.docxEthical Issues in Neonatology PowerPoint.pptxEthical Issues in Neonatology Instructor Guide.docxEthical Issues in Neonatology Assessment Questions.docx [file mep-16-10895-s001.zip › A. Medically Provided Fluids Nutrition PowerPoint.pptx]

## Slide 1
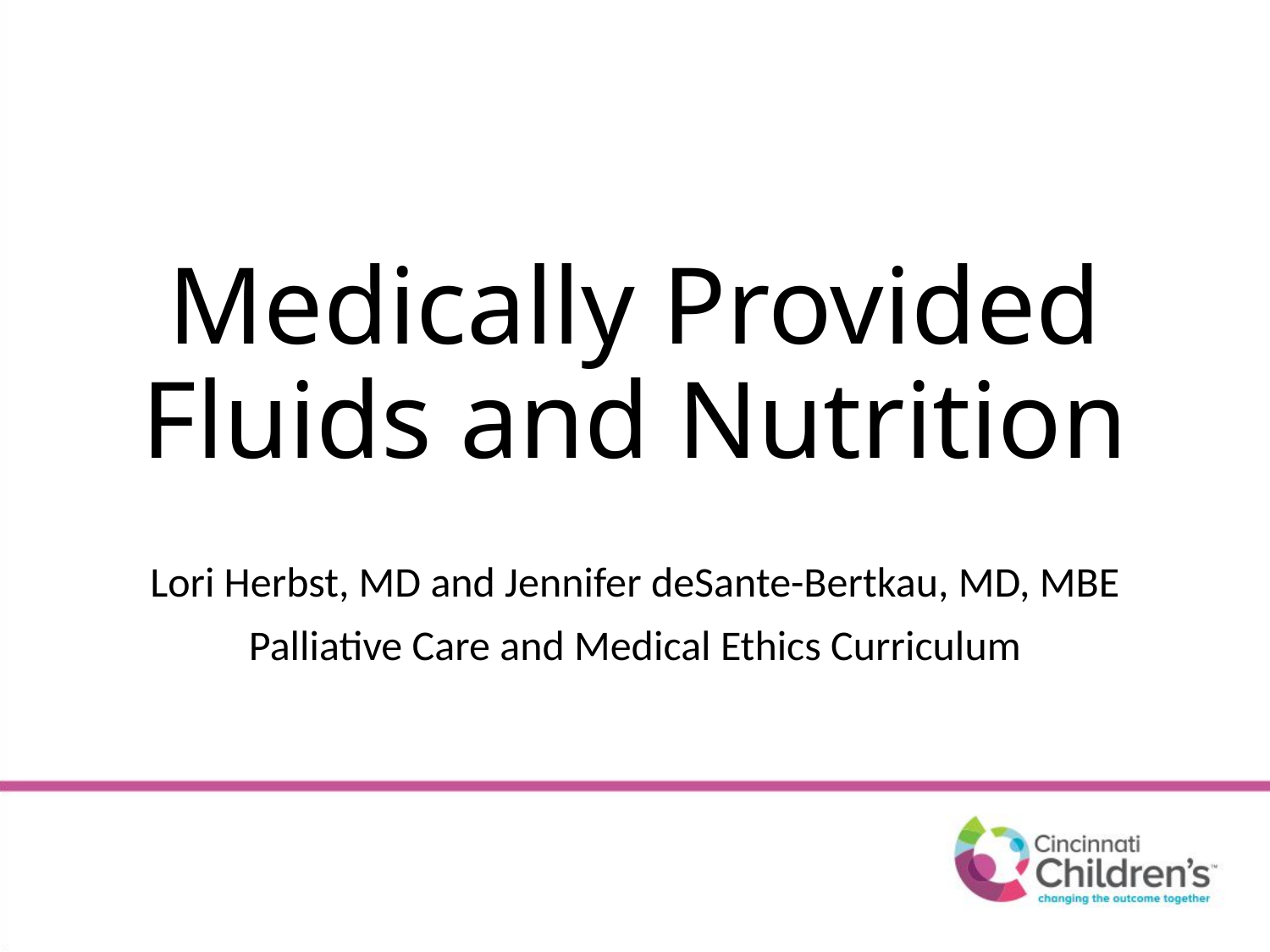

# Medically Provided Fluids and Nutrition
Lori Herbst, MD and Jennifer deSante-Bertkau, MD, MBE
Palliative Care and Medical Ethics Curriculum

## Slide 2
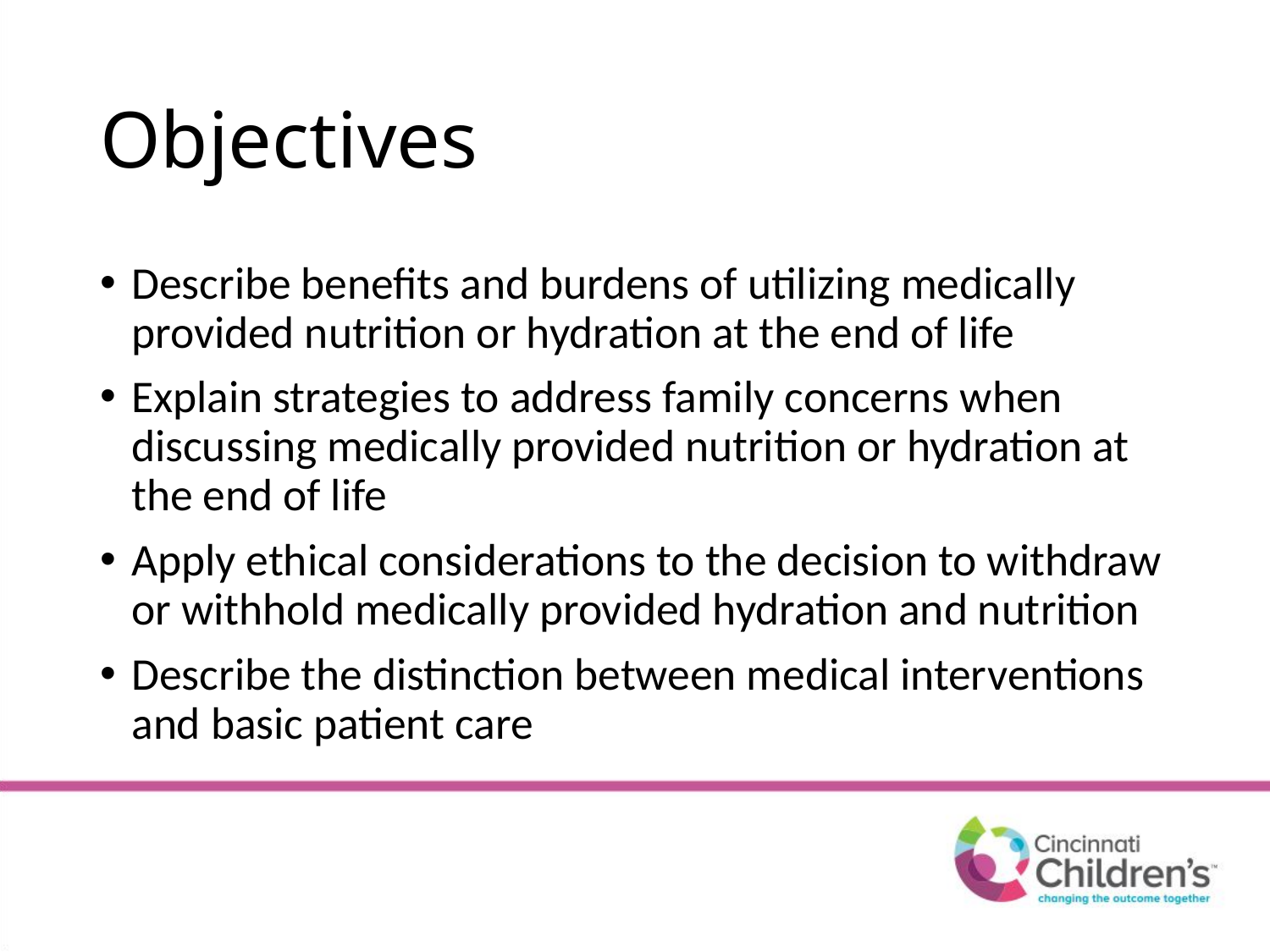

# Objectives
Describe benefits and burdens of utilizing medically provided nutrition or hydration at the end of life
Explain strategies to address family concerns when discussing medically provided nutrition or hydration at the end of life
Apply ethical considerations to the decision to withdraw or withhold medically provided hydration and nutrition
Describe the distinction between medical interventions and basic patient care

## Slide 3
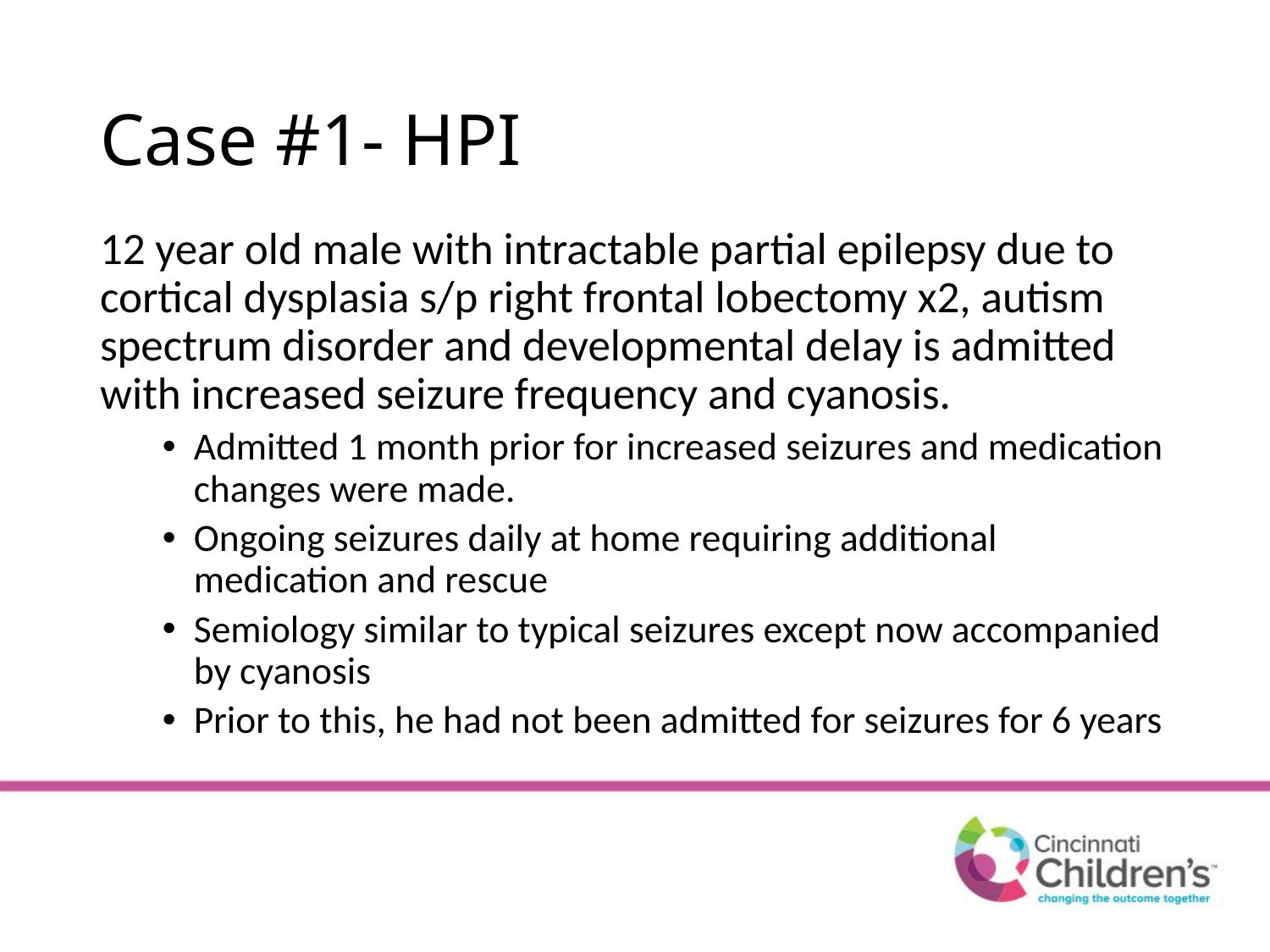

# Case #1- HPI
12 year old male with intractable partial epilepsy due to cortical dysplasia s/p right frontal lobectomy x2, autism spectrum disorder and developmental delay is admitted with increased seizure frequency and cyanosis.
Admitted 1 month prior for increased seizures and medication changes were made.
Ongoing seizures daily at home requiring additional medication and rescue
Semiology similar to typical seizures except now accompanied by cyanosis
Prior to this, he had not been admitted for seizures for 6 years

## Slide 4
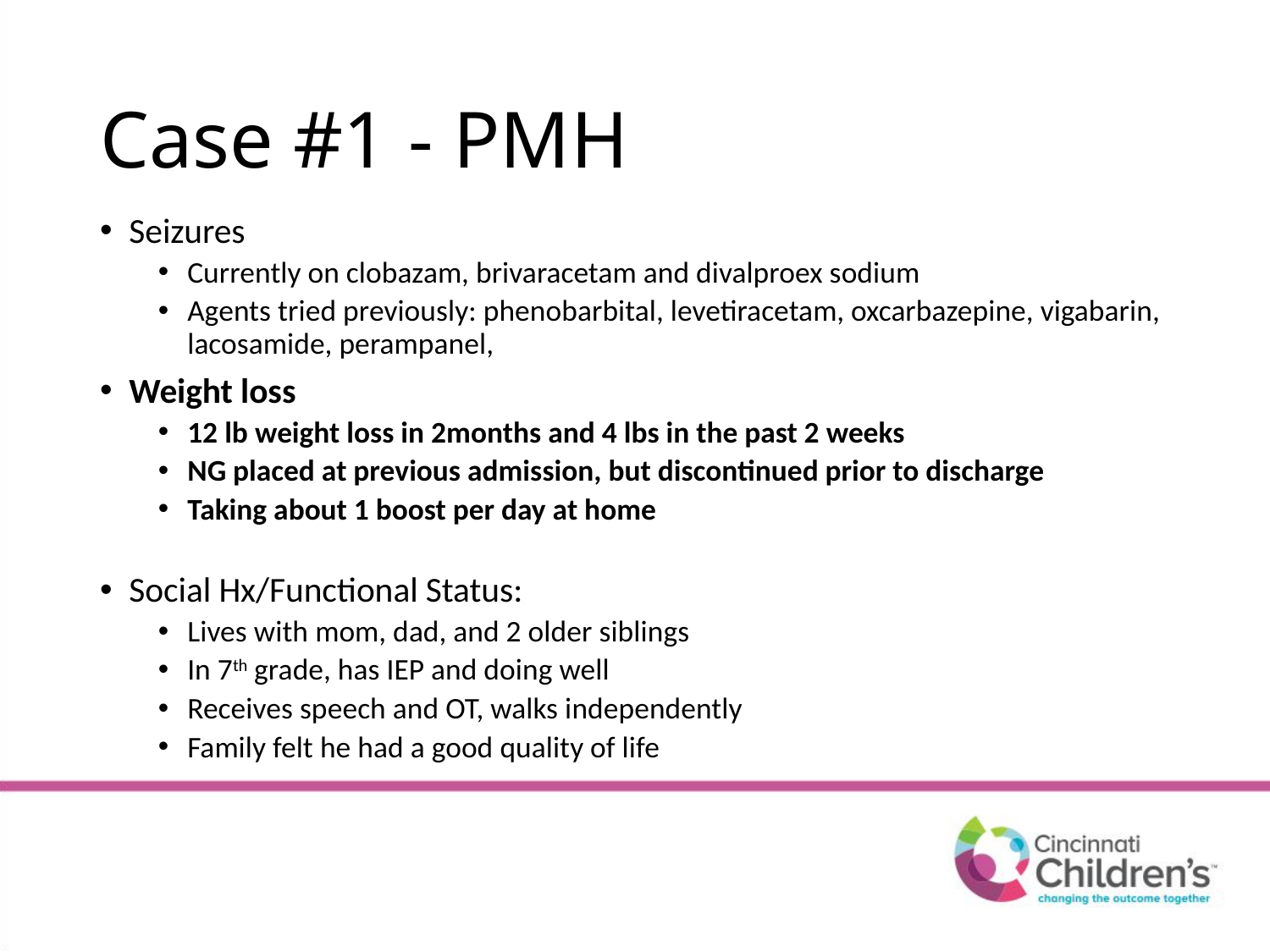

# Case #1 - PMH
Seizures
Currently on clobazam, brivaracetam and divalproex sodium
Agents tried previously: phenobarbital, levetiracetam, oxcarbazepine, vigabarin, lacosamide, perampanel,
Weight loss
12 lb weight loss in 2months and 4 lbs in the past 2 weeks
NG placed at previous admission, but discontinued prior to discharge
Taking about 1 boost per day at home
Social Hx/Functional Status:
Lives with mom, dad, and 2 older siblings
In 7th grade, has IEP and doing well
Receives speech and OT, walks independently
Family felt he had a good quality of life

## Slide 5
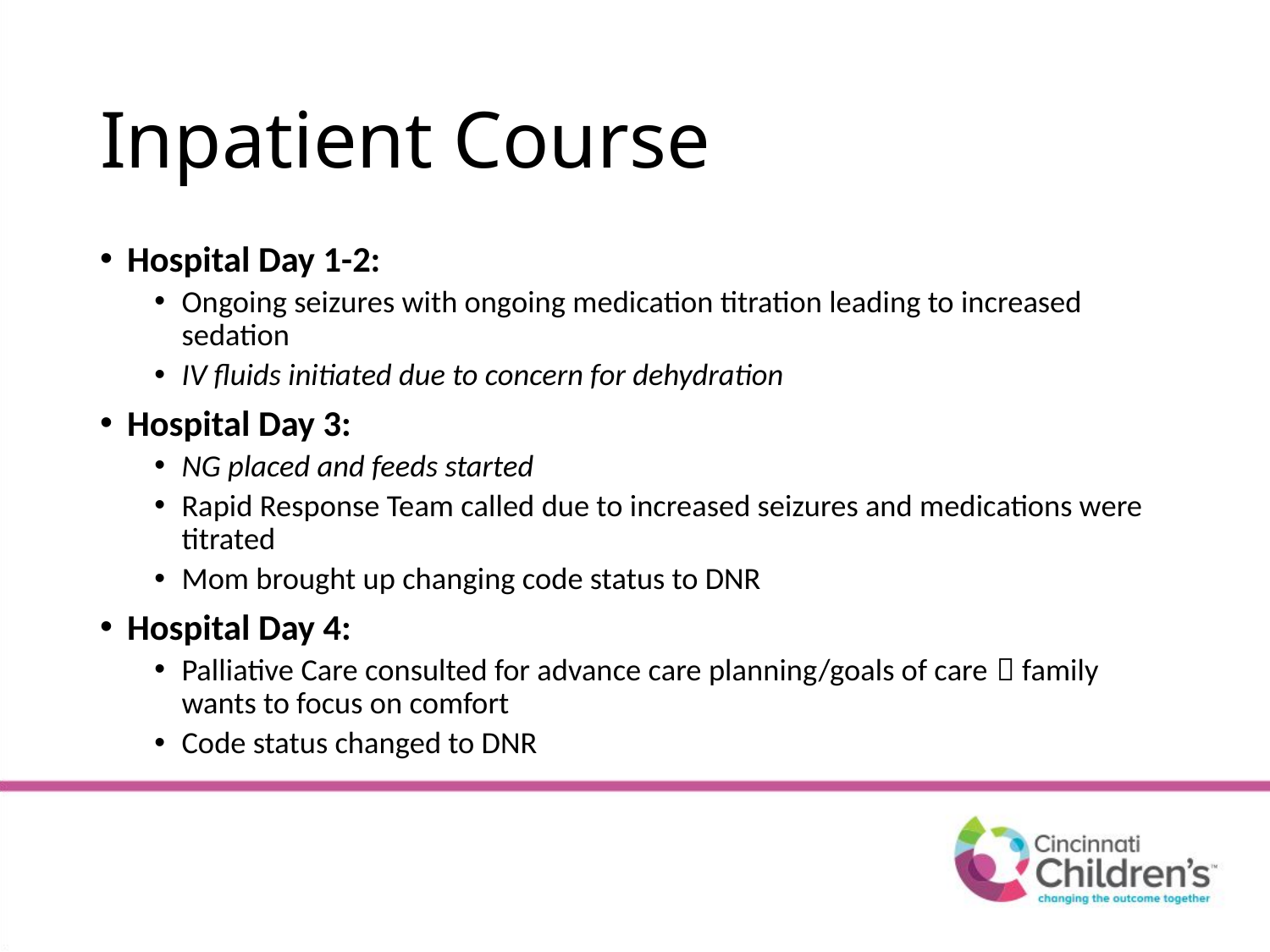

# Inpatient Course
Hospital Day 1-2:
Ongoing seizures with ongoing medication titration leading to increased sedation
IV fluids initiated due to concern for dehydration
Hospital Day 3:
NG placed and feeds started
Rapid Response Team called due to increased seizures and medications were titrated
Mom brought up changing code status to DNR
Hospital Day 4:
Palliative Care consulted for advance care planning/goals of care  family wants to focus on comfort
Code status changed to DNR

## Slide 6
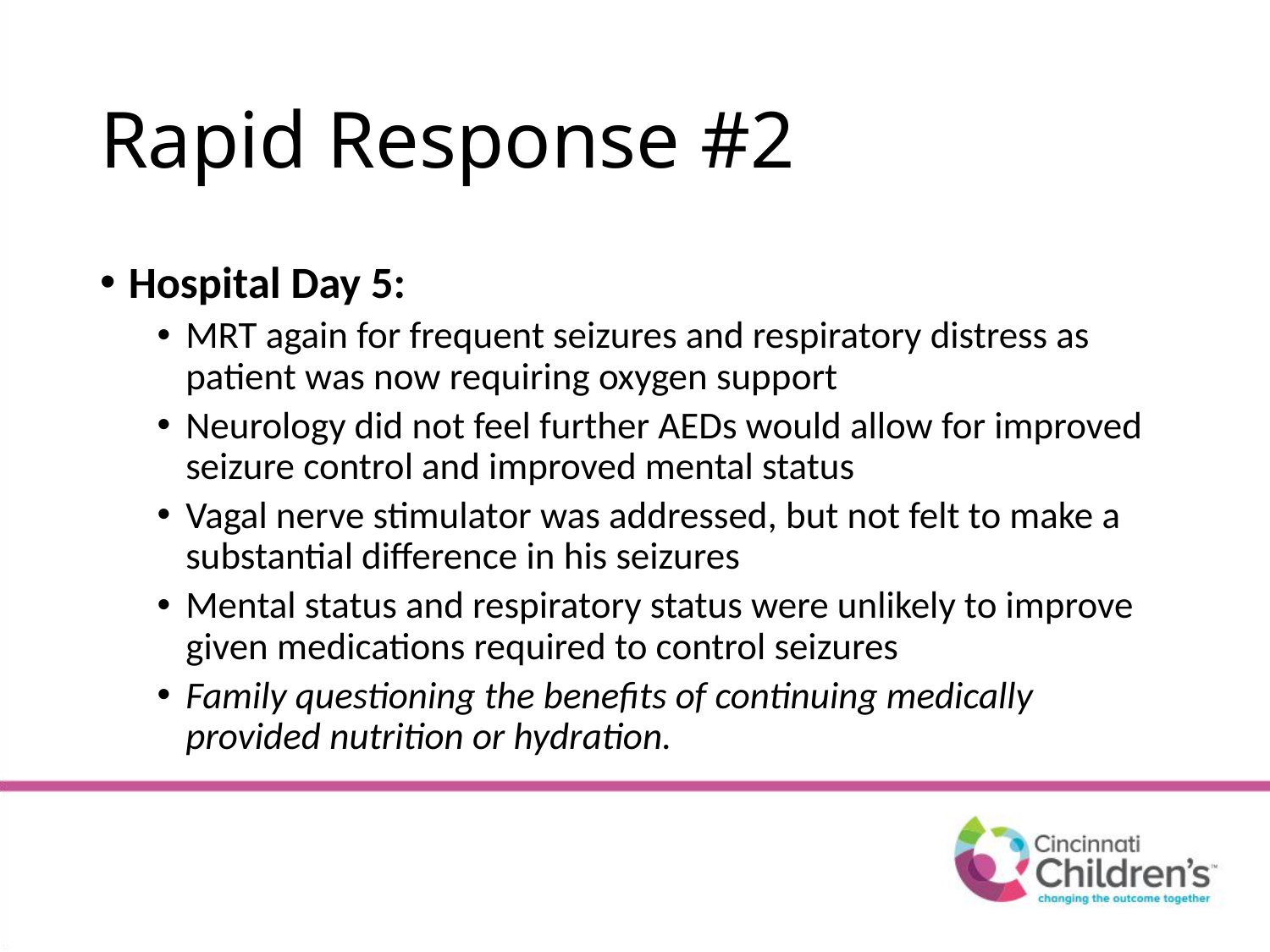

# Rapid Response #2
Hospital Day 5:
MRT again for frequent seizures and respiratory distress as patient was now requiring oxygen support
Neurology did not feel further AEDs would allow for improved seizure control and improved mental status
Vagal nerve stimulator was addressed, but not felt to make a substantial difference in his seizures
Mental status and respiratory status were unlikely to improve given medications required to control seizures
Family questioning the benefits of continuing medically provided nutrition or hydration.

## Slide 7
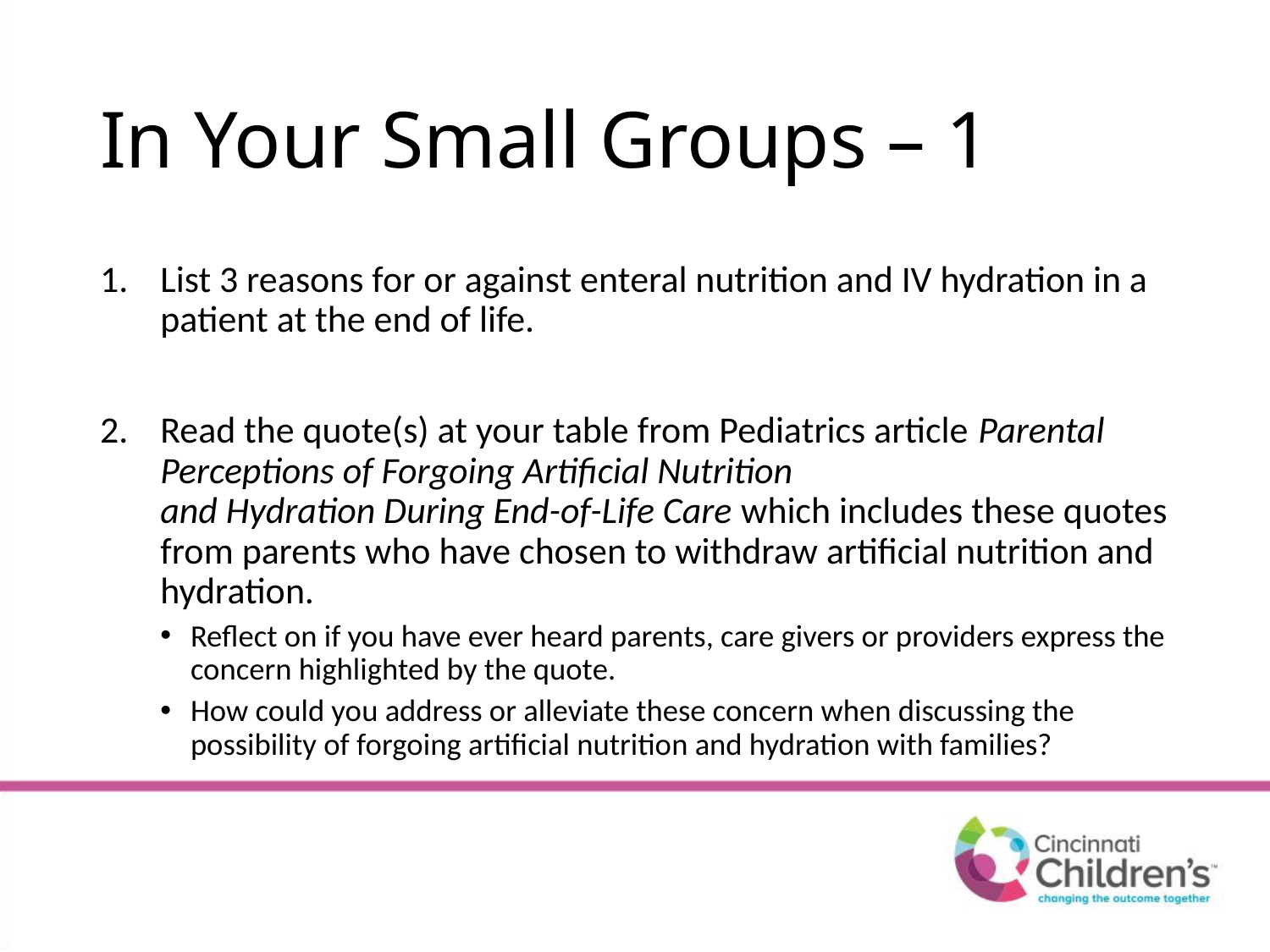

# In Your Small Groups – 1
List 3 reasons for or against enteral nutrition and IV hydration in a patient at the end of life.
Read the quote(s) at your table from Pediatrics article Parental Perceptions of Forgoing Artificial Nutrition
and Hydration During End-of-Life Care which includes these quotes from parents who have chosen to withdraw artificial nutrition and hydration.
Reflect on if you have ever heard parents, care givers or providers express the concern highlighted by the quote.
How could you address or alleviate these concern when discussing the possibility of forgoing artificial nutrition and hydration with families?

## Slide 8
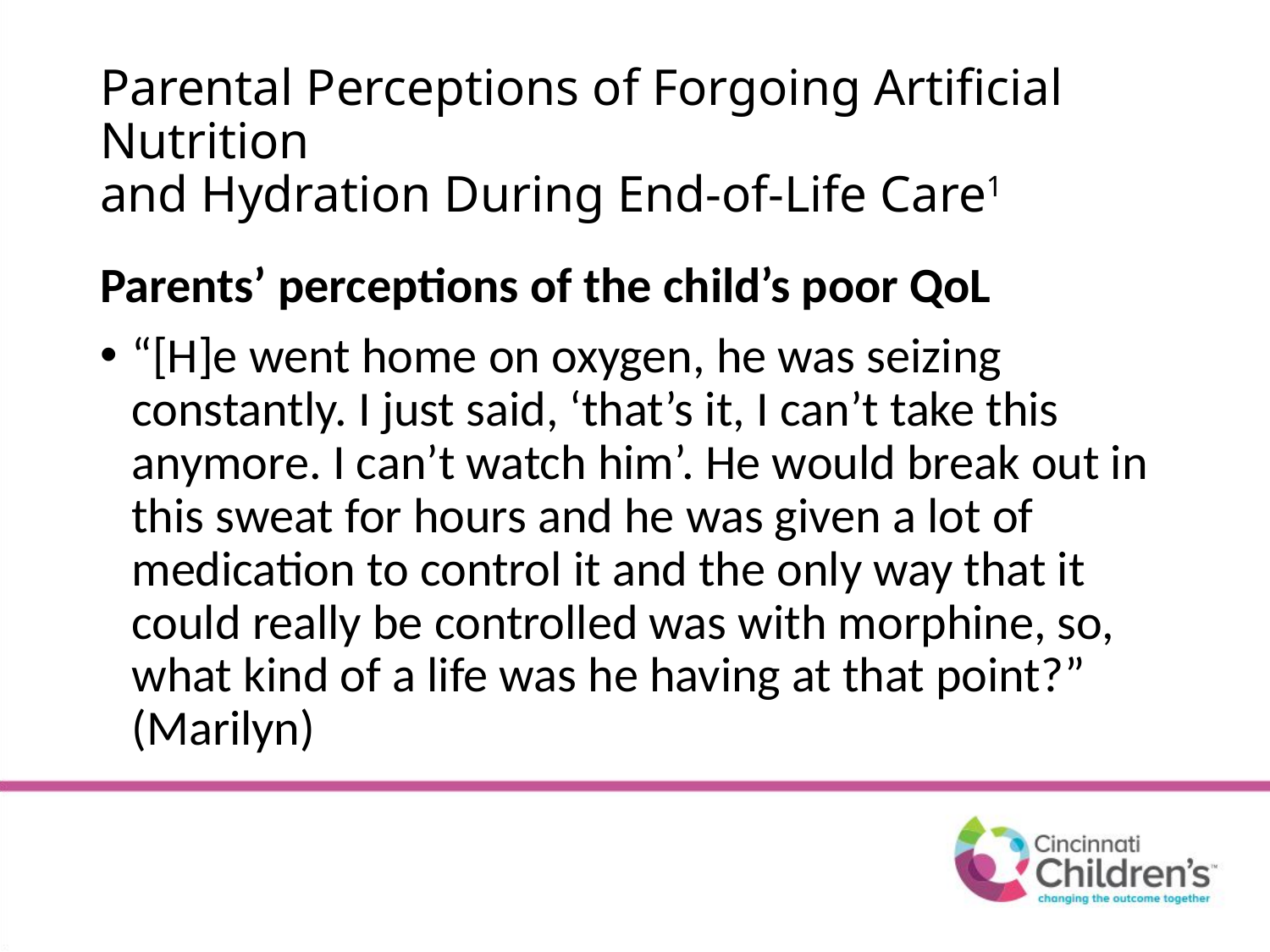

# Parental Perceptions of Forgoing Artificial Nutrition and Hydration During End-of-Life Care1
Parents’ perceptions of the child’s poor QoL
“[H]e went home on oxygen, he was seizing constantly. I just said, ‘that’s it, I can’t take this anymore. I can’t watch him’. He would break out in this sweat for hours and he was given a lot of medication to control it and the only way that it could really be controlled was with morphine, so, what kind of a life was he having at that point?” (Marilyn)

## Slide 9
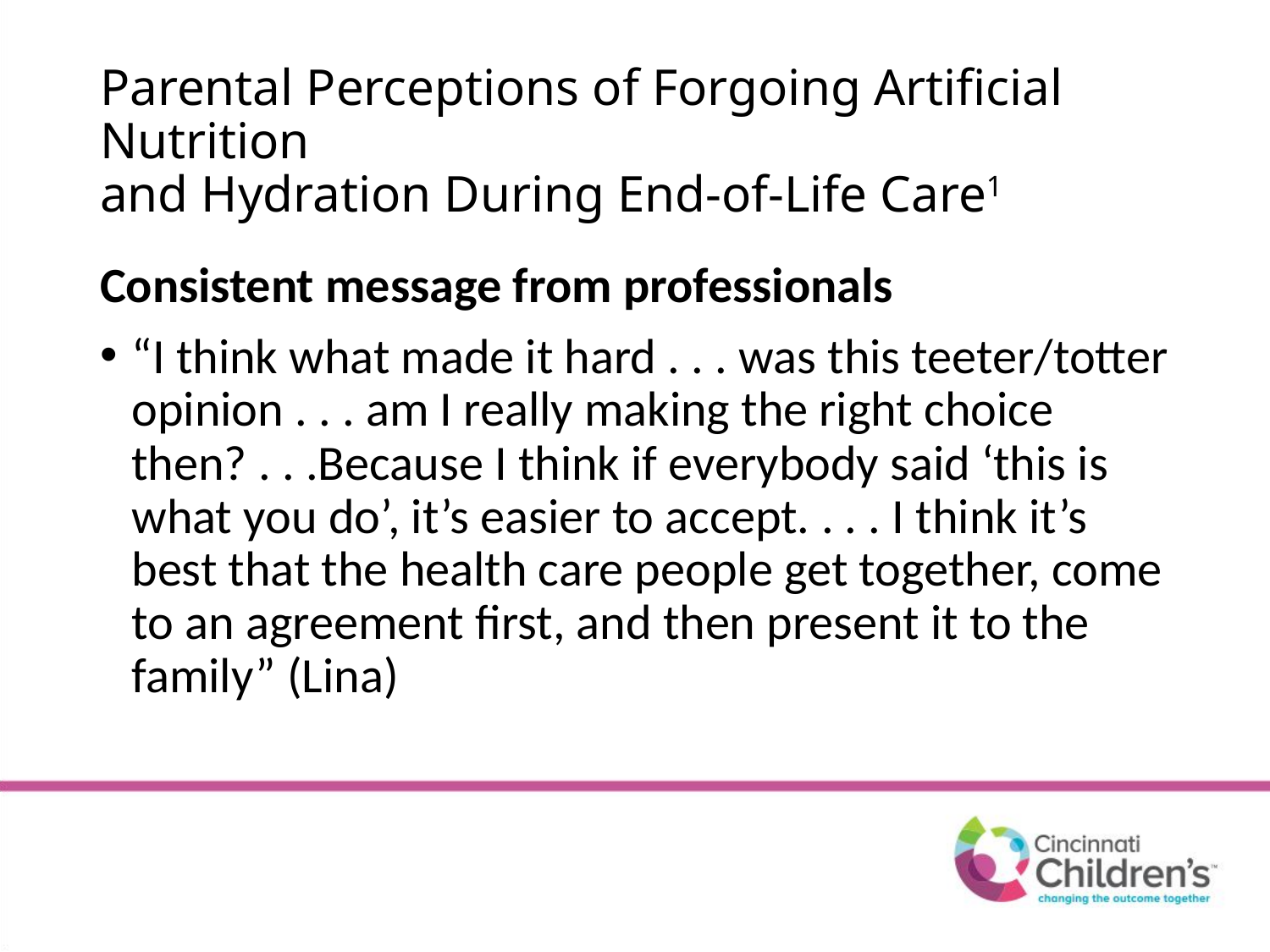

# Parental Perceptions of Forgoing Artificial Nutrition and Hydration During End-of-Life Care1
Consistent message from professionals
“I think what made it hard . . . was this teeter/totter opinion . . . am I really making the right choice then? . . .Because I think if everybody said ‘this is what you do’, it’s easier to accept. . . . I think it’s best that the health care people get together, come to an agreement first, and then present it to the family” (Lina)

## Slide 10
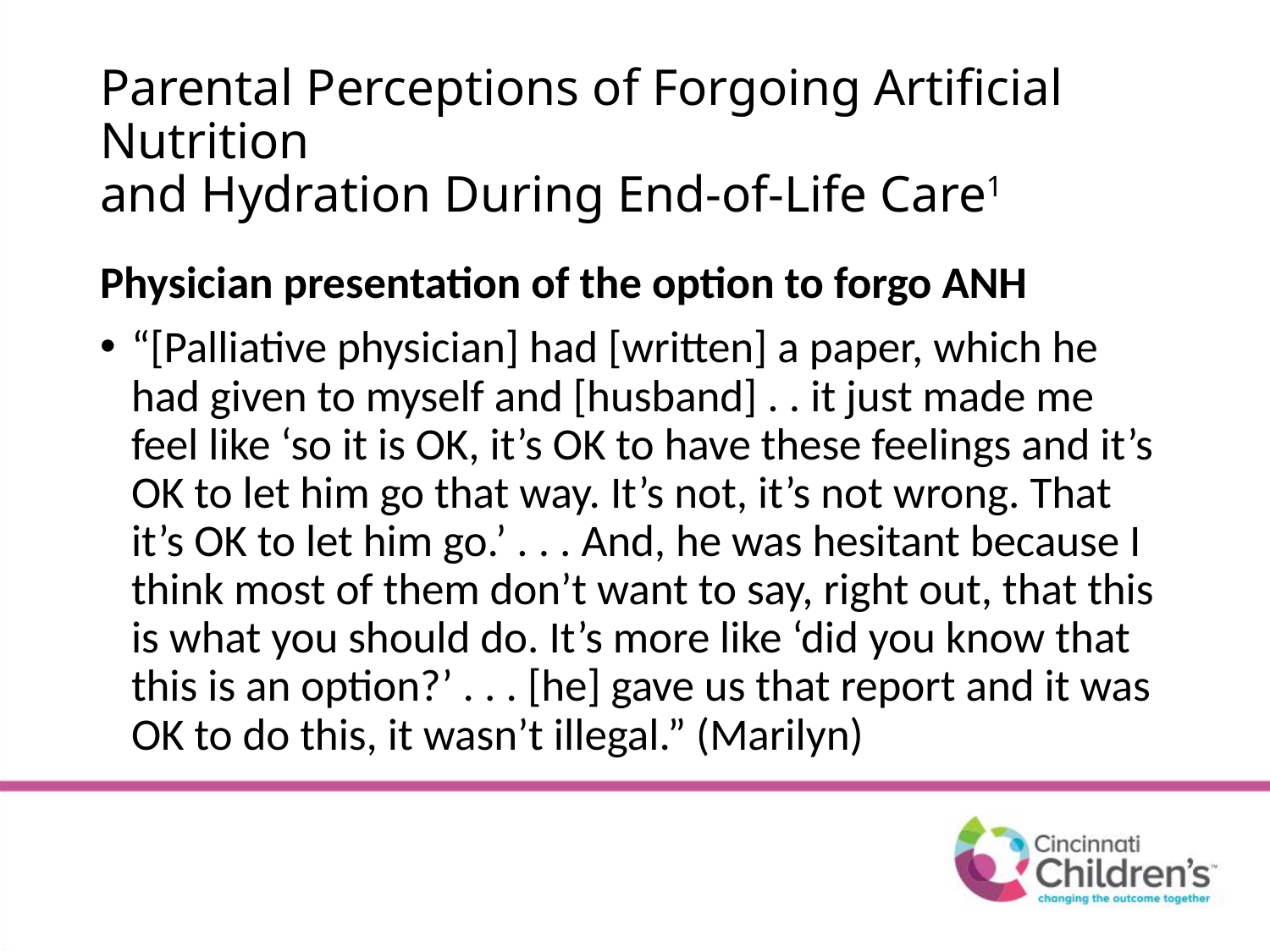

# Parental Perceptions of Forgoing Artificial Nutrition and Hydration During End-of-Life Care1
Physician presentation of the option to forgo ANH
“[Palliative physician] had [written] a paper, which he had given to myself and [husband] . . it just made me feel like ‘so it is OK, it’s OK to have these feelings and it’s OK to let him go that way. It’s not, it’s not wrong. That it’s OK to let him go.’ . . . And, he was hesitant because I think most of them don’t want to say, right out, that this is what you should do. It’s more like ‘did you know that this is an option?’ . . . [he] gave us that report and it was OK to do this, it wasn’t illegal.” (Marilyn)

## Slide 11
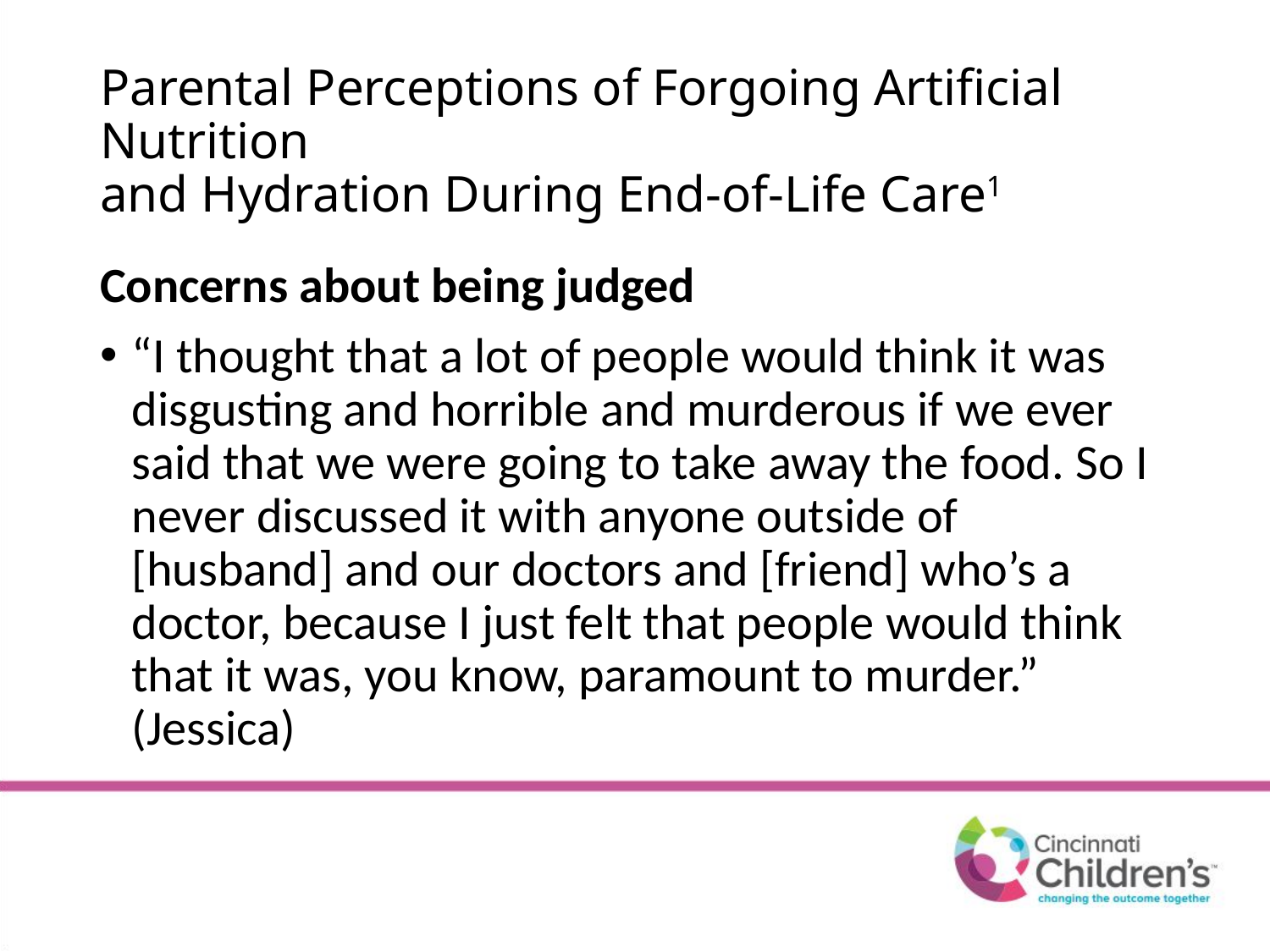

# Parental Perceptions of Forgoing Artificial Nutrition and Hydration During End-of-Life Care1
Concerns about being judged
“I thought that a lot of people would think it was disgusting and horrible and murderous if we ever said that we were going to take away the food. So I never discussed it with anyone outside of [husband] and our doctors and [friend] who’s a doctor, because I just felt that people would think that it was, you know, paramount to murder.” (Jessica)

## Slide 12
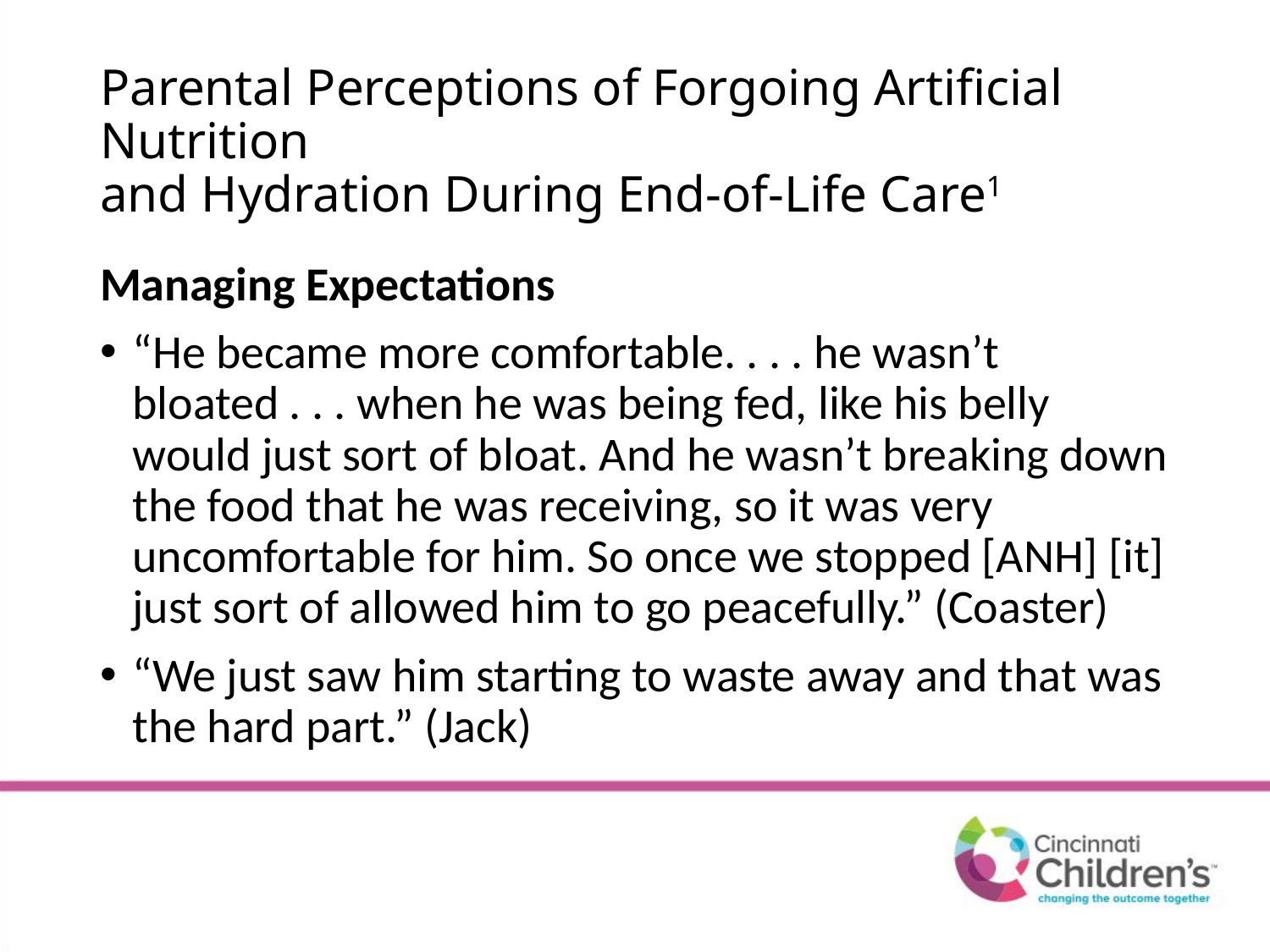

# Parental Perceptions of Forgoing Artificial Nutrition and Hydration During End-of-Life Care1
Managing Expectations
“He became more comfortable. . . . he wasn’t bloated . . . when he was being fed, like his belly would just sort of bloat. And he wasn’t breaking down the food that he was receiving, so it was very uncomfortable for him. So once we stopped [ANH] [it] just sort of allowed him to go peacefully.” (Coaster)
“We just saw him starting to waste away and that was the hard part.” (Jack)

## Slide 13
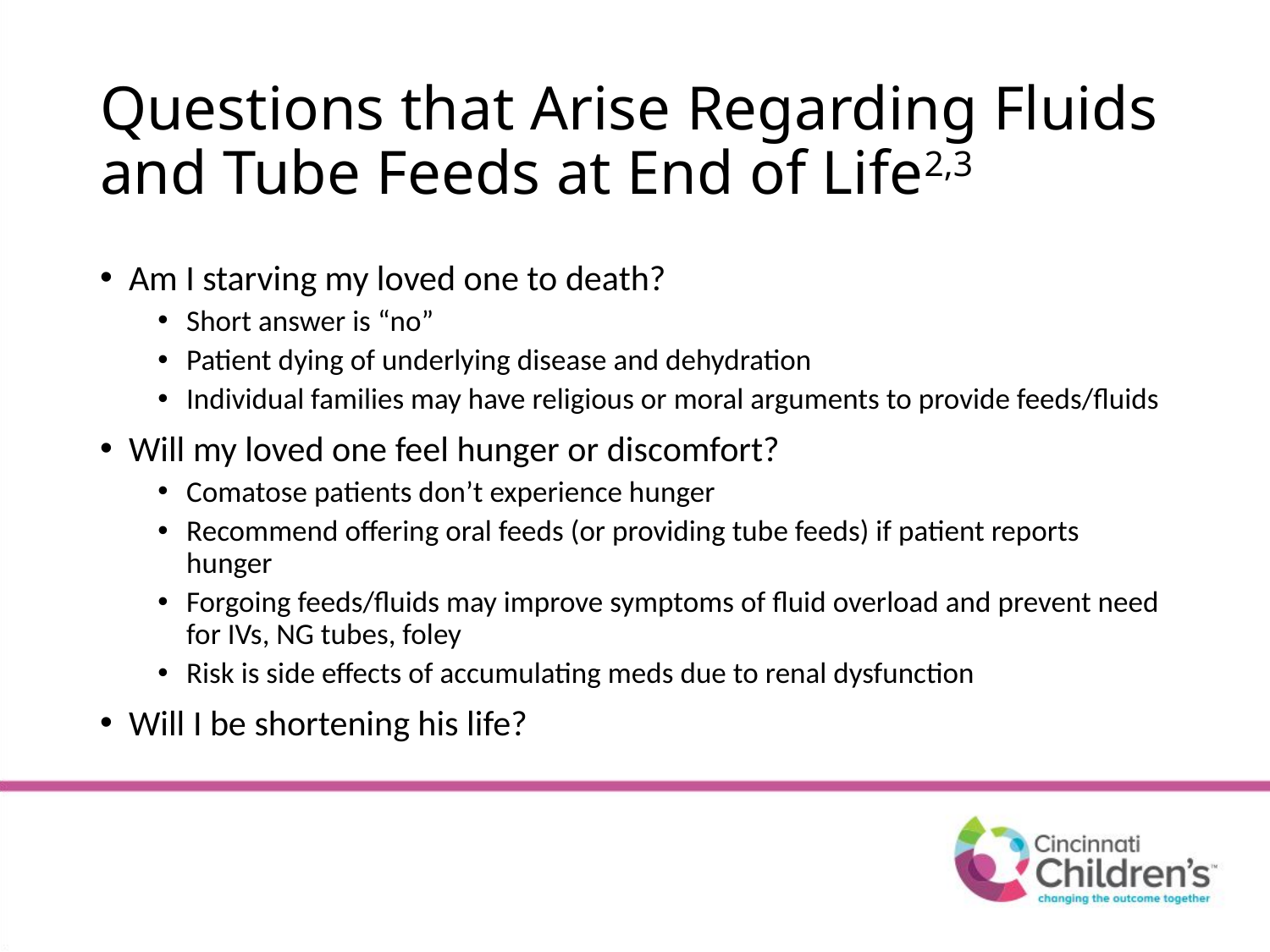

# Questions that Arise Regarding Fluids and Tube Feeds at End of Life2,3
Am I starving my loved one to death?
Short answer is “no”
Patient dying of underlying disease and dehydration
Individual families may have religious or moral arguments to provide feeds/fluids
Will my loved one feel hunger or discomfort?
Comatose patients don’t experience hunger
Recommend offering oral feeds (or providing tube feeds) if patient reports hunger
Forgoing feeds/fluids may improve symptoms of fluid overload and prevent need for IVs, NG tubes, foley
Risk is side effects of accumulating meds due to renal dysfunction
Will I be shortening his life?

## Slide 14
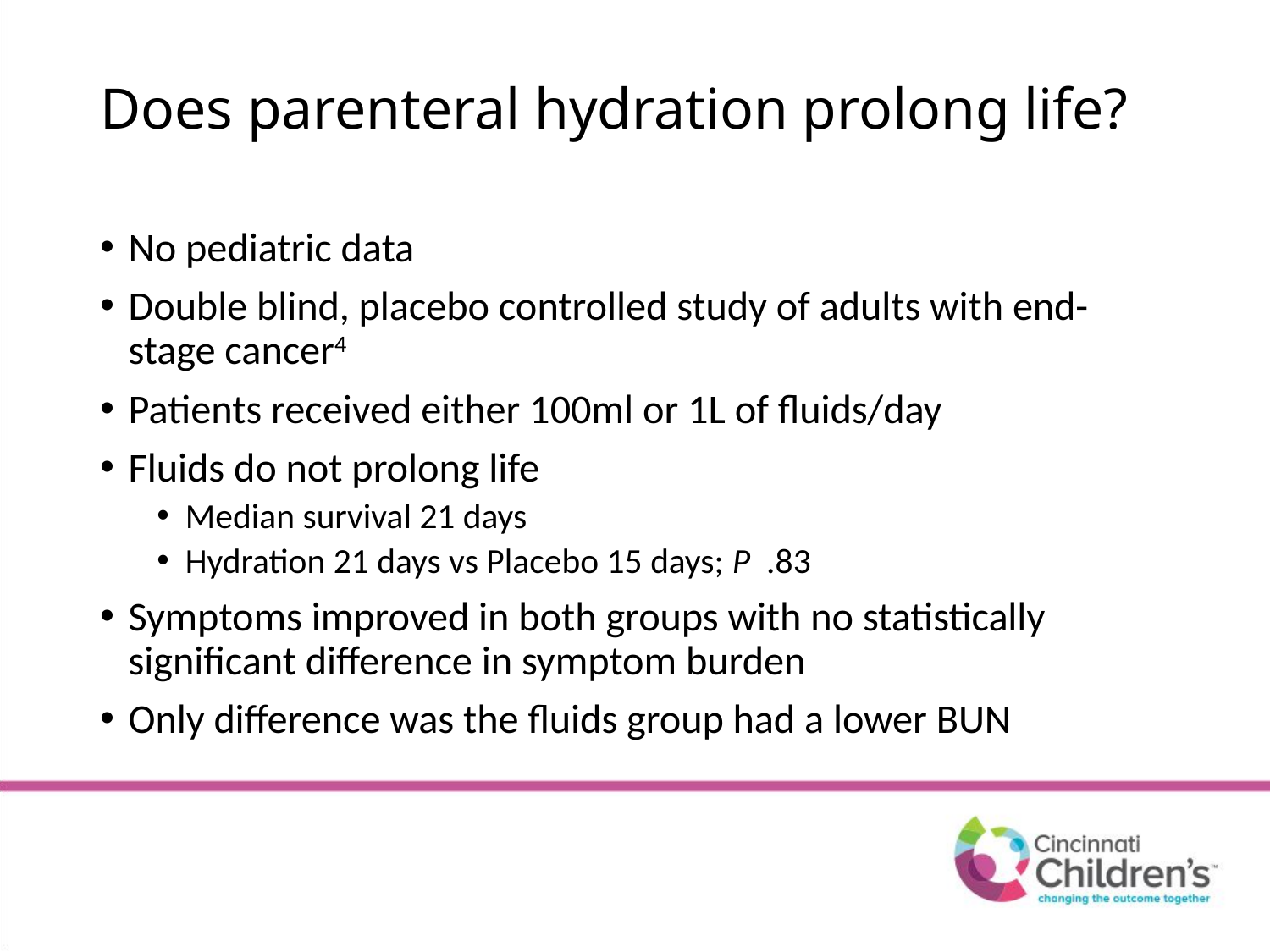

# Does parenteral hydration prolong life?
No pediatric data
Double blind, placebo controlled study of adults with end-stage cancer4
Patients received either 100ml or 1L of fluids/day
Fluids do not prolong life
Median survival 21 days
Hydration 21 days vs Placebo 15 days; P .83
Symptoms improved in both groups with no statistically significant difference in symptom burden
Only difference was the fluids group had a lower BUN

## Slide 15
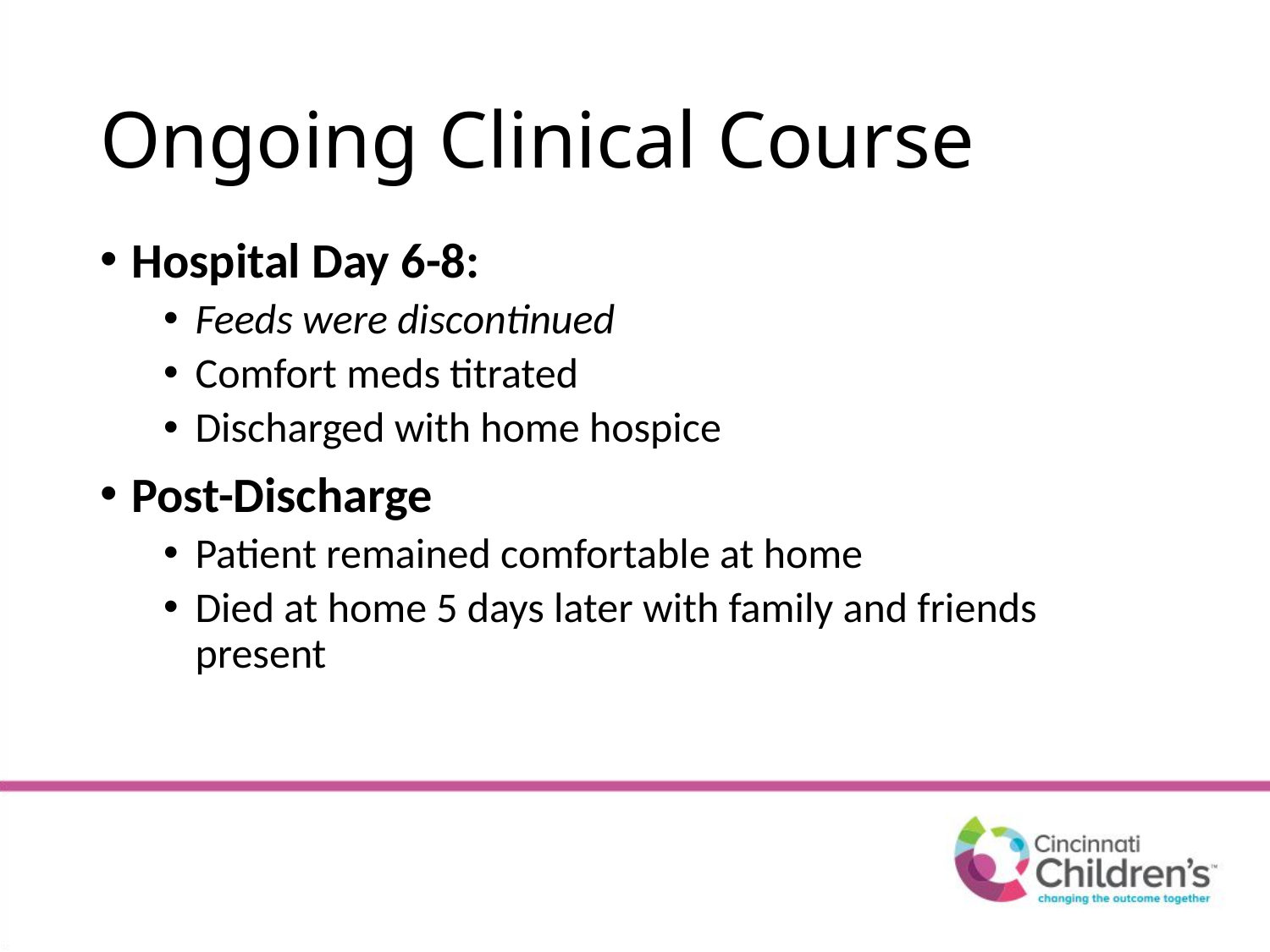

# Ongoing Clinical Course
Hospital Day 6-8:
Feeds were discontinued
Comfort meds titrated
Discharged with home hospice
Post-Discharge
Patient remained comfortable at home
Died at home 5 days later with family and friends present

## Slide 16
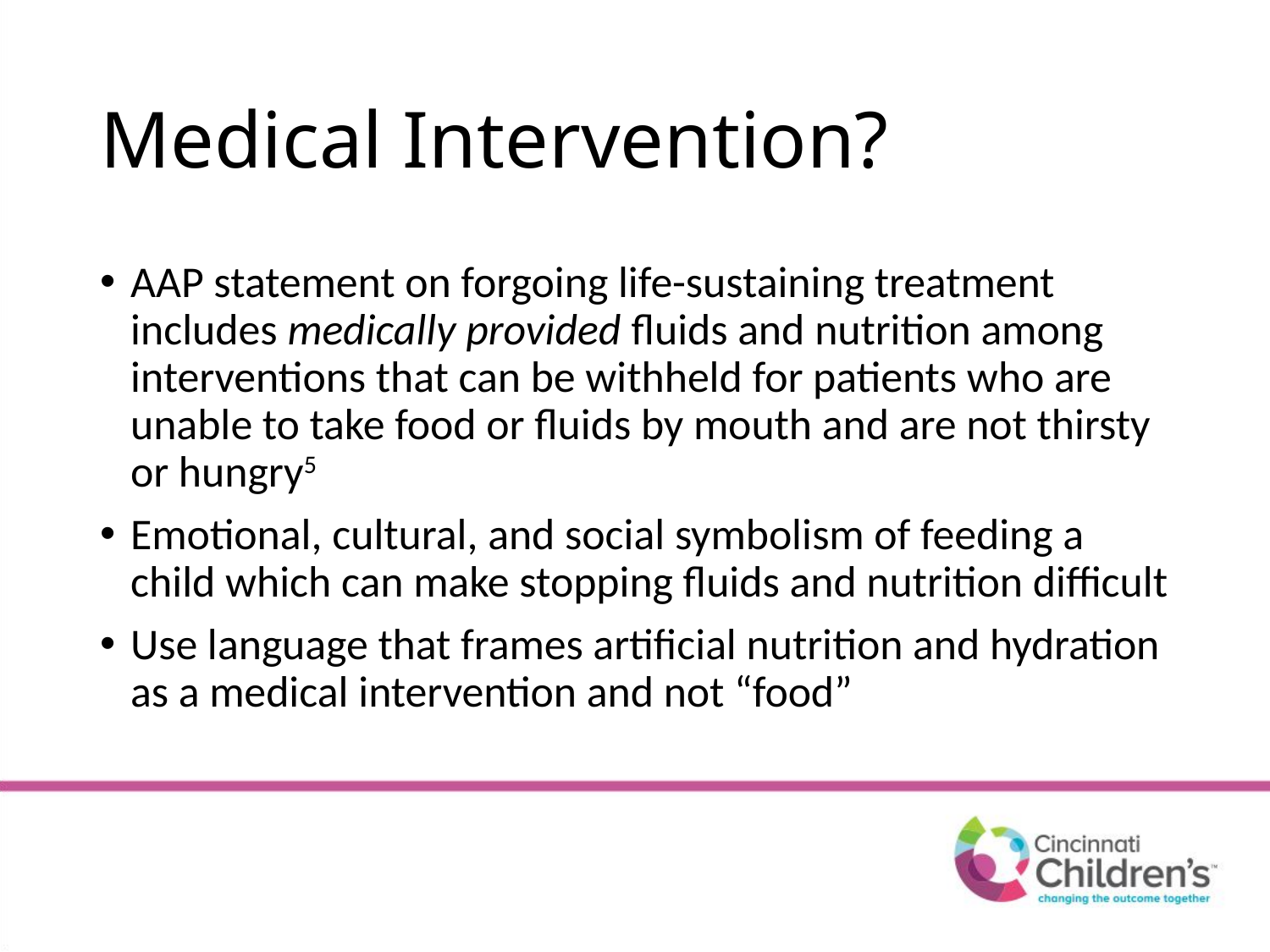

# Medical Intervention?
AAP statement on forgoing life-sustaining treatment includes medically provided fluids and nutrition among interventions that can be withheld for patients who are unable to take food or fluids by mouth and are not thirsty or hungry5
Emotional, cultural, and social symbolism of feeding a child which can make stopping fluids and nutrition difficult
Use language that frames artificial nutrition and hydration as a medical intervention and not “food”

## Slide 17
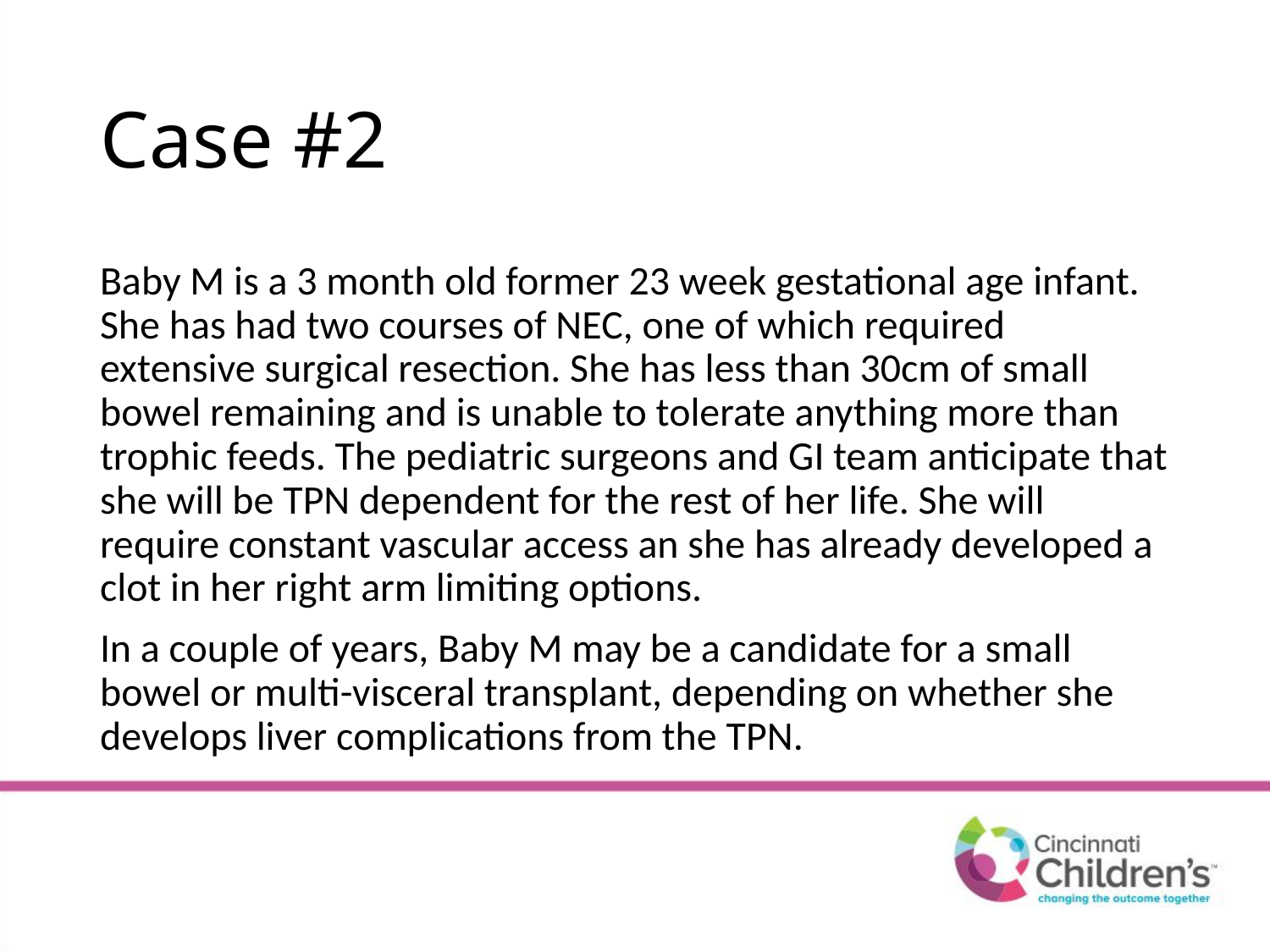

# Case #2
Baby M is a 3 month old former 23 week gestational age infant. She has had two courses of NEC, one of which required extensive surgical resection. She has less than 30cm of small bowel remaining and is unable to tolerate anything more than trophic feeds. The pediatric surgeons and GI team anticipate that she will be TPN dependent for the rest of her life. She will require constant vascular access an she has already developed a clot in her right arm limiting options.
In a couple of years, Baby M may be a candidate for a small bowel or multi-visceral transplant, depending on whether she develops liver complications from the TPN.

## Slide 18
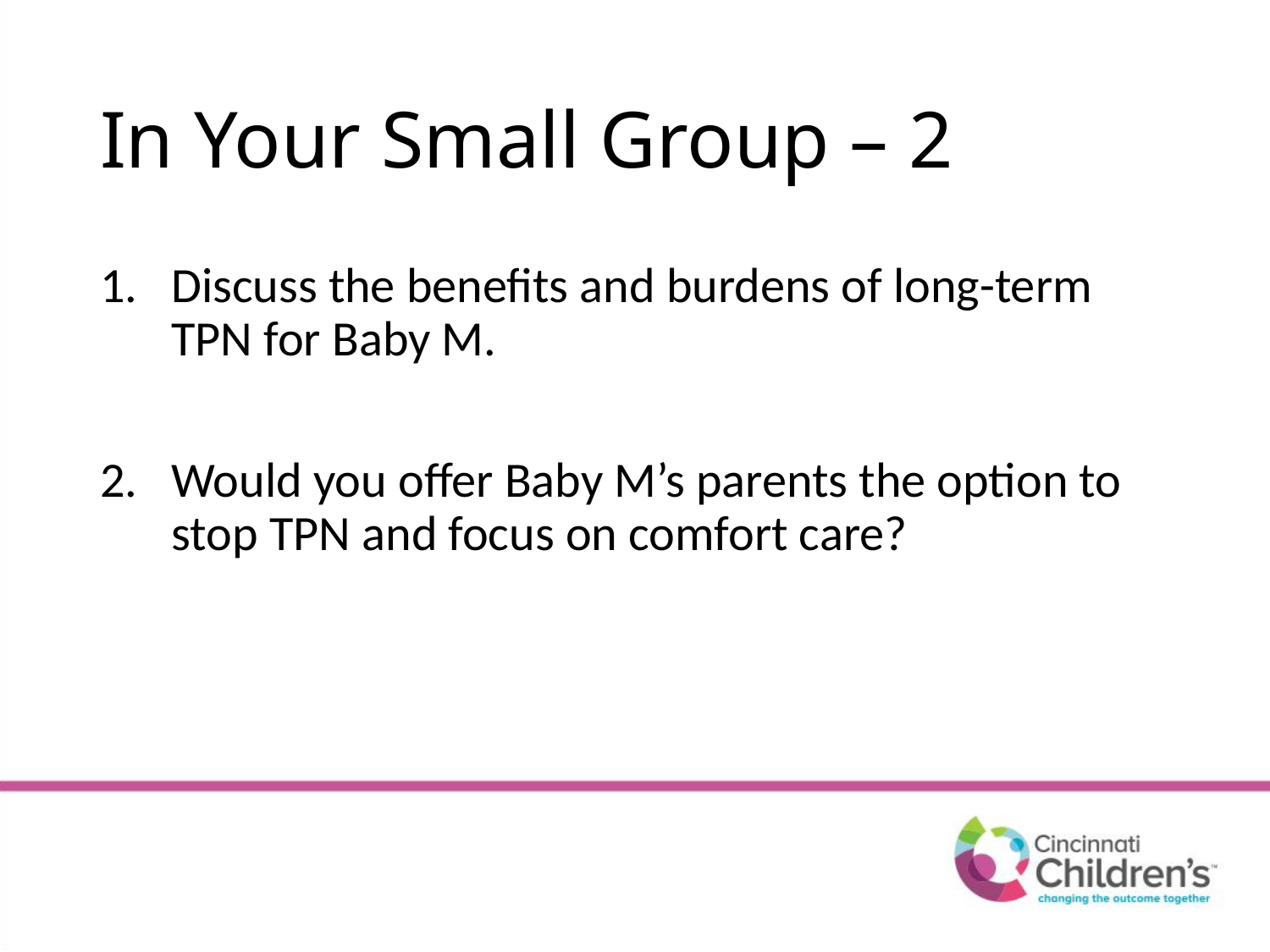

# In Your Small Group – 2
Discuss the benefits and burdens of long-term TPN for Baby M.
Would you offer Baby M’s parents the option to stop TPN and focus on comfort care?

## Slide 19
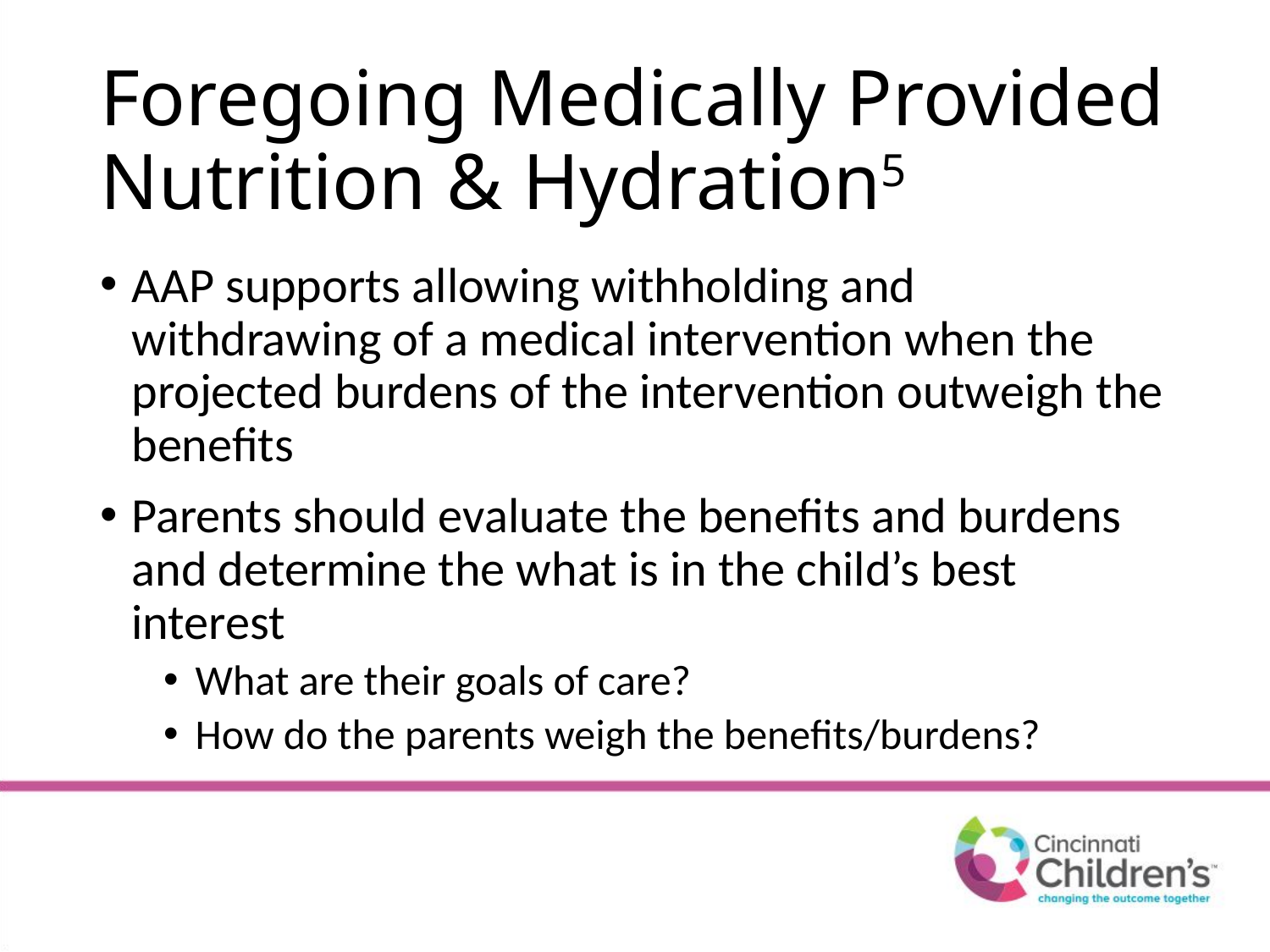

# Foregoing Medically Provided Nutrition & Hydration5
AAP supports allowing withholding and withdrawing of a medical intervention when the projected burdens of the intervention outweigh the benefits
Parents should evaluate the benefits and burdens and determine the what is in the child’s best interest
What are their goals of care?
How do the parents weigh the benefits/burdens?

## Slide 20
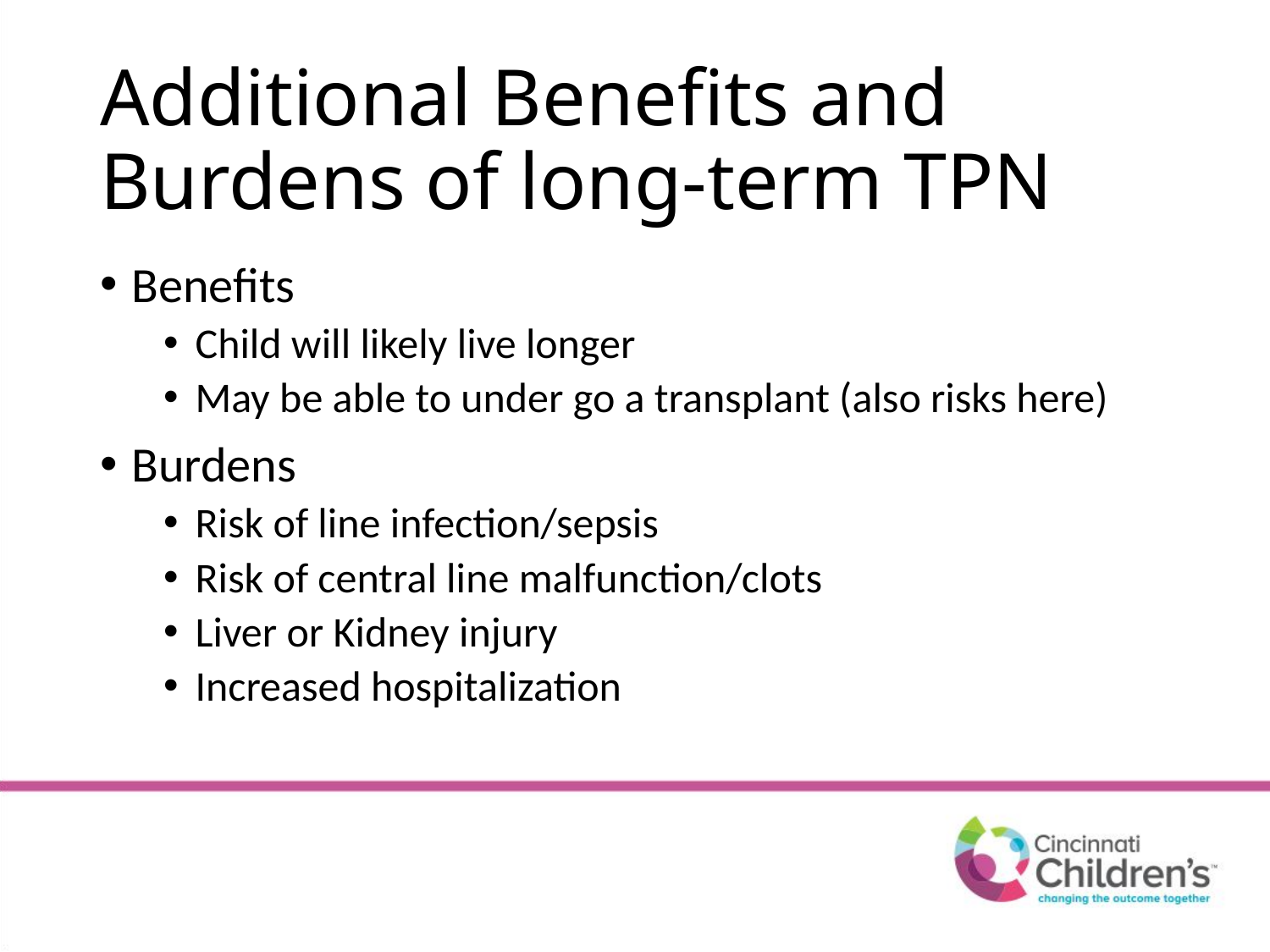

# Additional Benefits and Burdens of long-term TPN
Benefits
Child will likely live longer
May be able to under go a transplant (also risks here)
Burdens
Risk of line infection/sepsis
Risk of central line malfunction/clots
Liver or Kidney injury
Increased hospitalization

## Slide 21
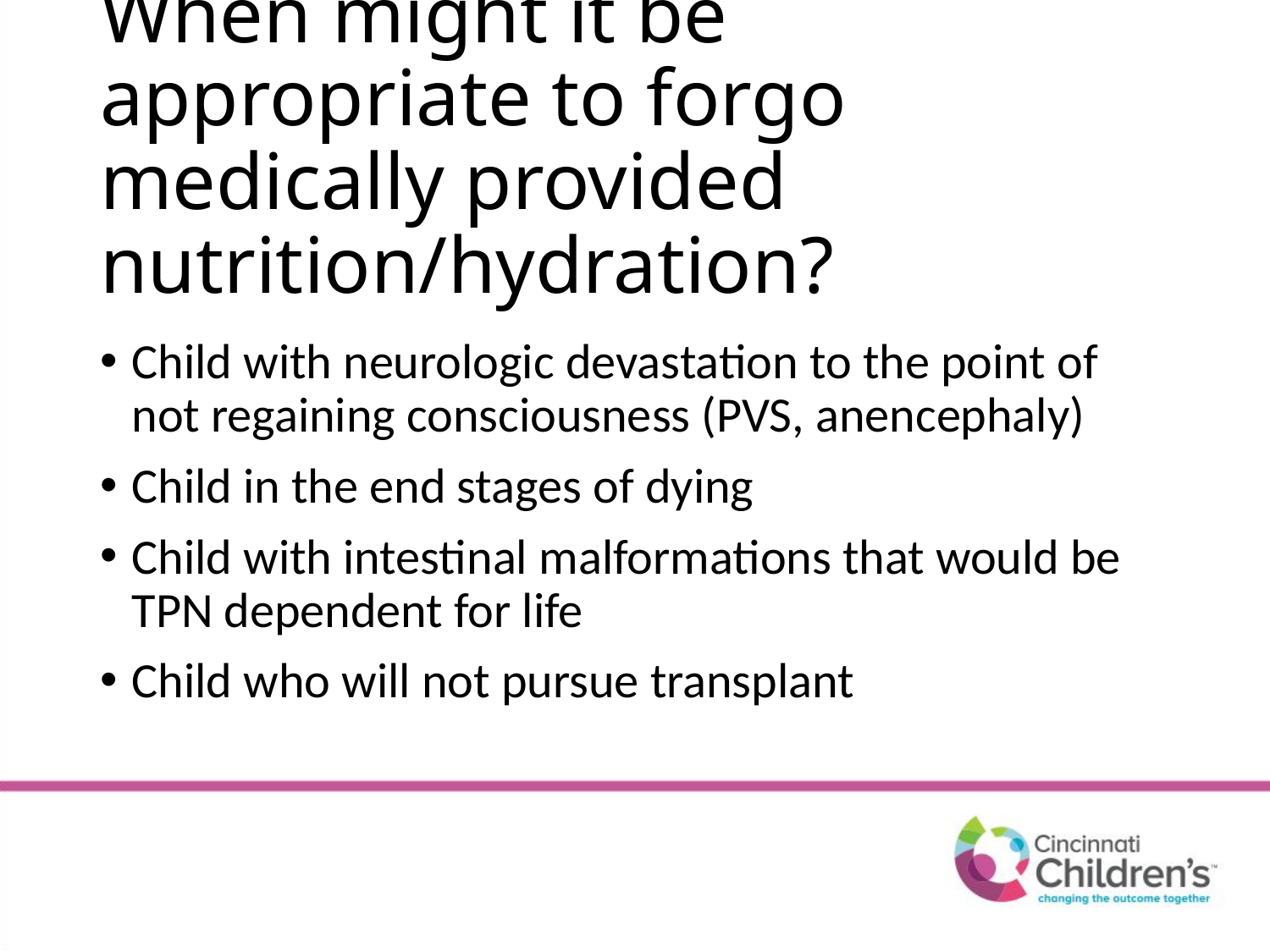

# When might it be appropriate to forgo medically provided nutrition/hydration?
Child with neurologic devastation to the point of not regaining consciousness (PVS, anencephaly)
Child in the end stages of dying
Child with intestinal malformations that would be TPN dependent for life
Child who will not pursue transplant

## Slide 22
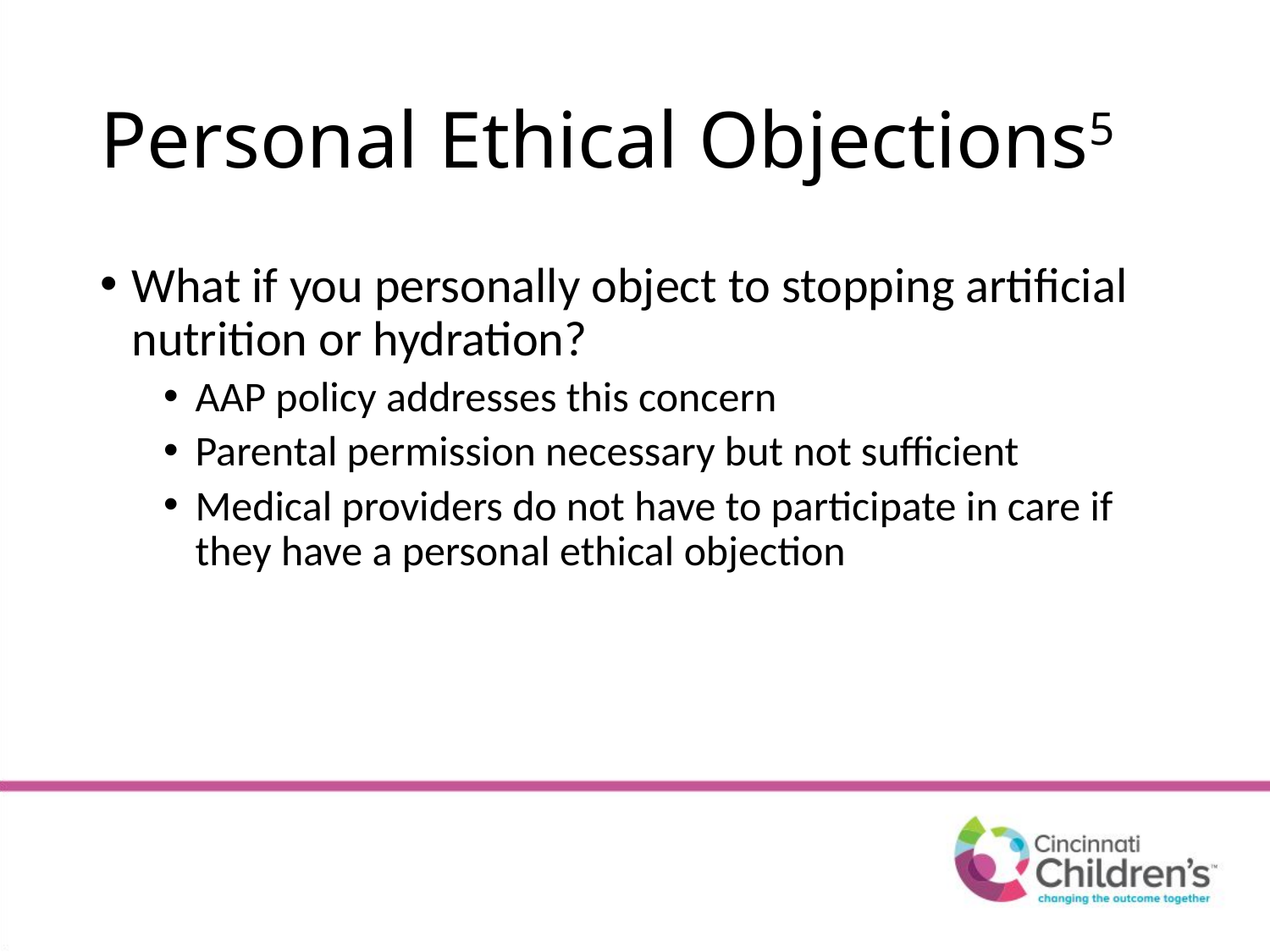

# Personal Ethical Objections5
What if you personally object to stopping artificial nutrition or hydration?
AAP policy addresses this concern
Parental permission necessary but not sufficient
Medical providers do not have to participate in care if they have a personal ethical objection

## Slide 23
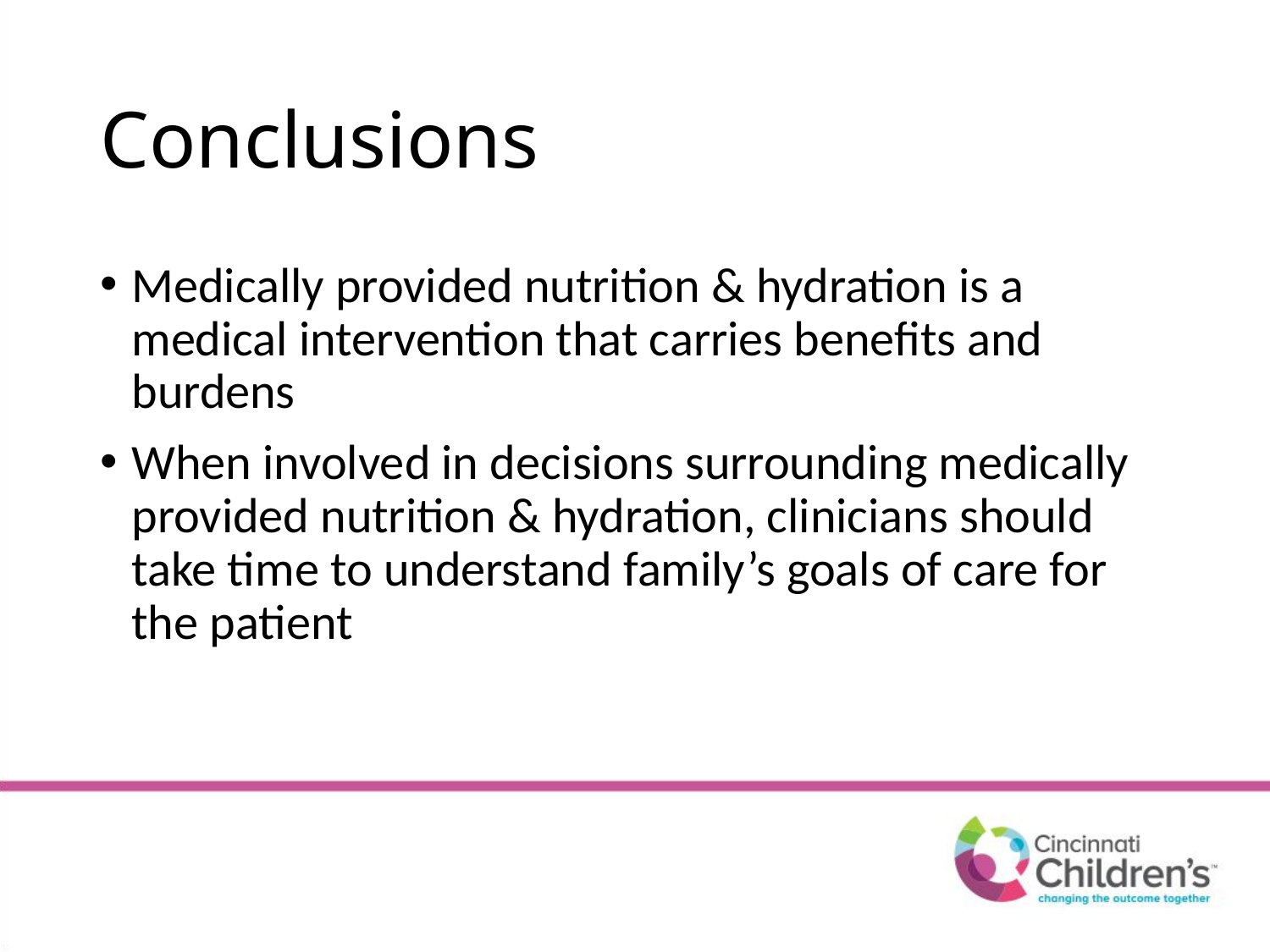

# Conclusions
Medically provided nutrition & hydration is a medical intervention that carries benefits and burdens
When involved in decisions surrounding medically provided nutrition & hydration, clinicians should take time to understand family’s goals of care for the patient

## Slide 24
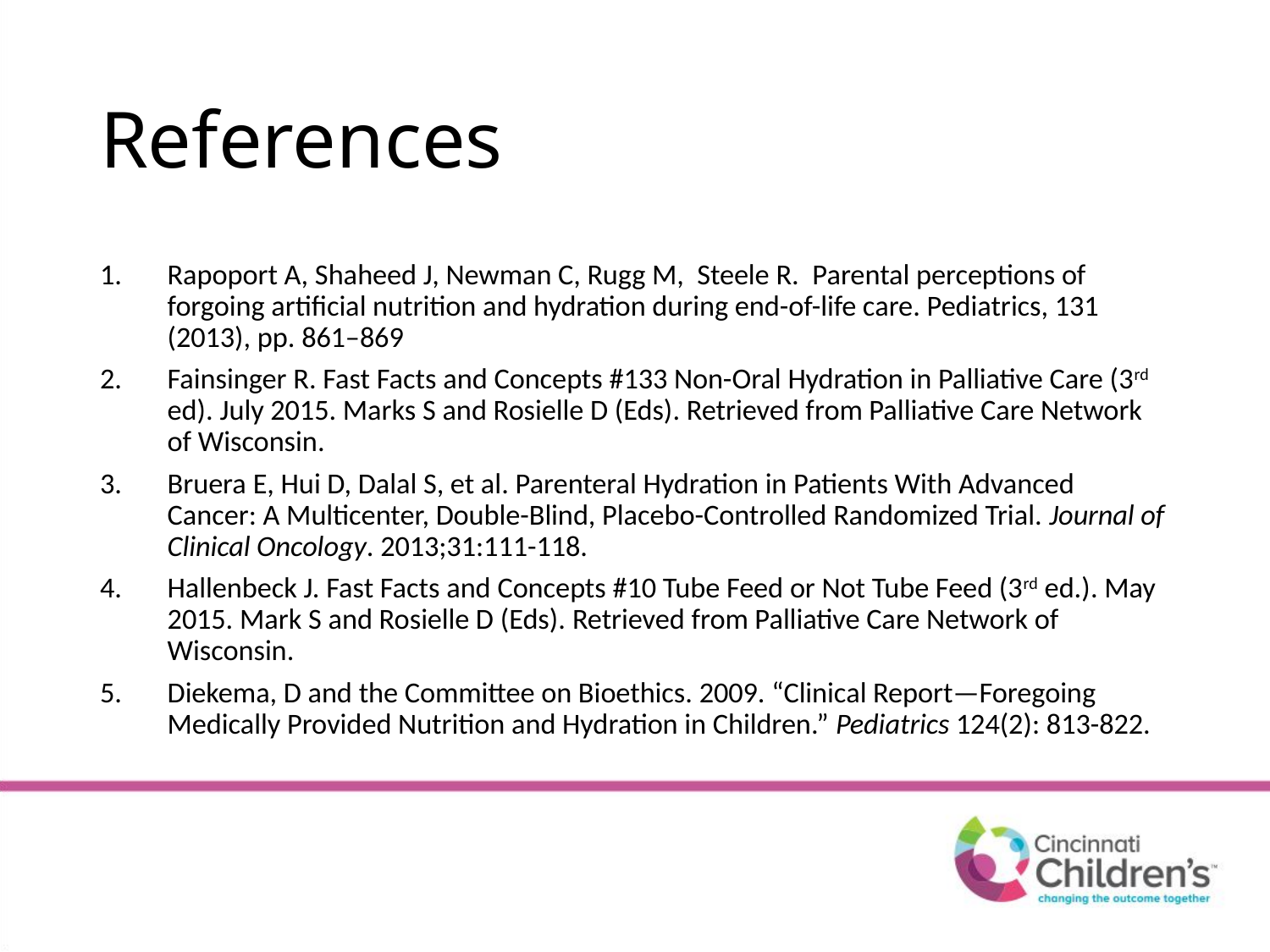

# References
Rapoport A, Shaheed J, Newman C, Rugg M, Steele R. Parental perceptions of forgoing artificial nutrition and hydration during end-of-life care. Pediatrics, 131 (2013), pp. 861–869
Fainsinger R. Fast Facts and Concepts #133 Non-Oral Hydration in Palliative Care (3rd ed). July 2015. Marks S and Rosielle D (Eds). Retrieved from Palliative Care Network of Wisconsin.
Bruera E, Hui D, Dalal S, et al. Parenteral Hydration in Patients With Advanced Cancer: A Multicenter, Double-Blind, Placebo-Controlled Randomized Trial. Journal of Clinical Oncology. 2013;31:111-118.
Hallenbeck J. Fast Facts and Concepts #10 Tube Feed or Not Tube Feed (3rd ed.). May 2015. Mark S and Rosielle D (Eds). Retrieved from Palliative Care Network of Wisconsin.
Diekema, D and the Committee on Bioethics. 2009. “Clinical Report—Foregoing Medically Provided Nutrition and Hydration in Children.” Pediatrics 124(2): 813-822.
