# Supplementary material for: Ethical Dilemmas at the Beginning and End of Life: A Needs-Based, Experience-Informed, Small-Group, Case-Based Curriculum for Pediatric Residents
Source: MedEdPORTAL. 2020 Apr 3;16:10895. doi: 10.15766/mep_2374-8265.10895 (PMC7187913; doi:10.15766/mep_2374-8265.10895)
Supplement: Supplementary file 1 — Medically Provided Fluids Nutrition PowerPoint.pptxMedically Provided Fluids Nutrition Instructor Guide.docxMedically Provided Fluids Nutrition Handout.docxMedically Provided Fluids Nutrition Assessment Questions.docxFutility and Goals of Care PowerPoint.pptxFutility and Goals of Care Instructor Guide.docxFutility and Goals of Care Handout.docxFutility and Goals of Care Assessment Questions.docxEthical Issues in Neonatology PowerPoint.pptxEthical Issues in Neonatology Instructor Guide.docxEthical Issues in Neonatology Assessment Questions.docx [file mep-16-10895-s001.zip › B. Medically Provided Fluids Nutrition Instructor Guide.docx]

**Medically Provided Fluid and Nutrition
Instructor Directions**

*Set-up:*

- Table Set-up: The group leader should arrange the classroom in multiple individual tables with seats for 6-10 learners.
- Learner Mix: If this is a mixed group of learners (i.e. medical students, interns, upper level residents), there should be a mix of all learners at each table. If possible, an attending physician who has encountered this or a similar challenge, should also be present at the table to provide additional guidance.
- Assessment Tools: Pre and Post Session evaluations should be printed double sided and placed on individual tables. Learners should be instructed to complete the pre-session evaluation when they arrive. They should be asked to complete the post-session evaluation after the session is completed.
- Handouts: Handouts should be printed single sided and placed on the tables prior to the session. For this session, each table should receive only 1 of the 5 available quotes. Learners should be instructed that the will be directed when to utilize the handouts during the session.

*Instructor Overview:*

- Before Session Begins: Prior to the start of the session, the session leader should ask the learners to complete the pre-session assessment.
- Presentation: The session leader will read though the PowerPoint presentation specifically providing details about the case.
- Breakout Sessions: Each time the leader reaches a slide titled “In Your Small Groups” the leader will prompt the teams to discuss the questions on the slide and utilize a hand out if one is available for that section. During each breakout session allow 5-10 minutes for discussion as a small group and 3-5 minutes for groups to share their thoughts with the larger group. Ensure every group gets to report out at least once, but not necessarily for each breakout session. Time for each session should be geared towards the amount of time available to complete the module. For a 45 minute session, 10 minutes for discussion and 5 minutes for report out is sufficient. Following residents/learners reporting out when they think/feel about each discussion questions, the leader should continue with the slides to provide didactic and context for the questions addressed.
- Conclusion of Session: As the learners to complete the post-session assessment at the end of the session.

*Additional Session-Specific Instructions and Content Information:*

**“In Your Small Groups – 1”:** For this slide, ask the leaners to utilize the hand out which contains a quote at each table.

**Question 1, Slides 13 and 14:** Slides 13 and 14 contain evidence based information regarding risks and benefits of providing artificial nutrition and hydration at the end of life. This information should be provided by the session leader in reference to the 1^st^ question. Additional facts include:

- Arguments against artificial nutrition/hydration:
  - Comatose patients don’t experience hunger
  - Stopping hydration/nutrition can improvement of symptoms related to fluid overload, including a decreased need for foley or straight catheters
  - Avoids IVs or NGs
- Arguments for artificial nutrition/hydration:
  - Some families equate stopping hydration/nutrition as killing their child;
  - Some families feel they need to continue hydration/nutrition to be a good parent;
  - Belief that hydration/nutrition is a basic human need they cannot stop;
  - Dehydration symptoms may be better with fluids
  - Medications may accumulate if kidneys start to fail without hydration

**Question 2:** There are no “right” or “wrong” answers to how residents feel when they read the quotes. This is a space to allow residents to reflect and process experiences they have had. Having a senior resident and/or attending at the table is helpful because they are more likely to have experienced the scenario of withdrawing/withholding artificial nutrition or hydration. There are also no “perfect” responses to these. Here are some examples of ways to address potential anticipated concerns.

- Quote on Slide 8: This parent is expressing the sentiment that her child has an extremely poor quality of life necessitating multiple medications to provide even some relief of suffering. In these situations, families may view stopping artificial nutrition/hydration as a way to allow the patient to die naturally instead of keeping the child alive just to suffer. This is often a difficult conversation to have, but could be a conversation a provider could initiate if a family is expressing concerns of excessive suffering. Palliative care involvement (and potentially ethics) in this discussion would be helpful.
- Quote on Slide 9: This parent is expressing frustration with multiple opinions, which is OK in medicine, but can lead to confusion and second guessing when it comes to difficult decisions at the end of life. A provider meeting and/or ethics consult could be helpful to help providers get on the same page regarding treatments that can be offered. Once the family makes a decision, it is important to support them.
- Quote on Slide 10: This parent is expressing concern that what she was choosing is illegal. In the situation of a dying patient, it is important to be able to reassure the family that withdrawing artificial nutrition/hydration is not the same as euthanasia or physician assisted suicide. Also, if the family’s does not want to not prolong the dying process, it may be something the doctor could recommend.
- Quote on Slide 11: This parent is expressing concern that her friends and family would not understand the decision she made and actually equate it to murder. It is important that as a physician you recognize families may feel isolated when making this decision. Providing support and reassurance that the family is making a good and loving decision is important. Additionally, being available to discuss the decision with those other family members may be helpful for some families. After a loss of a child, if a family now feels isolated from their support system it may make the grieving process even more difficult.
- Quote on Slide 12: These parents express 2 different experiences of dying after discontinuation of artificial nutrition and hydration. It is very difficult to know what the dying process will look like in advance and physicians are terrible at prognostication. Depending on how much fluid a child is receiving with meds, the length of time it may take for someone to die is variable. Having a palliative care or hospice provider that can answer any questions and provide support if the dying process is not going as anticipated can be helpful.

**“In Your Small Groups – 2”:** The session leader should prompt the residents to break into small groups to discuss.

**Question 1:** Slides 19 and 20 provide didactic.

**Question 2:** Slide 21 provides didactic. Additional content includes:

- The American Academy of Pediatrics concludes in its policy that the withdrawal of medically administered fluids and nutrition for pediatric patients is ethically acceptable in limited circumstances.
- Example clinical scenarios where the burdens of MPNH might outweigh to benefits.
  - Children in a perm vegetative state or anencephalic infants might have such a diminished level of consciousness that they may not obtain benefit from any medical interventions
  - For children with weeks or less to live, MPNH will only prolong the dying process.
  - For children needing long-term total parenteral nutrition, particularly in the absence of any bowel function, is associated with a high rate of complications
  - Parents almost always have the right to refuse to pursue organ transplant if they don’t think it is in their child’s best interest. If the parents are refusing an organ transplant TPN may only prolong the dying process

*References:*

The session leader can utilize the references listed to obtain additional content expertise if needed prior to leading the session.
