# Supplementary material for: Ethical Dilemmas at the Beginning and End of Life: A Needs-Based, Experience-Informed, Small-Group, Case-Based Curriculum for Pediatric Residents
Source: MedEdPORTAL. 2020 Apr 3;16:10895. doi: 10.15766/mep_2374-8265.10895 (PMC7187913; doi:10.15766/mep_2374-8265.10895)
Supplement: Supplementary file 1 — Medically Provided Fluids Nutrition PowerPoint.pptxMedically Provided Fluids Nutrition Instructor Guide.docxMedically Provided Fluids Nutrition Handout.docxMedically Provided Fluids Nutrition Assessment Questions.docxFutility and Goals of Care PowerPoint.pptxFutility and Goals of Care Instructor Guide.docxFutility and Goals of Care Handout.docxFutility and Goals of Care Assessment Questions.docxEthical Issues in Neonatology PowerPoint.pptxEthical Issues in Neonatology Instructor Guide.docxEthical Issues in Neonatology Assessment Questions.docx [file mep-16-10895-s001.zip › C. Medically Provided Fluids Nutrition Handout.docx]

**Parents’ perceptions of the child’s poor quality of life**

- “[H]e went home on oxygen, he was seizing constantly. I just said, ‘that’s it, I can’t take this anymore. I can’t watch him’. He would break out in this sweat for hours and he was given a lot of medication to control it and the only way that it could really be controlled was with morphine, so, what kind of a life was he having at that point?” (Marilyn)

**Consistent message from professionals**

- “I think what made it hard . . . was this teeter/totter opinion . . . am I really making the right choice then? . . .Because I think if everybody said ‘this is what you do’, it’s easier to accept. . . . I think it’s best that the health care people get together, come to an agreement first, and then present it to the family” (Lina)

**Physician presentation of the option to forgo medically provided fluids and nutrition**

- “[Palliative physician] had [written] a paper, which he had given to myself and [husband] . . it just made me feel like ‘so it is OK, it’s OK to have these feelings and it’s OK to let him go that way. It’s not, it’s not wrong. That it’s OK to let him go.’ . . . And, he was hesitant because I think most of them don’t want to say, right out, that this is what you should do. It’s more like ‘did you know that this is an option?’ . . . [he] gave us that report and it was OK to do this, it wasn’t illegal.” (Marilyn)

**Concerns about being judged**

- “I thought that a lot of people would think it was disgusting and horrible and murderous if we ever said that we were going to take away the food. So I never discussed it with anyone outside of [husband] and our doctors and [friend] who’s a doctor, because I just felt that people would think that it was, you know, paramount to murder.” (Jessica)

**Managing expectations**

- “He became more comfortable. . . . he wasn’t bloated . . . when he was being fed, like his belly would just sort of bloat. And he wasn’t breaking down the food that he was receiving, so it was very uncomfortable for him. So once we stopped [ANH] [it] just sort of allowed him to go peacefully.” (Coaster)
- “We just saw him starting to waste away and that was the hard part.” (Jack)
