# Supplementary material for: Ethical Dilemmas at the Beginning and End of Life: A Needs-Based, Experience-Informed, Small-Group, Case-Based Curriculum for Pediatric Residents
Source: MedEdPORTAL. 2020 Apr 3;16:10895. doi: 10.15766/mep_2374-8265.10895 (PMC7187913; doi:10.15766/mep_2374-8265.10895)
Supplement: Supplementary file 1 — Medically Provided Fluids Nutrition PowerPoint.pptxMedically Provided Fluids Nutrition Instructor Guide.docxMedically Provided Fluids Nutrition Handout.docxMedically Provided Fluids Nutrition Assessment Questions.docxFutility and Goals of Care PowerPoint.pptxFutility and Goals of Care Instructor Guide.docxFutility and Goals of Care Handout.docxFutility and Goals of Care Assessment Questions.docxEthical Issues in Neonatology PowerPoint.pptxEthical Issues in Neonatology Instructor Guide.docxEthical Issues in Neonatology Assessment Questions.docx [file mep-16-10895-s001.zip › D. Medically Provided Fluids Nutrition Assessment Questions.docx]

End of Life and Ethics Curriculum Evaluation
Medically Provided Fluids and Nutrition

**Level of Training:**

M3 AI/M4 PGY1 PGY2 PGY3 PGY4 PGY5 Other _________________________

**Pre-Session Evaluation**

I feel comfortable describing the benefits and burdens of utilizing medically provided nutrition or hydration at the end of life

1 = strongly disagree 2 = disagree 3 = neither 4 = agree 5 = strongly agree

I feel comfortable counseling families on the decision to withdraw or withhold medically provided nutrition at the end of life.

1 = strongly disagree 2 = disagree 3 = neither 4 = agree 5 = strongly agree

I understand the ethical considerations involved in the decision to withdraw or withhold medically provided hydration and nutrition.

1 = strongly disagree 2 = disagree 3 = neither 4 = agree 5 = strongly agree

I understand the distinction between medical interventions and basic patient care.

1 = strongly disagree 2 = disagree 3 = neither 4 = agree 5 = strongly agree

End of Life and Ethics Curriculum Evaluation
Medically Provided Fluids and Nutrition

**Post-Session Evaluation**

I feel comfortable describing the benefits and burdens of utilizing medically provided nutrition or hydration at the end of life

1 = strongly disagree 2 = disagree 3 = neither 4 = agree 5 = strongly agree

I feel comfortable counseling families on the decision to withdraw or withhold medically provided nutrition at the end of life.

1 = strongly disagree 2 = disagree 3 = neither 4 = agree 5 = strongly agree

I understand the ethical considerations involved in the decision to withdraw or withhold medically provided hydration and nutrition.

1 = strongly disagree 2 = disagree 3 = neither 4 = agree 5 = strongly agree

I understand the distinction between medical interventions and basic patient care.

1 = strongly disagree 2 = disagree 3 = neither 4 = agree 5 = strongly agree

What was most effective about this session?

What are areas for improvement of this session?
