# Supplementary material for: Ethical Dilemmas at the Beginning and End of Life: A Needs-Based, Experience-Informed, Small-Group, Case-Based Curriculum for Pediatric Residents
Source: MedEdPORTAL. 2020 Apr 3;16:10895. doi: 10.15766/mep_2374-8265.10895 (PMC7187913; doi:10.15766/mep_2374-8265.10895)
Supplement: Supplementary file 1 — Medically Provided Fluids Nutrition PowerPoint.pptxMedically Provided Fluids Nutrition Instructor Guide.docxMedically Provided Fluids Nutrition Handout.docxMedically Provided Fluids Nutrition Assessment Questions.docxFutility and Goals of Care PowerPoint.pptxFutility and Goals of Care Instructor Guide.docxFutility and Goals of Care Handout.docxFutility and Goals of Care Assessment Questions.docxEthical Issues in Neonatology PowerPoint.pptxEthical Issues in Neonatology Instructor Guide.docxEthical Issues in Neonatology Assessment Questions.docx [file mep-16-10895-s001.zip › E. Futility and Goals of Care PowerPoint.pptx]

## Slide 1
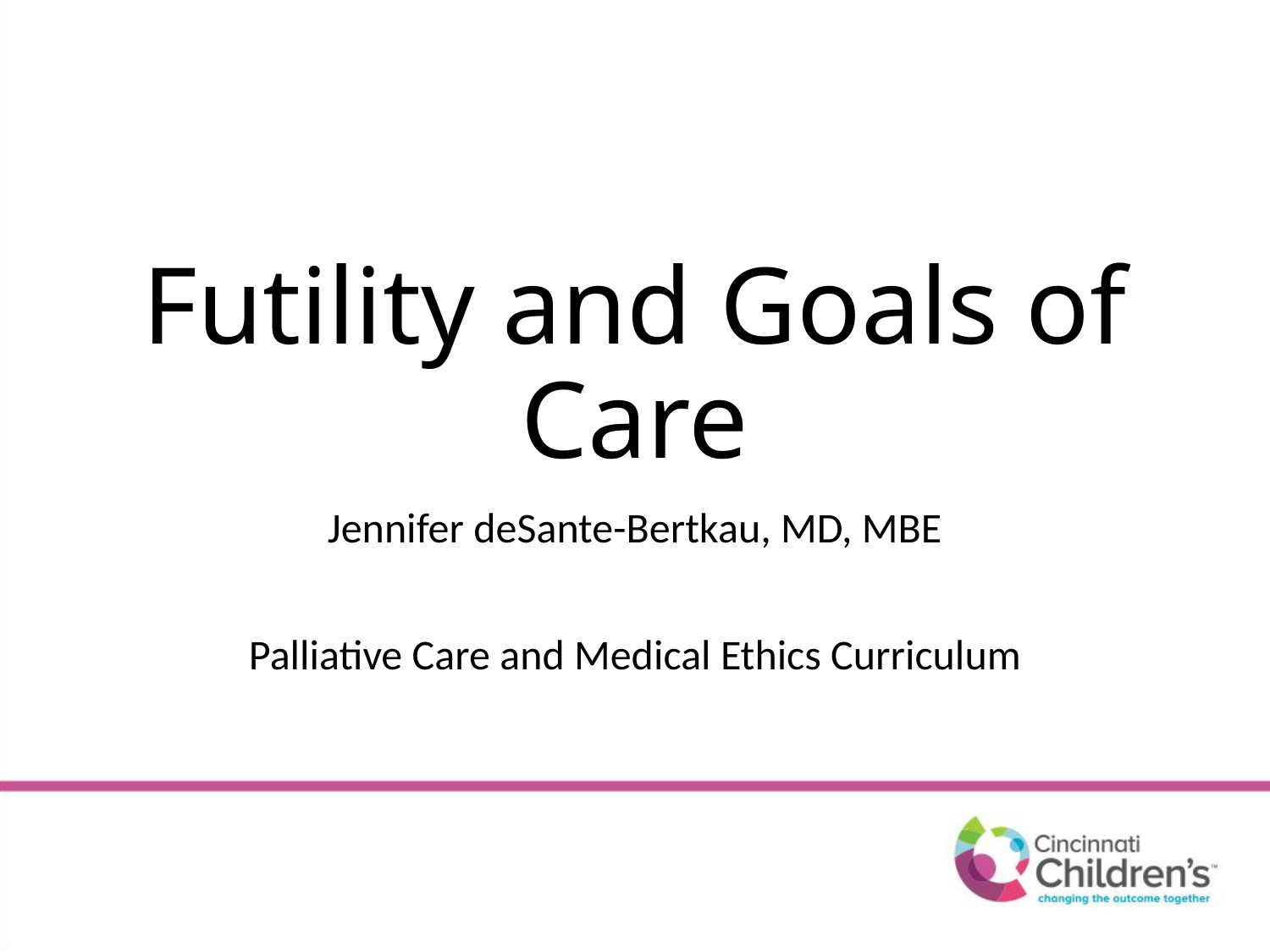

# Futility and Goals of Care
Jennifer deSante-Bertkau, MD, MBE
Palliative Care and Medical Ethics Curriculum

## Slide 2
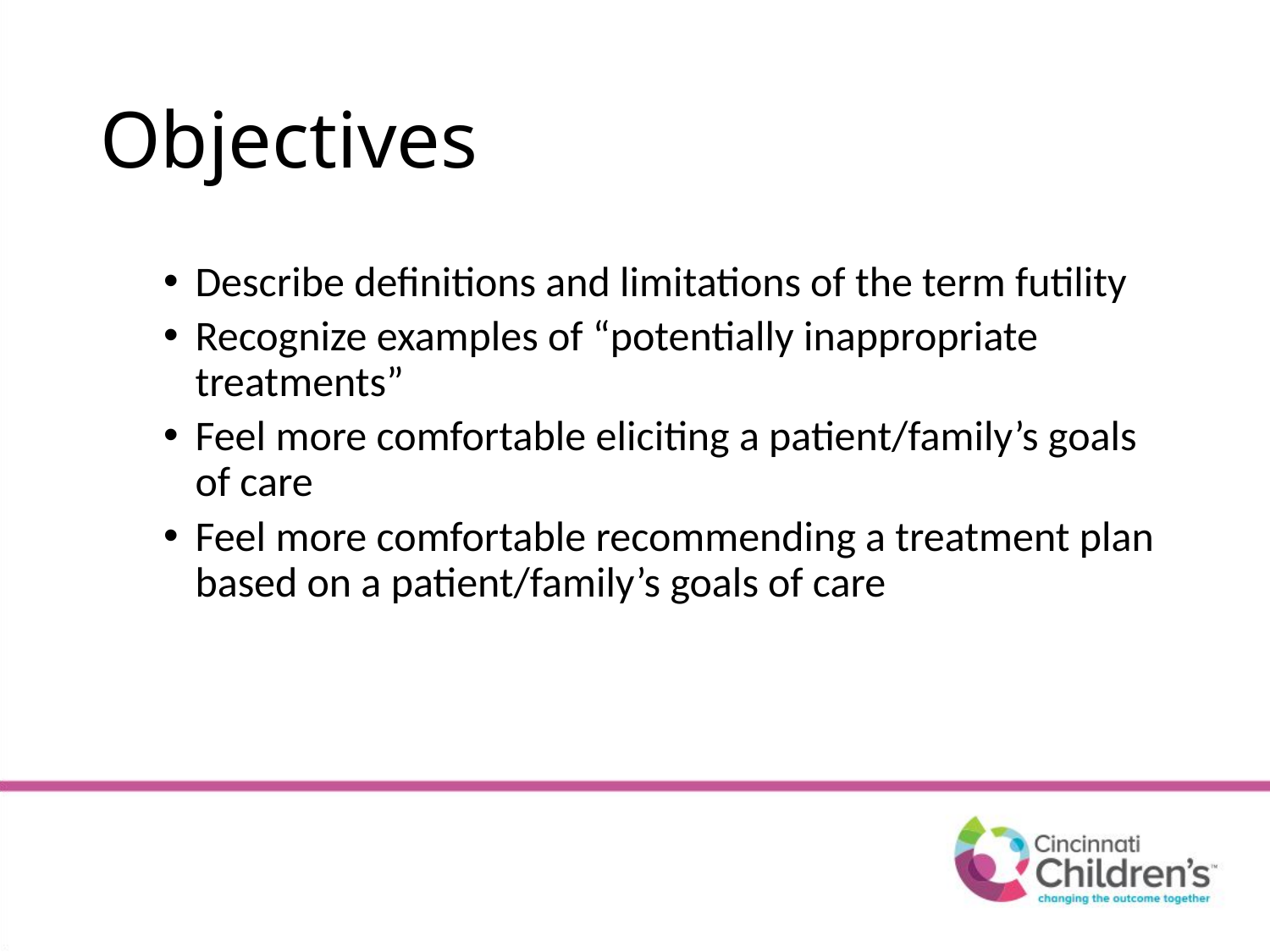

# Objectives
Describe definitions and limitations of the term futility
Recognize examples of “potentially inappropriate treatments”
Feel more comfortable eliciting a patient/family’s goals of care
Feel more comfortable recommending a treatment plan based on a patient/family’s goals of care

## Slide 3
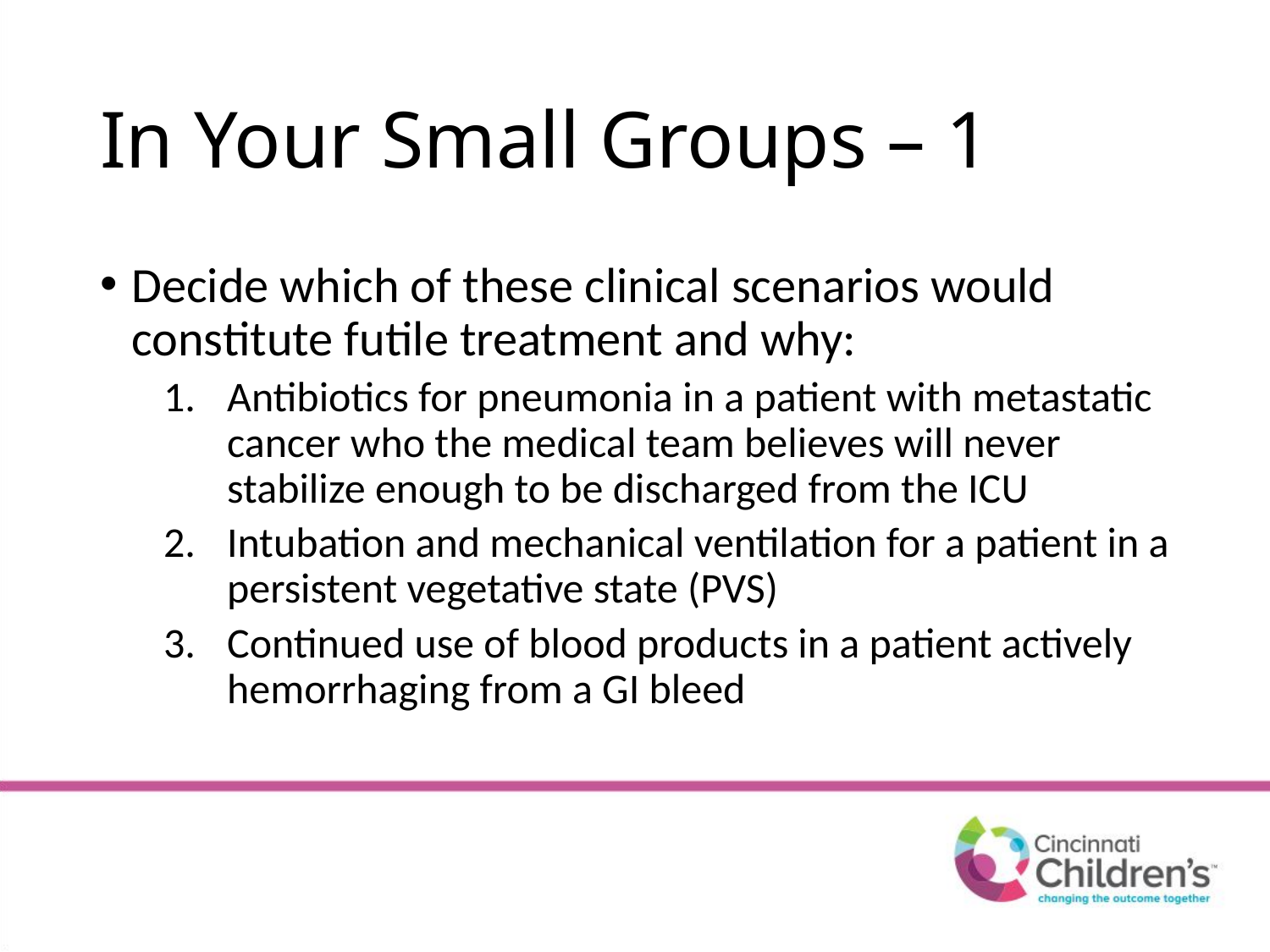

# In Your Small Groups – 1
Decide which of these clinical scenarios would constitute futile treatment and why:
Antibiotics for pneumonia in a patient with metastatic cancer who the medical team believes will never stabilize enough to be discharged from the ICU
Intubation and mechanical ventilation for a patient in a persistent vegetative state (PVS)
Continued use of blood products in a patient actively hemorrhaging from a GI bleed

## Slide 4
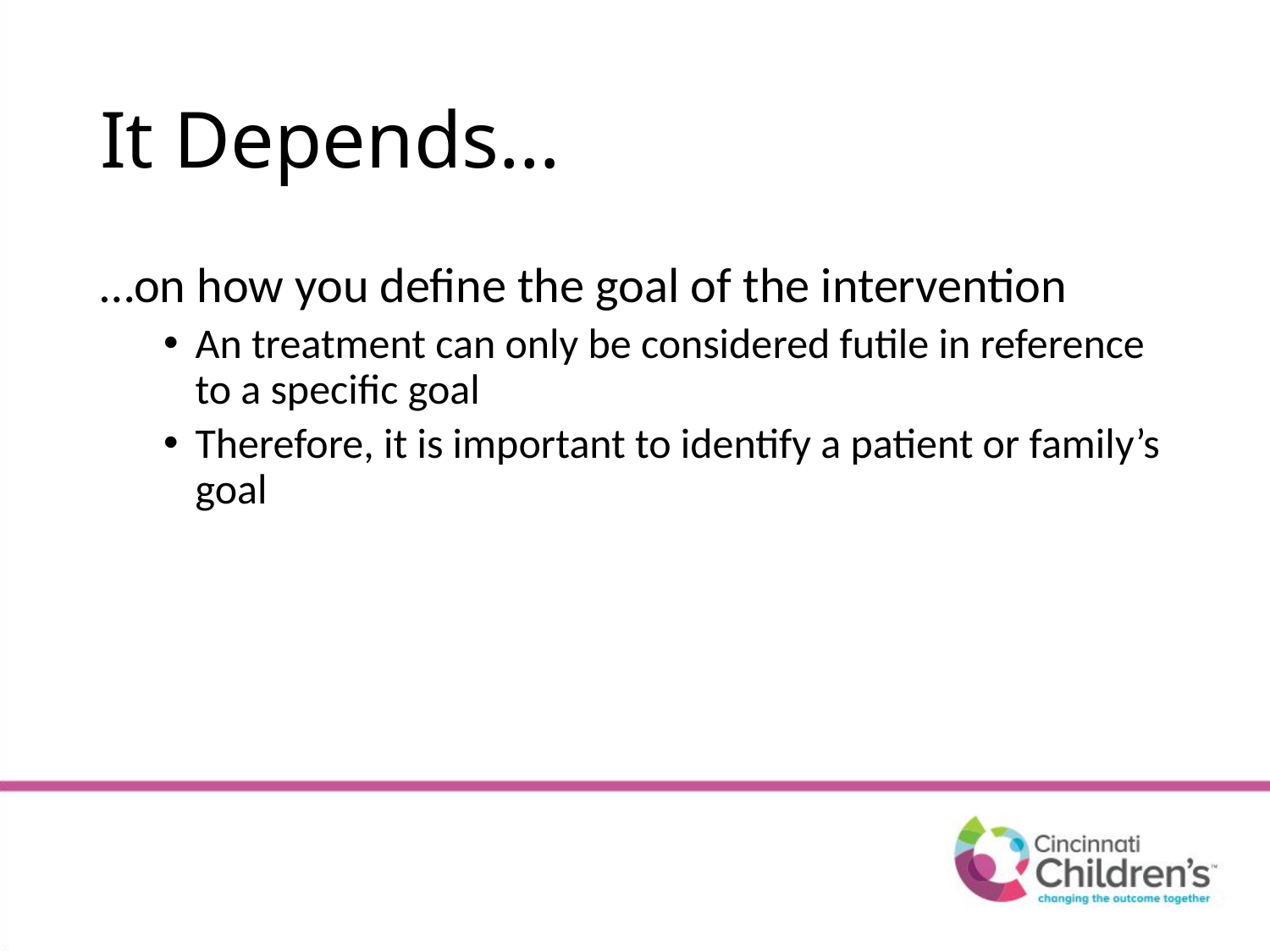

# It Depends…
…on how you define the goal of the intervention
An treatment can only be considered futile in reference to a specific goal
Therefore, it is important to identify a patient or family’s goal

## Slide 5
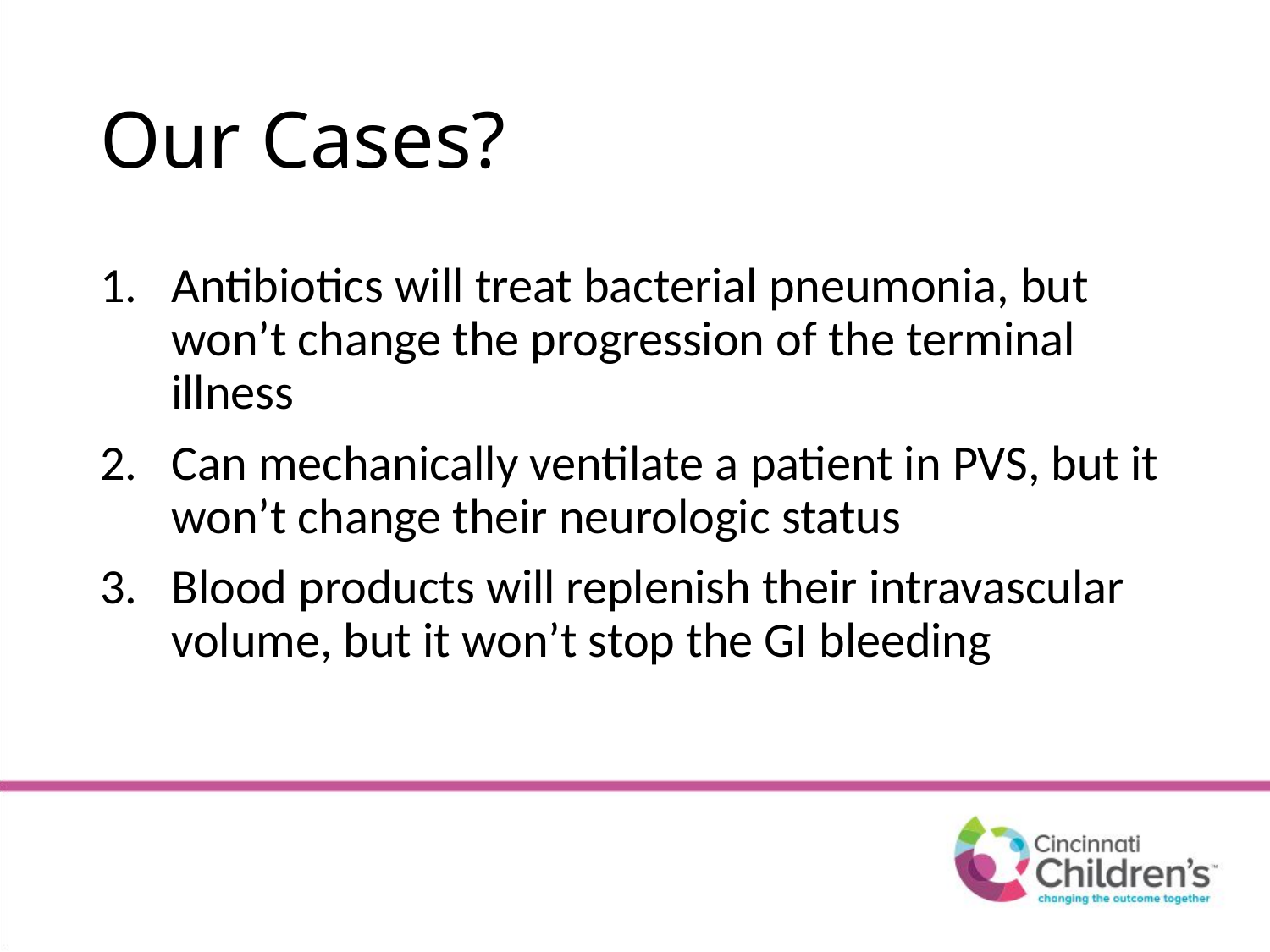

# Our Cases?
Antibiotics will treat bacterial pneumonia, but won’t change the progression of the terminal illness
Can mechanically ventilate a patient in PVS, but it won’t change their neurologic status
Blood products will replenish their intravascular volume, but it won’t stop the GI bleeding

## Slide 6
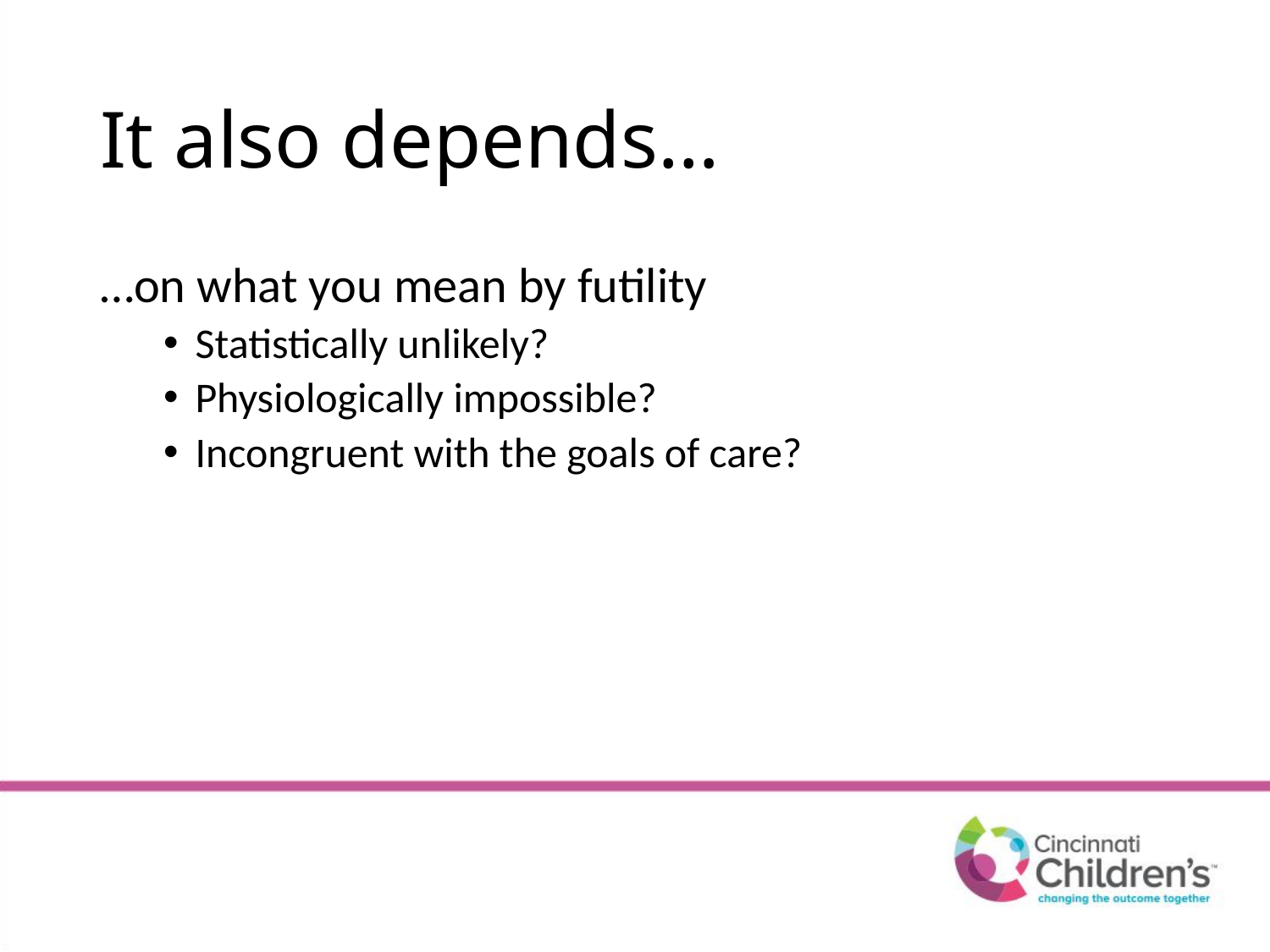

# It also depends…
…on what you mean by futility
Statistically unlikely?
Physiologically impossible?
Incongruent with the goals of care?

## Slide 7
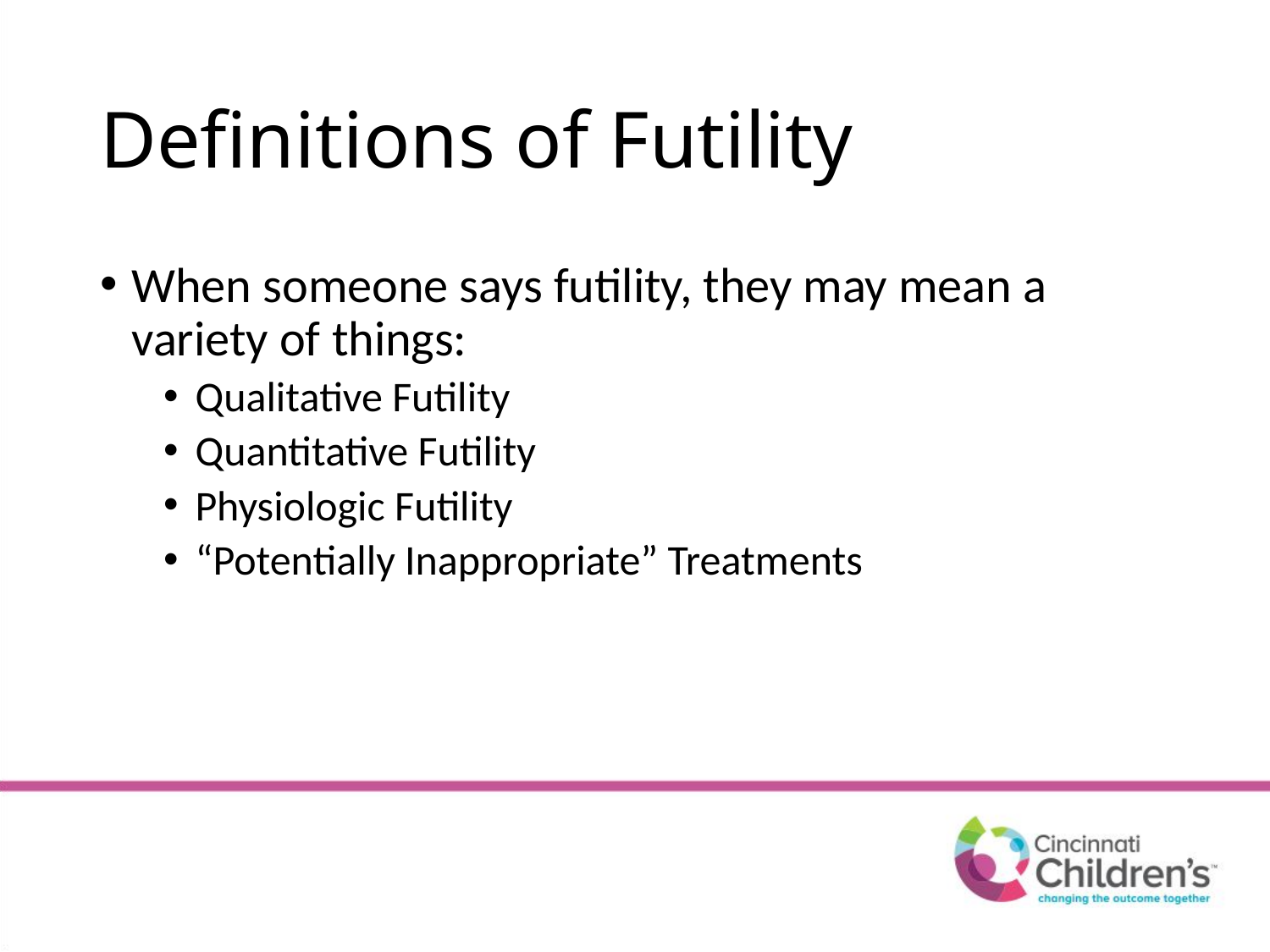

# Definitions of Futility
When someone says futility, they may mean a variety of things:
Qualitative Futility
Quantitative Futility
Physiologic Futility
“Potentially Inappropriate” Treatments

## Slide 8
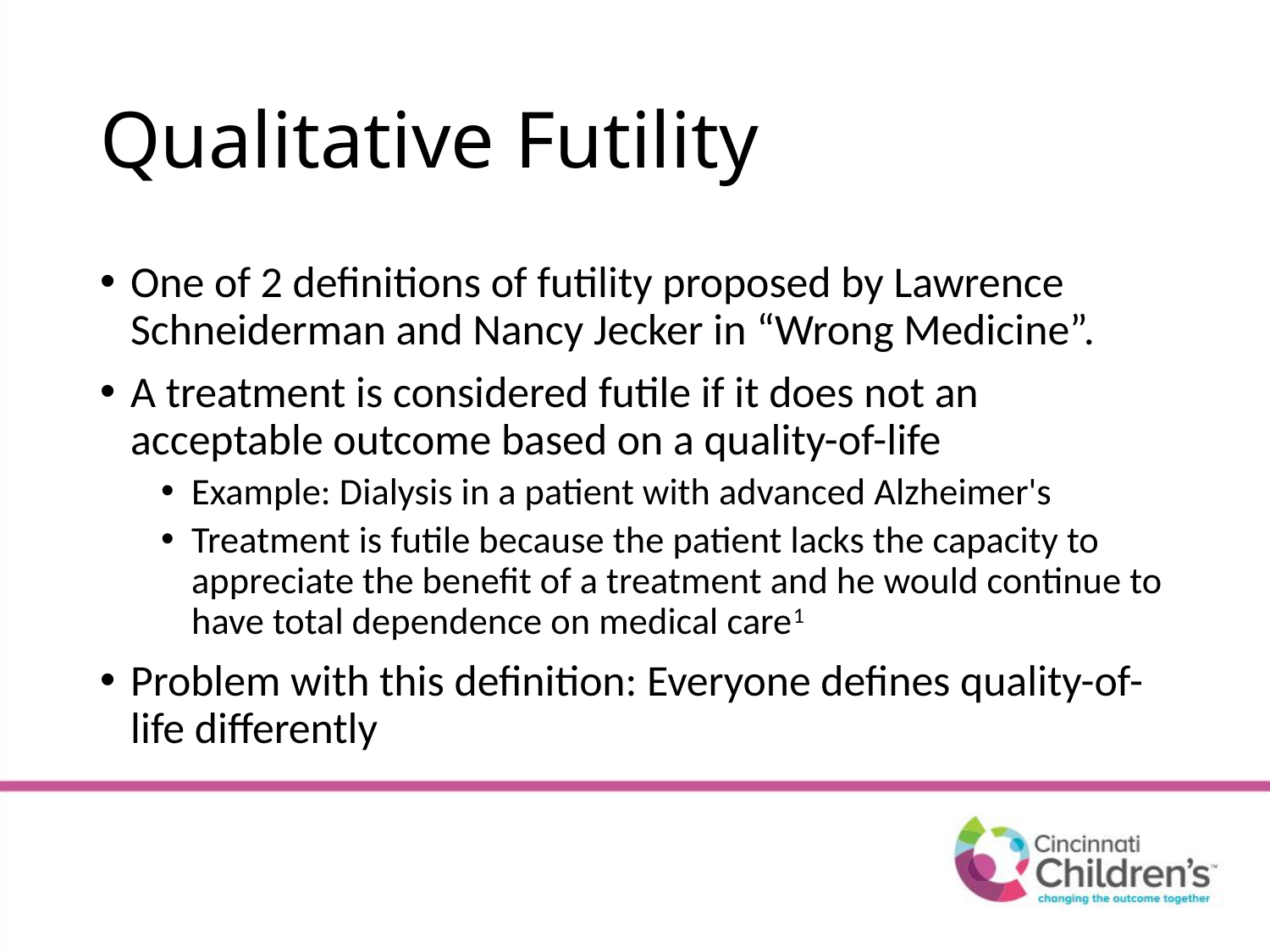

# Qualitative Futility
One of 2 definitions of futility proposed by Lawrence Schneiderman and Nancy Jecker in “Wrong Medicine”.
A treatment is considered futile if it does not an acceptable outcome based on a quality-of-life
Example: Dialysis in a patient with advanced Alzheimer's
Treatment is futile because the patient lacks the capacity to appreciate the benefit of a treatment and he would continue to have total dependence on medical care1
Problem with this definition: Everyone defines quality-of-life differently

## Slide 9
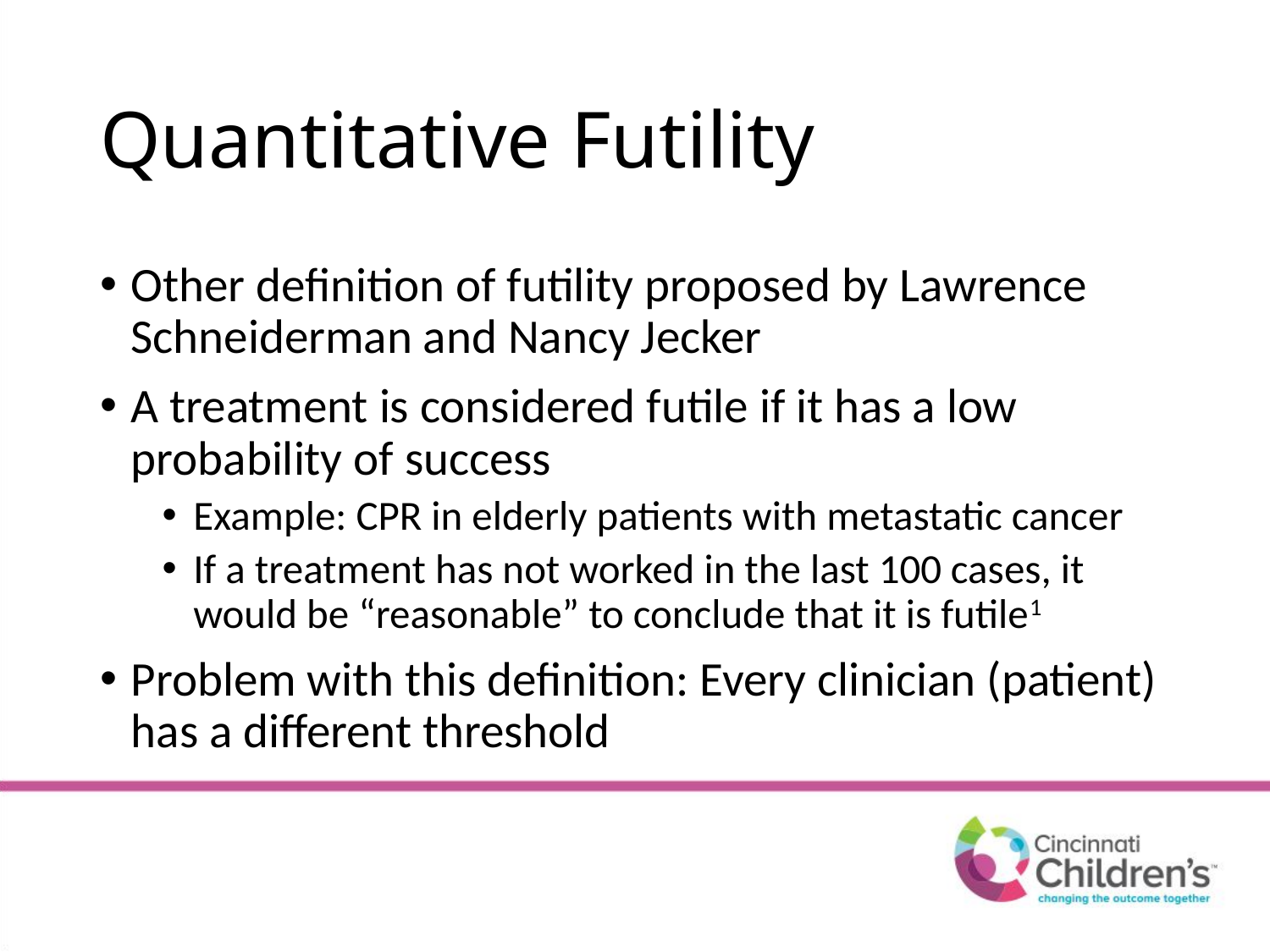

# Quantitative Futility
Other definition of futility proposed by Lawrence Schneiderman and Nancy Jecker
A treatment is considered futile if it has a low probability of success
Example: CPR in elderly patients with metastatic cancer
If a treatment has not worked in the last 100 cases, it would be “reasonable” to conclude that it is futile1
Problem with this definition: Every clinician (patient) has a different threshold

## Slide 10
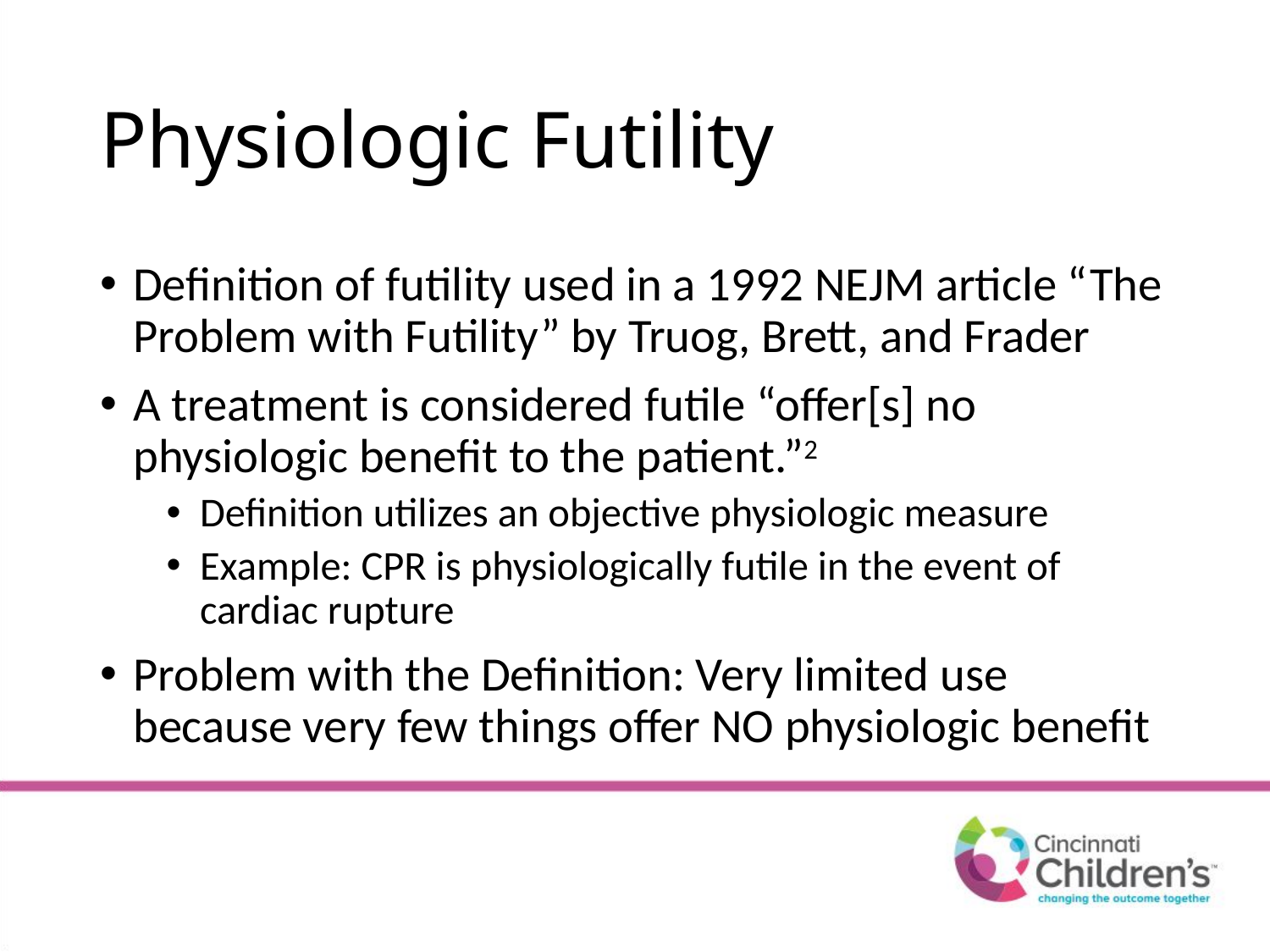

# Physiologic Futility
Definition of futility used in a 1992 NEJM article “The Problem with Futility” by Truog, Brett, and Frader
A treatment is considered futile “offer[s] no physiologic benefit to the patient.”2
Definition utilizes an objective physiologic measure
Example: CPR is physiologically futile in the event of cardiac rupture
Problem with the Definition: Very limited use because very few things offer NO physiologic benefit

## Slide 11
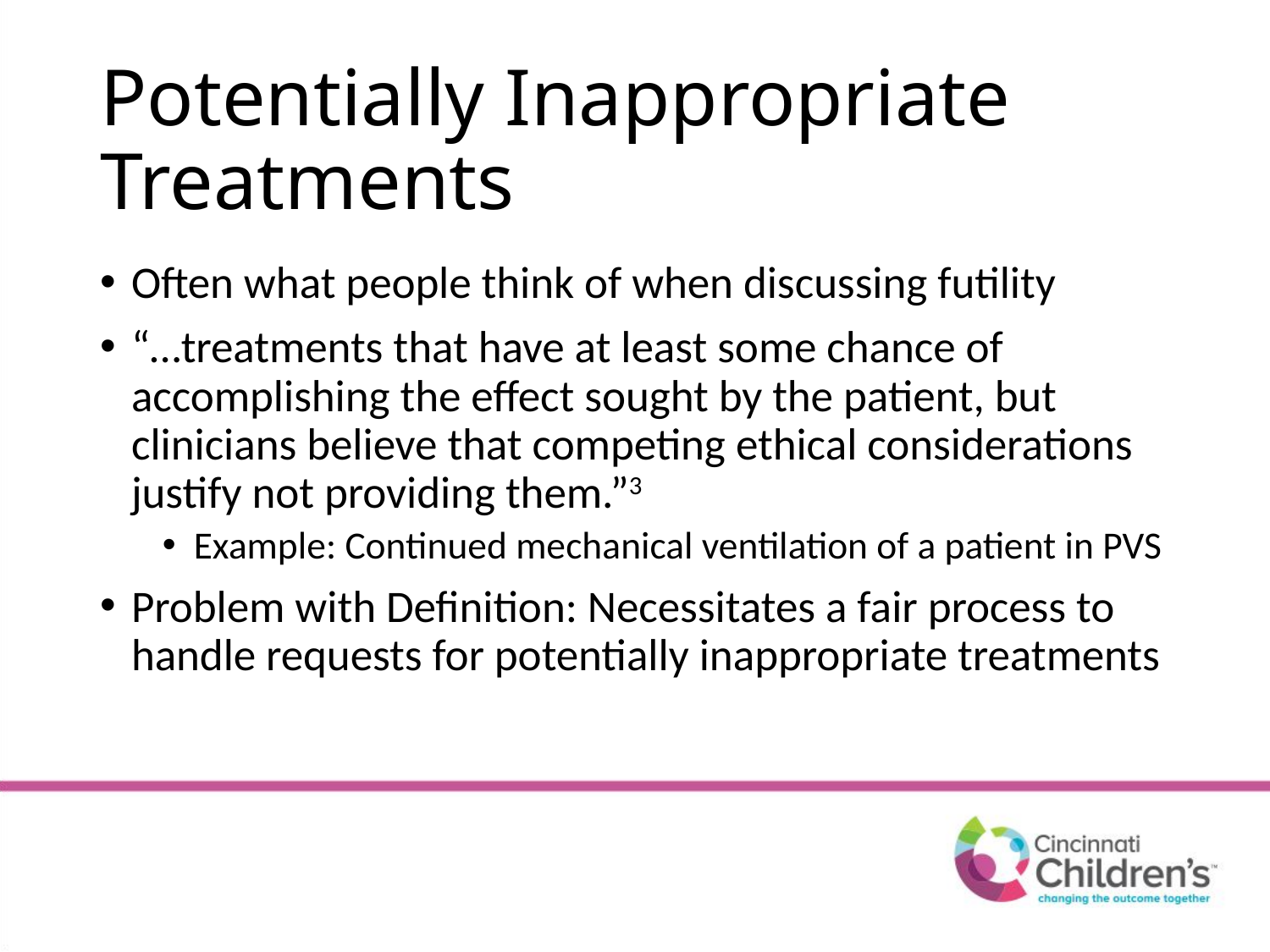

# Potentially Inappropriate Treatments
Often what people think of when discussing futility
“…treatments that have at least some chance of accomplishing the effect sought by the patient, but clinicians believe that competing ethical considerations justify not providing them.”3
Example: Continued mechanical ventilation of a patient in PVS
Problem with Definition: Necessitates a fair process to handle requests for potentially inappropriate treatments

## Slide 12
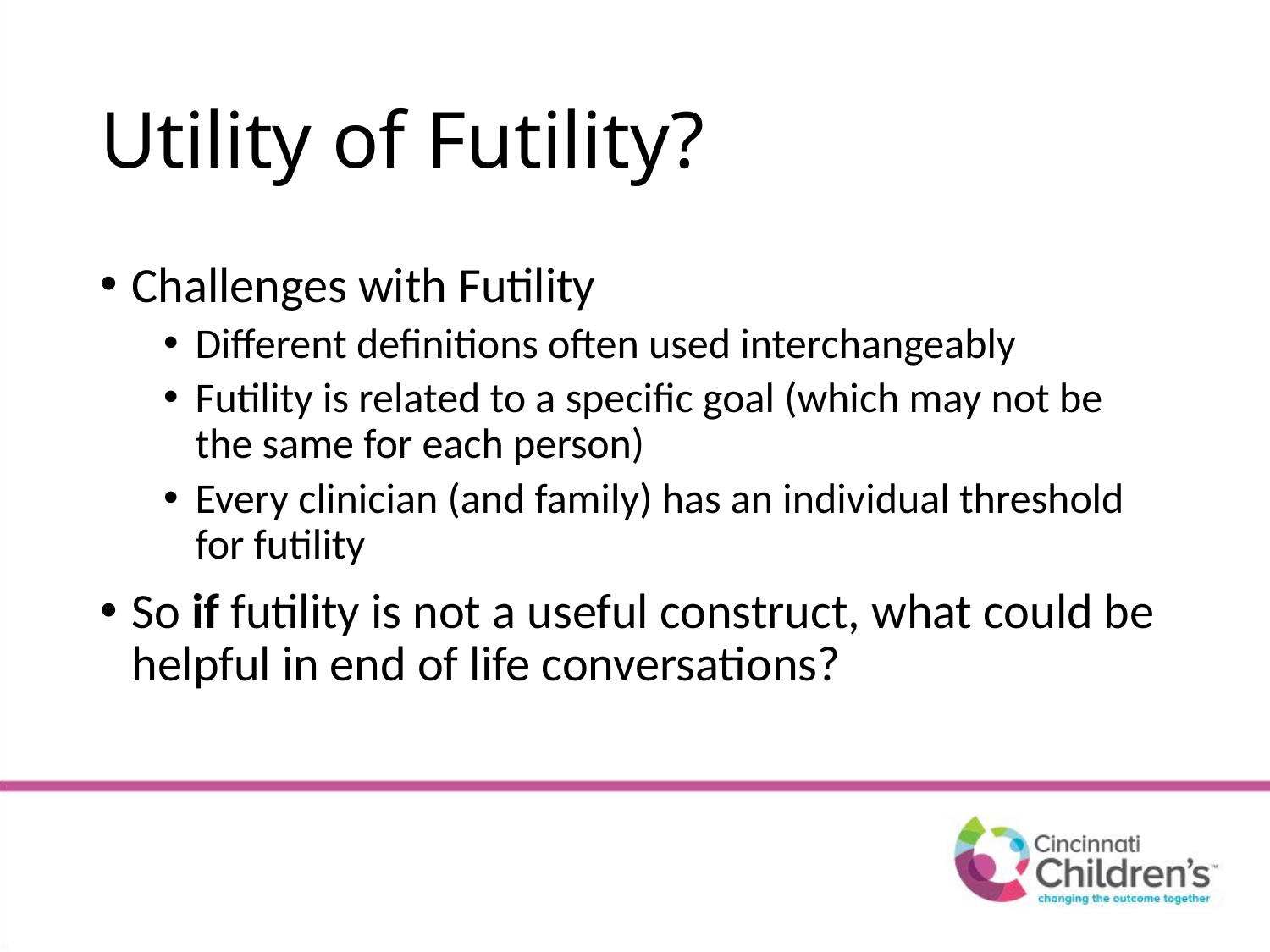

# Utility of Futility?
Challenges with Futility
Different definitions often used interchangeably
Futility is related to a specific goal (which may not be the same for each person)
Every clinician (and family) has an individual threshold for futility
So if futility is not a useful construct, what could be helpful in end of life conversations?

## Slide 13
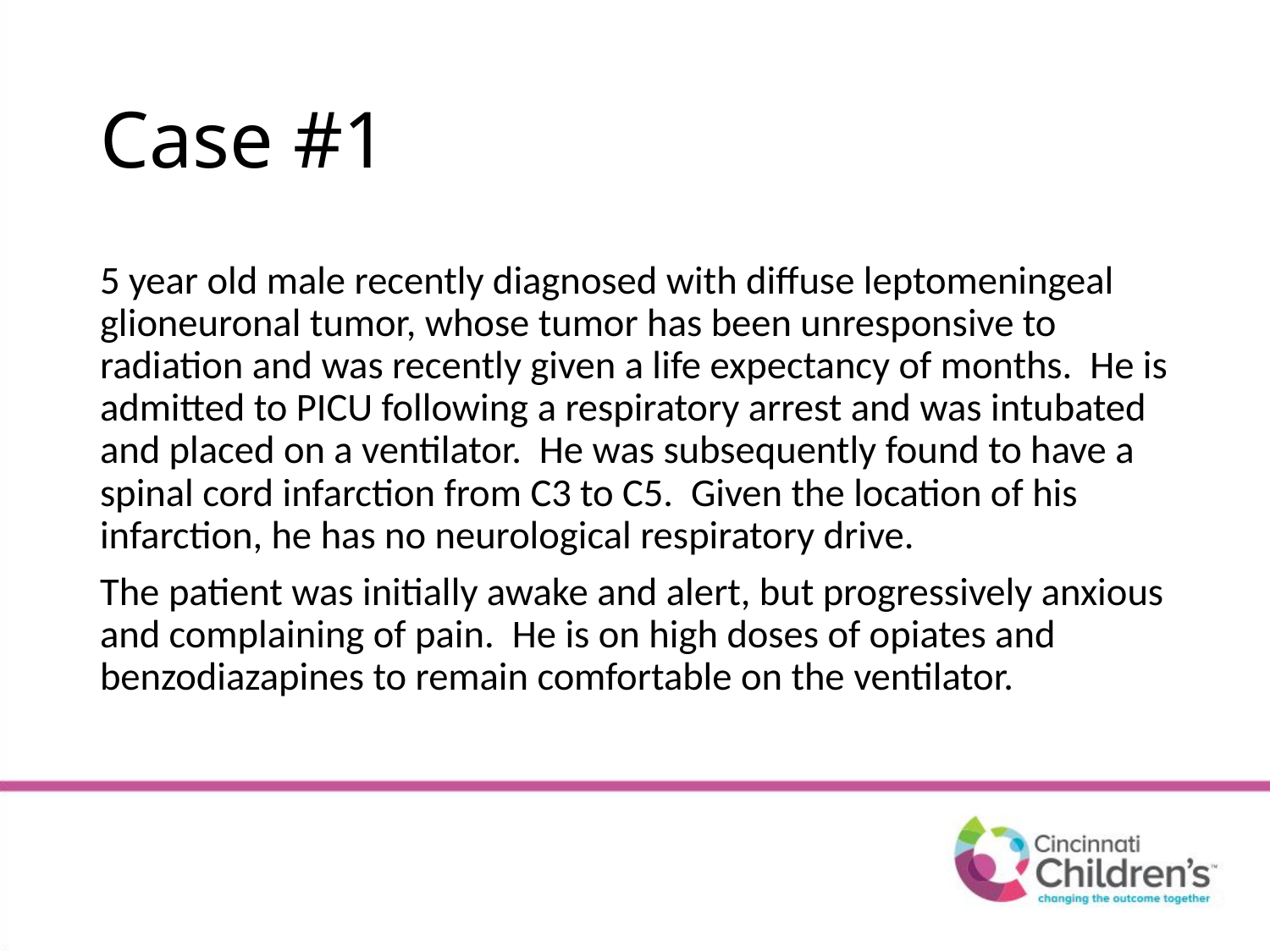

# Case #1
5 year old male recently diagnosed with diffuse leptomeningeal glioneuronal tumor, whose tumor has been unresponsive to radiation and was recently given a life expectancy of months. He is admitted to PICU following a respiratory arrest and was intubated and placed on a ventilator. He was subsequently found to have a spinal cord infarction from C3 to C5. Given the location of his infarction, he has no neurological respiratory drive.
The patient was initially awake and alert, but progressively anxious and complaining of pain. He is on high doses of opiates and benzodiazapines to remain comfortable on the ventilator.

## Slide 14
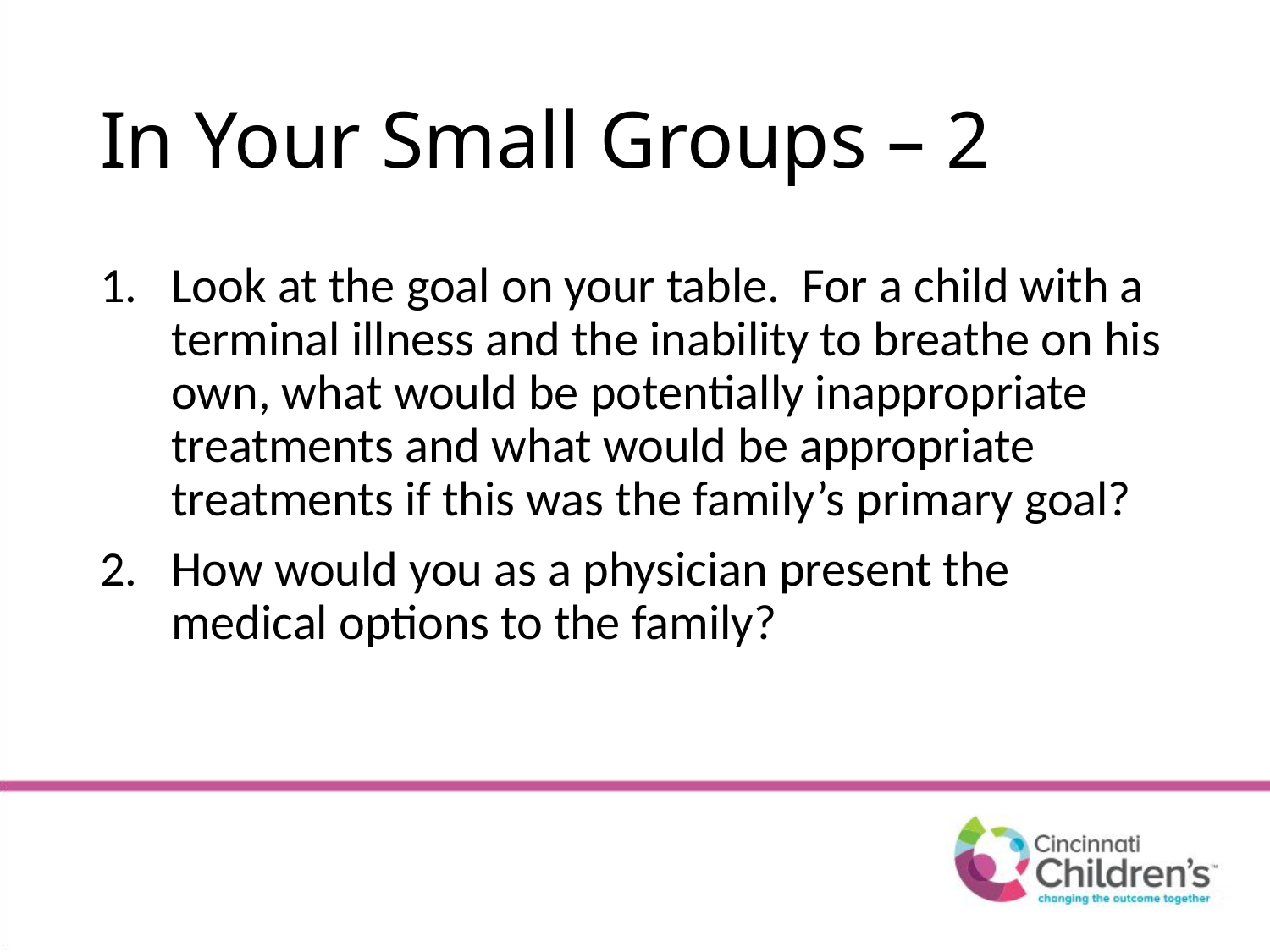

# In Your Small Groups – 2
Look at the goal on your table. For a child with a terminal illness and the inability to breathe on his own, what would be potentially inappropriate treatments and what would be appropriate treatments if this was the family’s primary goal?
How would you as a physician present the medical options to the family?

## Slide 15
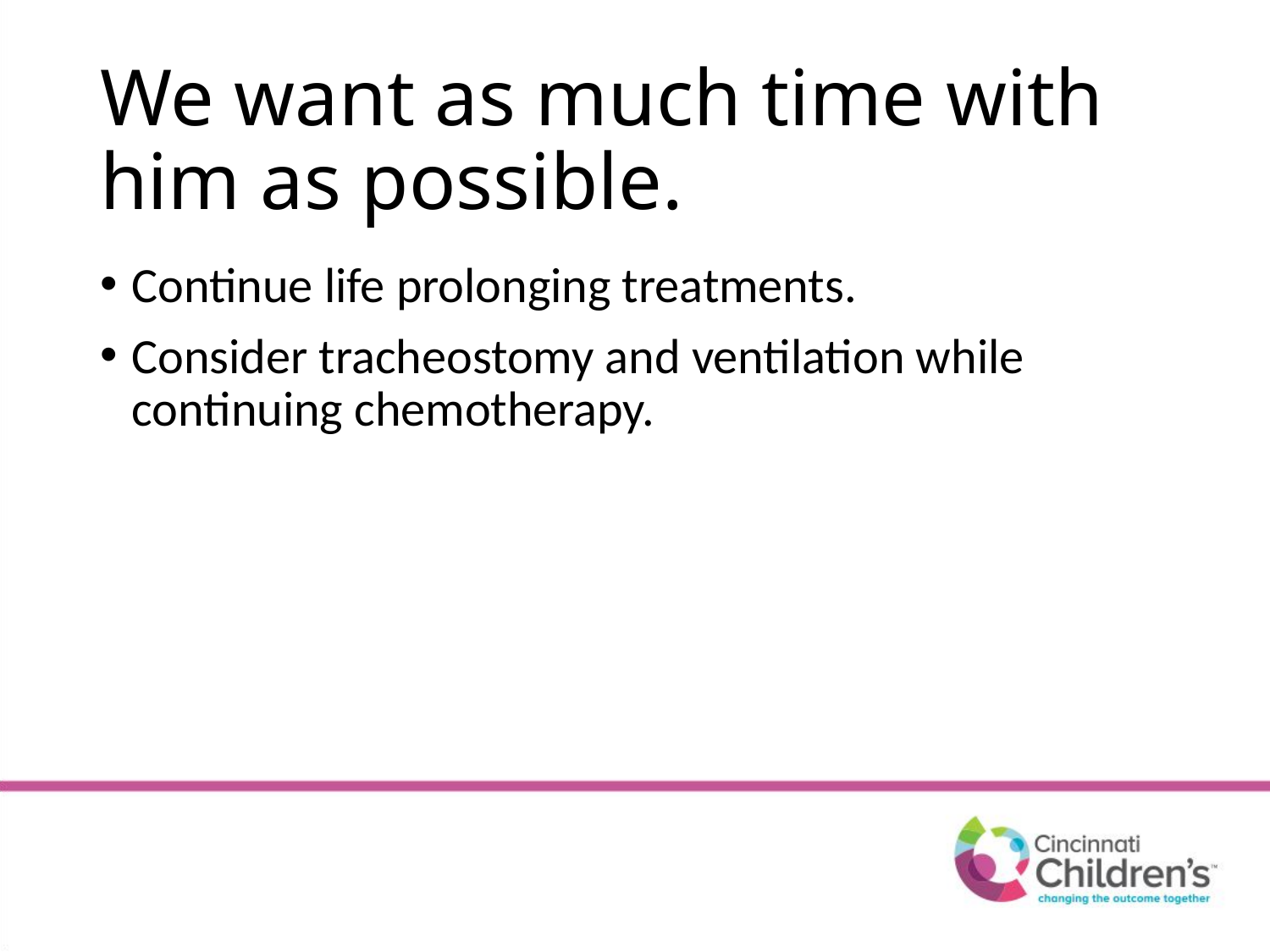

# We want as much time with him as possible.
Continue life prolonging treatments.
Consider tracheostomy and ventilation while continuing chemotherapy.

## Slide 16
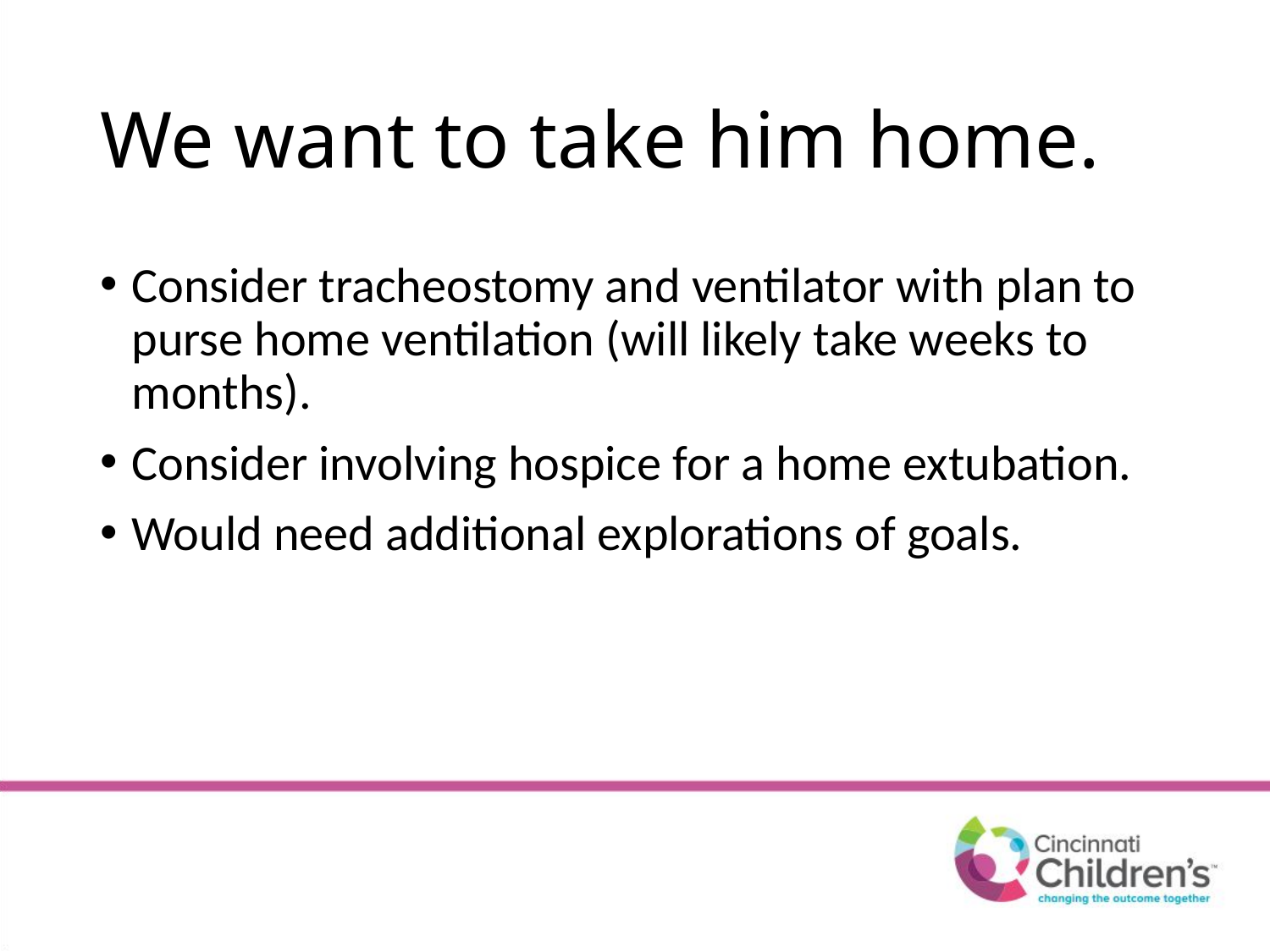

# We want to take him home.
Consider tracheostomy and ventilator with plan to purse home ventilation (will likely take weeks to months).
Consider involving hospice for a home extubation.
Would need additional explorations of goals.

## Slide 17
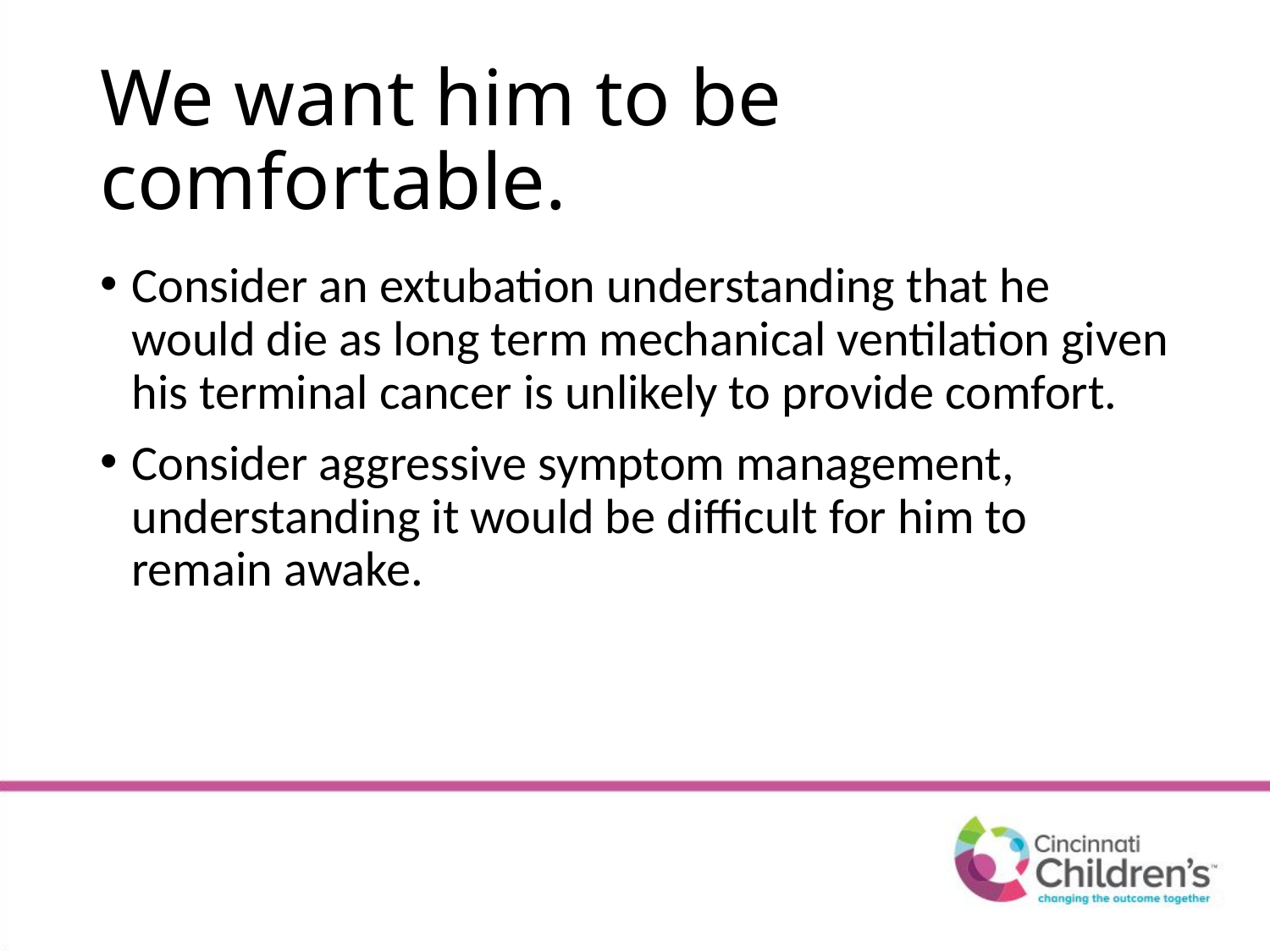

# We want him to be comfortable.
Consider an extubation understanding that he would die as long term mechanical ventilation given his terminal cancer is unlikely to provide comfort.
Consider aggressive symptom management, understanding it would be difficult for him to remain awake.

## Slide 18
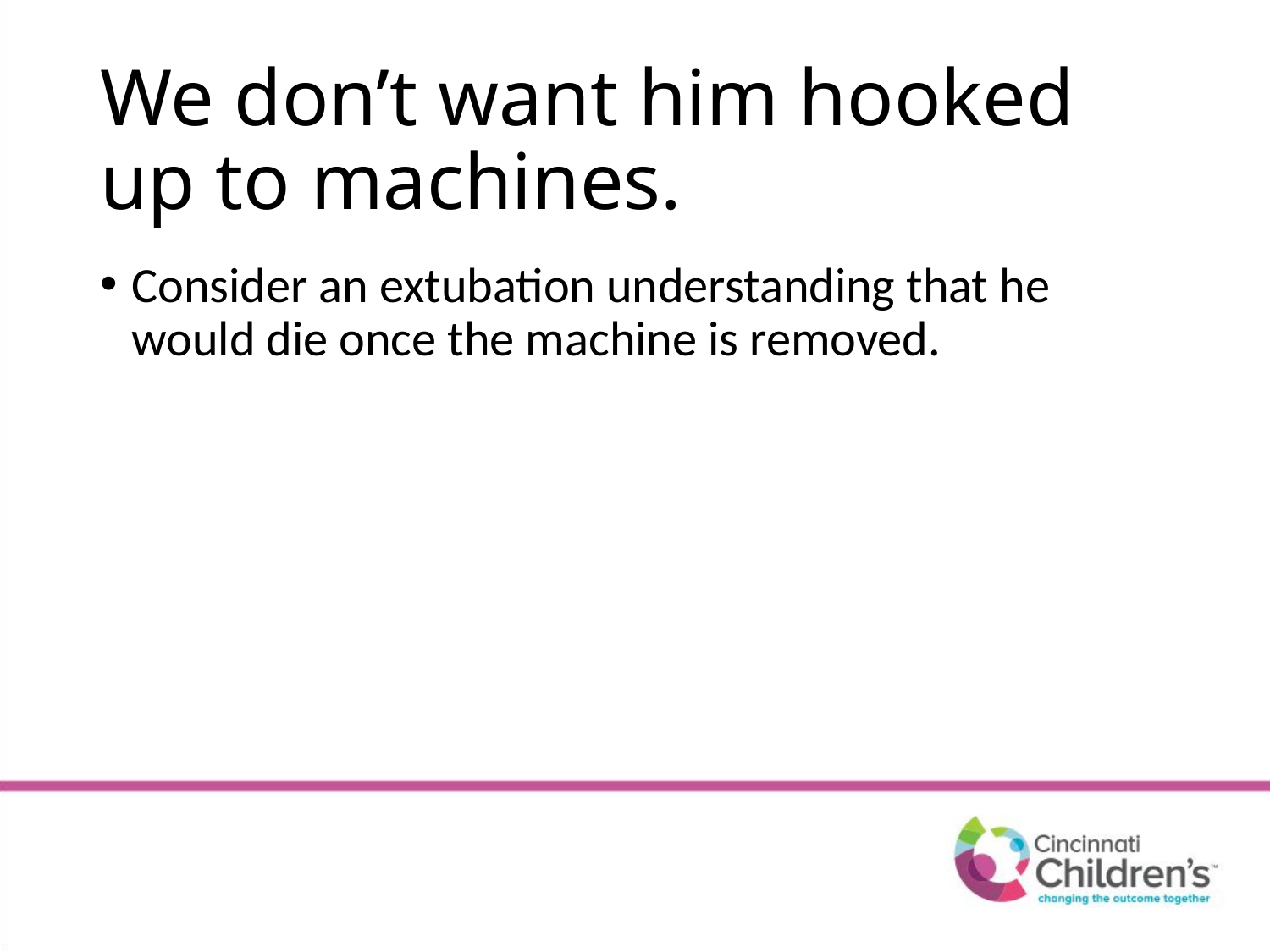

# We don’t want him hooked up to machines.
Consider an extubation understanding that he would die once the machine is removed.

## Slide 19
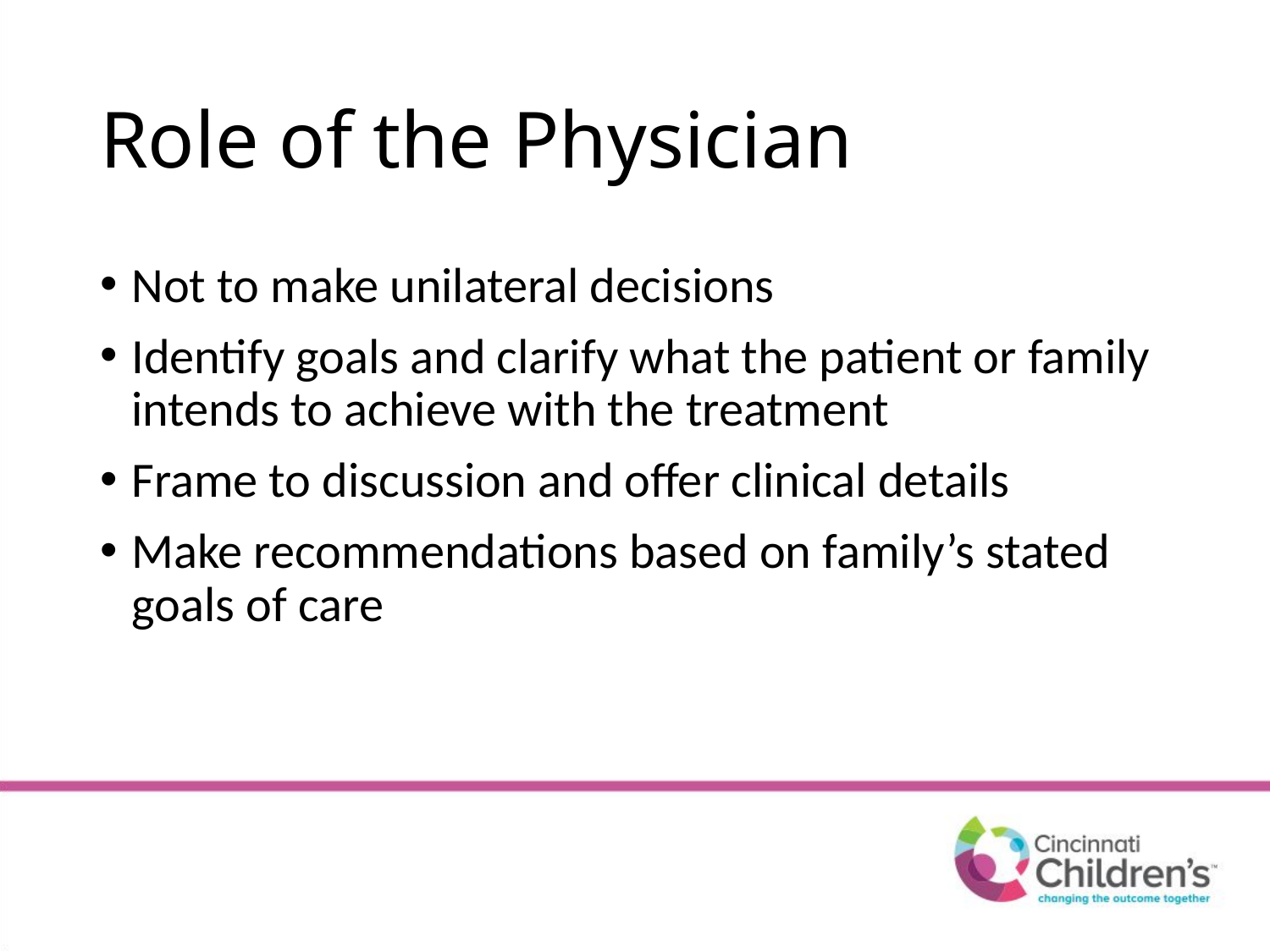

# Role of the Physician
Not to make unilateral decisions
Identify goals and clarify what the patient or family intends to achieve with the treatment
Frame to discussion and offer clinical details
Make recommendations based on family’s stated goals of care

## Slide 20
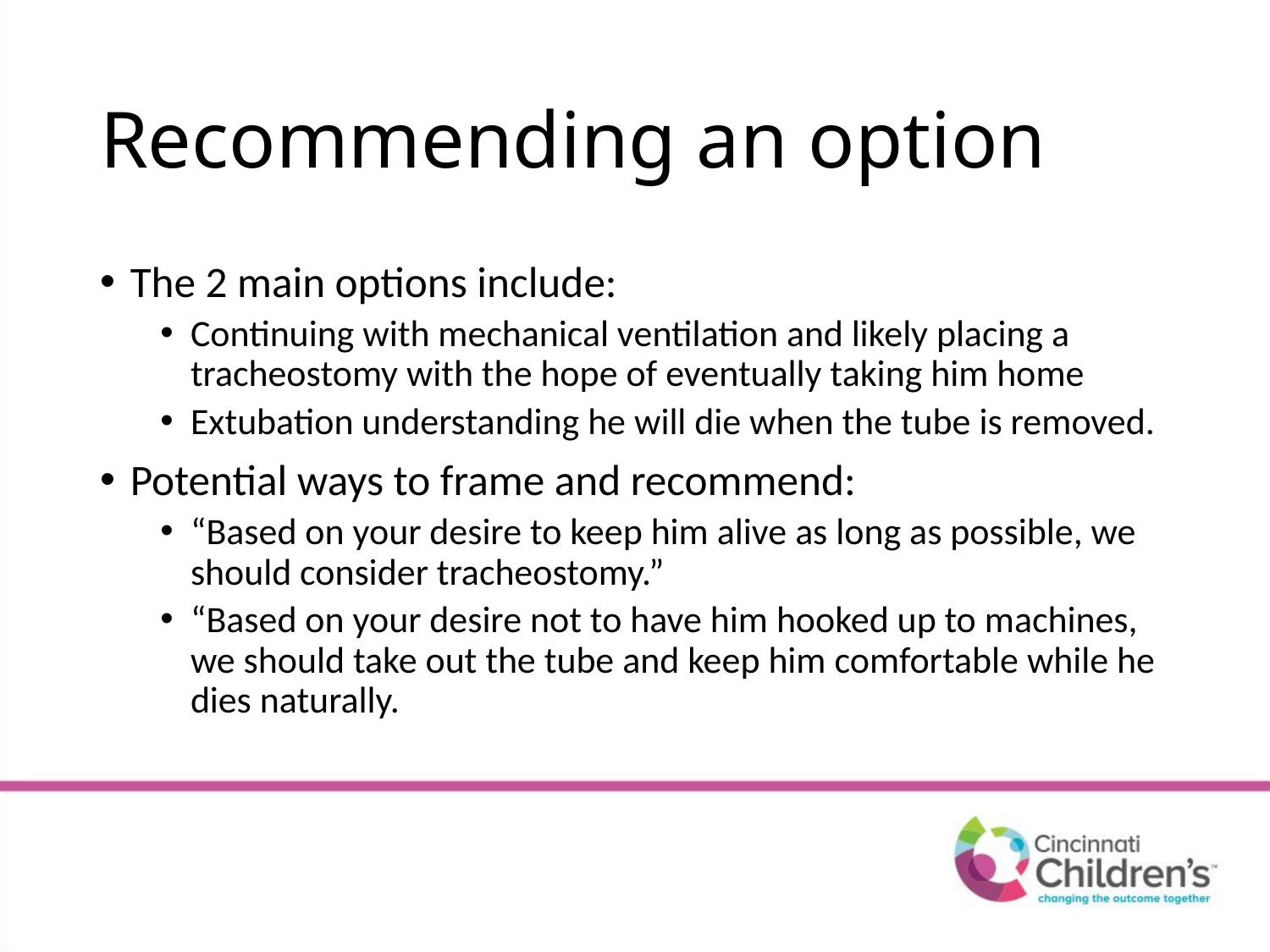

# Recommending an option
The 2 main options include:
Continuing with mechanical ventilation and likely placing a tracheostomy with the hope of eventually taking him home
Extubation understanding he will die when the tube is removed.
Potential ways to frame and recommend:
“Based on your desire to keep him alive as long as possible, we should consider tracheostomy.”
“Based on your desire not to have him hooked up to machines, we should take out the tube and keep him comfortable while he dies naturally.

## Slide 21
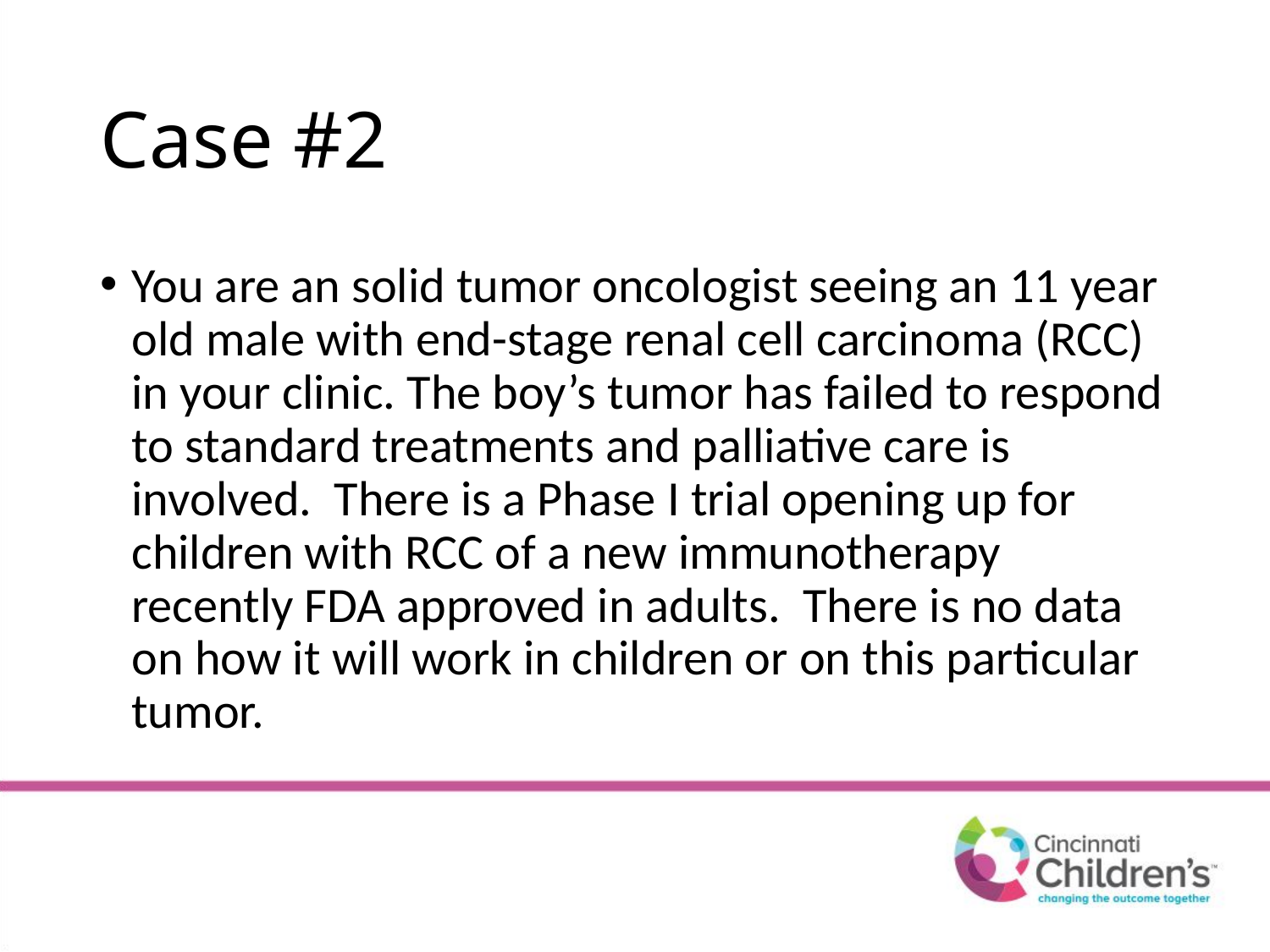

# Case #2
You are an solid tumor oncologist seeing an 11 year old male with end-stage renal cell carcinoma (RCC) in your clinic. The boy’s tumor has failed to respond to standard treatments and palliative care is involved. There is a Phase I trial opening up for children with RCC of a new immunotherapy recently FDA approved in adults. There is no data on how it will work in children or on this particular tumor.

## Slide 22
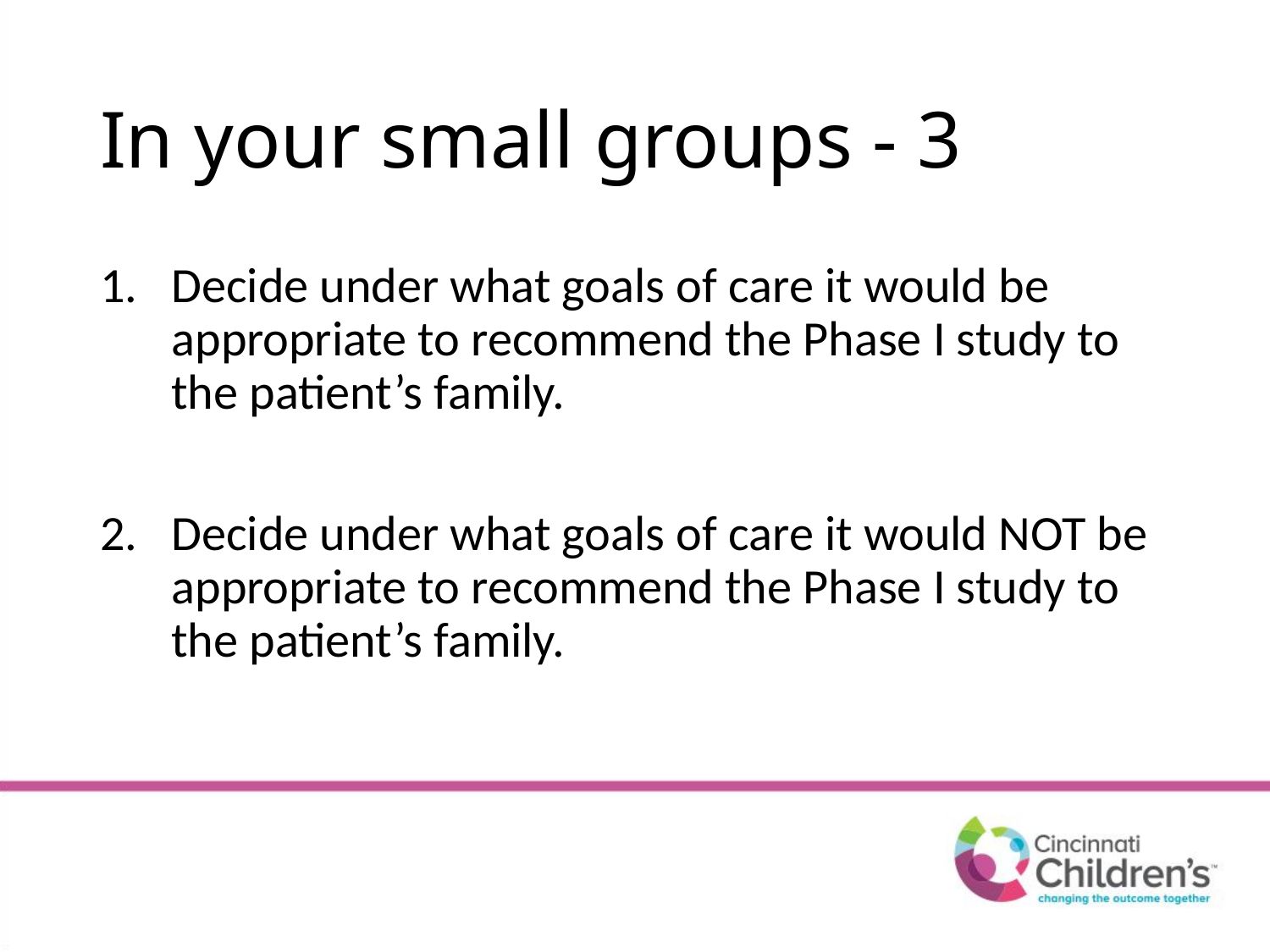

# In your small groups - 3
Decide under what goals of care it would be appropriate to recommend the Phase I study to the patient’s family.
Decide under what goals of care it would NOT be appropriate to recommend the Phase I study to the patient’s family.

## Slide 23
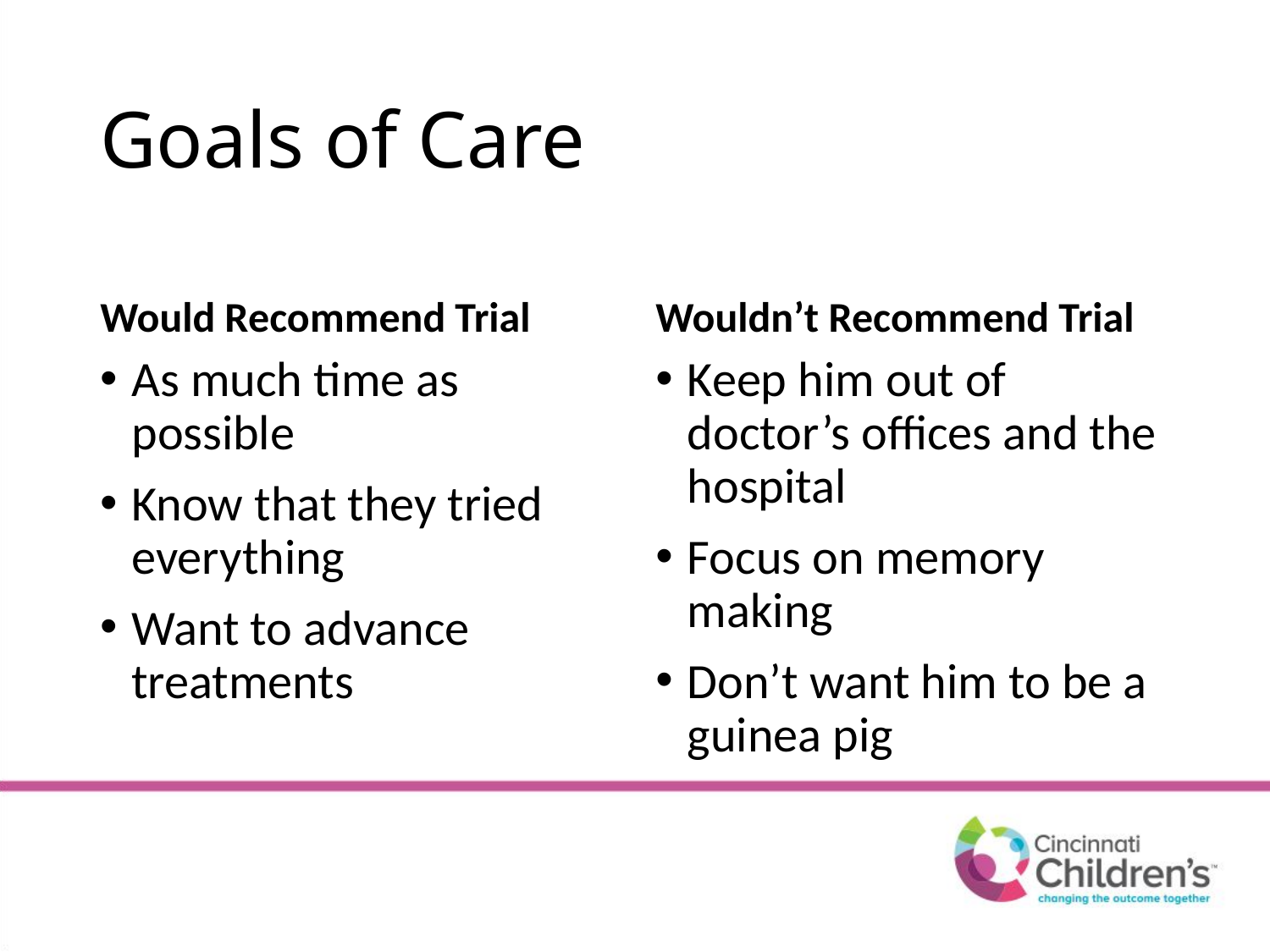

# Goals of Care
Would Recommend Trial
Wouldn’t Recommend Trial
As much time as possible
Know that they tried everything
Want to advance treatments
Keep him out of doctor’s offices and the hospital
Focus on memory making
Don’t want him to be a guinea pig

## Slide 24
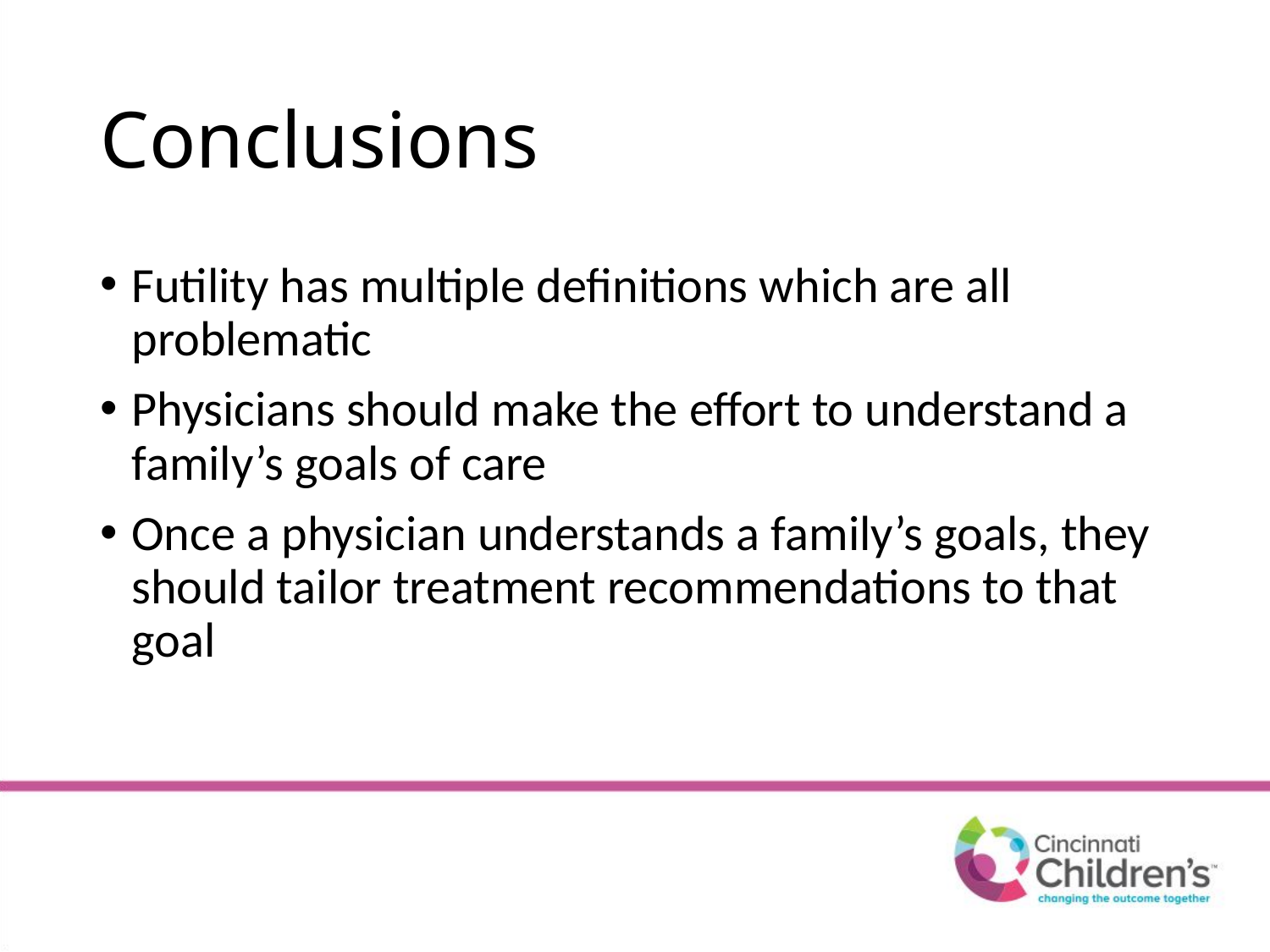

# Conclusions
Futility has multiple definitions which are all problematic
Physicians should make the effort to understand a family’s goals of care
Once a physician understands a family’s goals, they should tailor treatment recommendations to that goal

## Slide 25
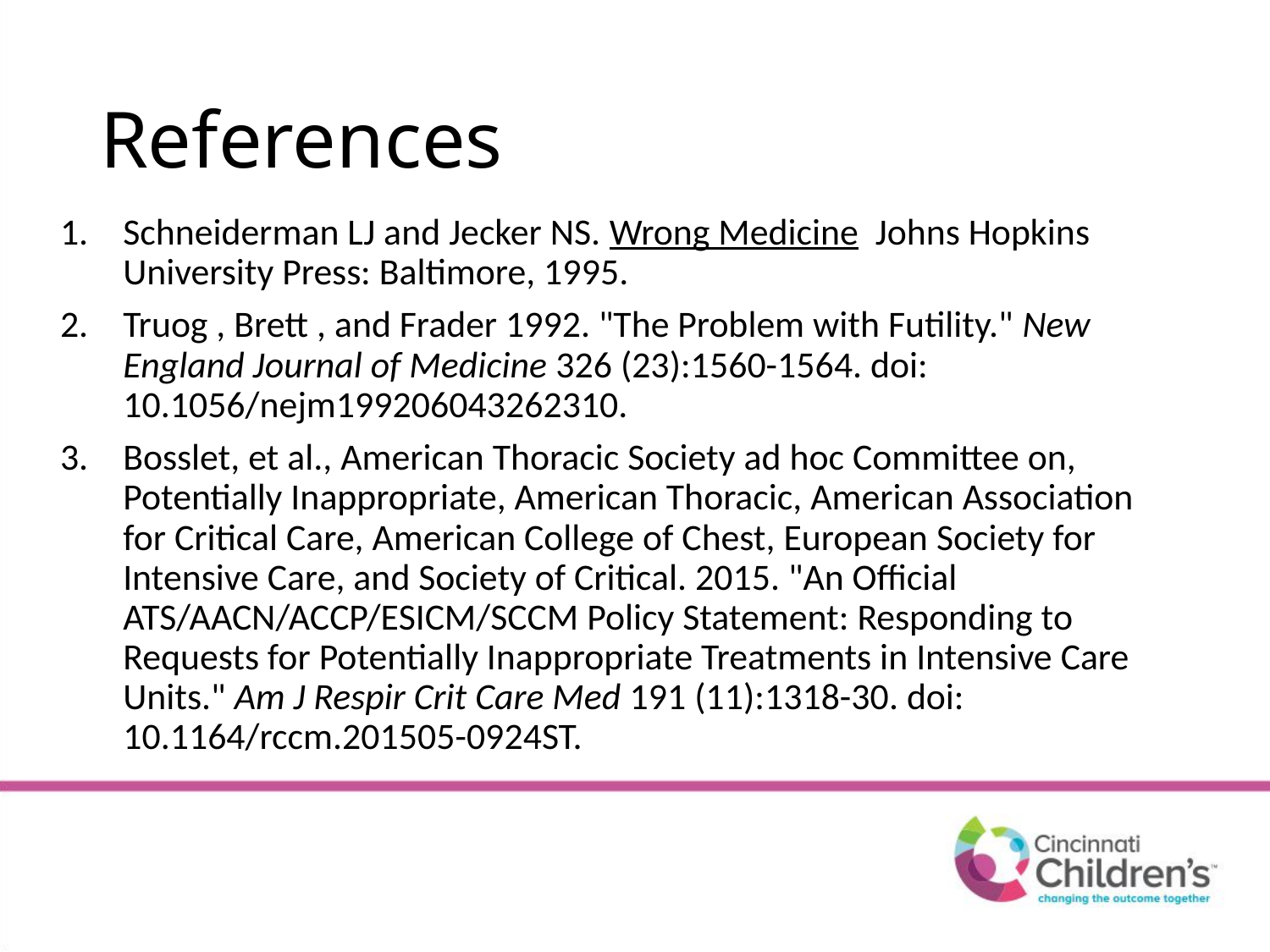

# References
Schneiderman LJ and Jecker NS. Wrong Medicine Johns Hopkins University Press: Baltimore, 1995.
Truog , Brett , and Frader 1992. "The Problem with Futility." New England Journal of Medicine 326 (23):1560-1564. doi: 10.1056/nejm199206043262310.
Bosslet, et al., American Thoracic Society ad hoc Committee on, Potentially Inappropriate, American Thoracic, American Association for Critical Care, American College of Chest, European Society for Intensive Care, and Society of Critical. 2015. "An Official ATS/AACN/ACCP/ESICM/SCCM Policy Statement: Responding to Requests for Potentially Inappropriate Treatments in Intensive Care Units." Am J Respir Crit Care Med 191 (11):1318-30. doi: 10.1164/rccm.201505-0924ST.
