# Supplementary material for: Ethical Dilemmas at the Beginning and End of Life: A Needs-Based, Experience-Informed, Small-Group, Case-Based Curriculum for Pediatric Residents
Source: MedEdPORTAL. 2020 Apr 3;16:10895. doi: 10.15766/mep_2374-8265.10895 (PMC7187913; doi:10.15766/mep_2374-8265.10895)
Supplement: Supplementary file 1 — Medically Provided Fluids Nutrition PowerPoint.pptxMedically Provided Fluids Nutrition Instructor Guide.docxMedically Provided Fluids Nutrition Handout.docxMedically Provided Fluids Nutrition Assessment Questions.docxFutility and Goals of Care PowerPoint.pptxFutility and Goals of Care Instructor Guide.docxFutility and Goals of Care Handout.docxFutility and Goals of Care Assessment Questions.docxEthical Issues in Neonatology PowerPoint.pptxEthical Issues in Neonatology Instructor Guide.docxEthical Issues in Neonatology Assessment Questions.docx [file mep-16-10895-s001.zip › F. Futility and Goals of Care Instructor Guide.docx]

**Futility and Goals of Care
Instructor Directions**

*Set-up:*

- Table Set-up: The group leader should arrange the classroom in multiple individual tables with seats for 6-10 learners.
- Learner Mix: If this is a mixed group of learners (i.e. medical students, interns, upper level residents), there should be a mix of all learners at each table. If possible, an attending physician who has encountered this or a similar challenge, should also be present at the table to provide additional guidance.
- Assessment Tools: Pre and Post Session evaluations should be printed double sided and placed on individual tables. Learners should be instructed to complete the pre-session evaluation when they arrive. They should be asked to complete the post-session evaluation after the session is completed.
- Handouts: Handouts should be printed single sided and placed on the tables prior to the session. For this session, each table should receive only 1 of the 4 available quotes. Learners should be instructed that the will be directed when to utilize the handouts during the session.

*Instructor Overview:*

- Before Session Begins: Prior to the start of the session, the session leader should ask the learners to complete the pre-session assessment.
- Presentation: The session leader will read though the PowerPoint presentation specifically providing details about the case.
- Breakout Sessions: Each time the leader reaches a slide titled “In Your Small Groups” the leader will prompt the teams to discuss the questions on the slide and utilize a hand out if one is available for that section. During each breakout session allow 3-10 minutes for discussion as a small group and 2-3 minutes for groups to share their thoughts with the larger group. Ensure every group gets to report out at least once, but not necessarily for each breakout session. Time for each session should be geared towards the amount of time available to complete the module. For a 45 minute session, 5 minutes for discussion of the 1^st^ and 3^rd^ breakout sessions and 10 minutes for discussion of the 2^nd^ breakout session and 3 minutes for report out is sufficient. **If you have time constraints, you can skip Case #2 (slides 21-23).** Following residents/learners reporting out when they think/feel about each discussion questions, the leader should continue with the slides to provide didactic and context for the questions addressed.
- Conclusion of Session: As the learners to complete the post-session assessment at the end of the session.

*Additional Session-Specific Instructions and Content Information:*

**“In Your Small Groups – 1”:** The instructor should prompt the learners to break into small groups to discuss the question on the slide.

**Question 1, Slides 4 - 6:** The short answer is to the question of which scenarios are futile is “it depends”. The instructor should utilize the below information for additional context to describe the cases on slide 5. Slide 6 further outlines 3 potential ways to consider futility which will be described further during the slides.

- Case 1: If the patient/family’s goal is to treat a pneumonia, then antibiotics would likely be effective at meeting that goal and therefore not be considered futile. If the patient/family’s goal is to only pursue a treatment if it would cure the cancer or if the chances of antibiotics working are slim, then antibiotics *could* be considered futile.
- Case 2: If the patient/family’s goal is to keep the patient alive longer, then intubation and mechanical ventilation would likely meet that goal and therefore would not be considered futile. If the goal is to only provide treatments that would improve function or neurologic status or the potential of her surviving with intubation is limited, then intubation *could* be considered futile.
- Case 3: If the patient/family’s goal is to keep the patient alive while awaiting a surgery or procedure to stop the bleeding, then blood products are not futile. If no surgery or procedure is possible, the goal is for the patient to live outside of the ICU, or the hemorrhage is quicker than blood can be administered, then blood products *could* be considered futile.

**Slides 7-12:** These slides provide different definitions of futility.

- Qualitative and Quantitative futility were definitions proposed by Lawrence Schneiderman, a family physician, and Nancy Jecker, a PhD in medical history in a book published in 1995 called “Wrong Medicine”.
- For Slides 8 and 9, the examples under each definition are the ones that Schneider and Jecker describe in their book. As described on both slides, while these were acceptable thresholds to these individual authors, not all physicians or patients would agree.
- For Slide 10, Authors Truog, Brett, and Frader attempted to provide an objective definition of futility, but it is very limited because physiologically a treatment may be successful for at least a short time or could possibly work.

**“In Your Small Groups – 2”:** The instructor should prompt the learners to break into small groups to discuss the questions on the slide. The instructor should prompt the learners to utilize the patient goal on their table.

**Question 1:** For report out, go through slides 15-18 and ask the table what they discussed. There are no right or wrong answers. After they report out you can forward the slide to include some potential options. You should also ask them how they would frame options based on those goals during the report out.

**Question 2:** Utilize slide 19 and 20 to explain the role of the physician, potential goals, and ways to frame and recommend options. Additional ways to frame and recommend options include:

- You stated you want him to be comfortable and you do not feel the ventilator is comfortable. We do not have a way to keep him awake and comfortable on the ventilator. Because of this, our only way to achieve that goal, is to keep him comfortable with medications, take him off the ventilator, and die naturally.
- You stated your goal is for him to be able to spend some time at home. One way to achieve this is to place a tracheostomy and a ventilator. Our team can usually get children home within a couple of months.

**Case #2:** This case (slides 21-23) can be skipped if short on time. Please note, the intent of a phase 1 study is to test for *safety.* While this drug has been FDA approved in adults with RCC, there is no evidence that this drug will improve survival. If the learners are not clear on what a phase 1 trial is, this can be shared with the groups.

**“In Your Small Groups – 3”:** The instructor should prompt the learners to break into small groups to discuss the questions on the slide.

**Question 1 and 2:** Utilize slide 21. Ask the learners to report out before advancing the slide to show answers. Remind the group that the goal of a phase 1 study is *safety* when reviewing the answers if you have not already.

*References:*

The session leader can utilize the references listed to obtain additional content expertise if needed prior to leading the session.
