# Supplementary material for: Ethical Dilemmas at the Beginning and End of Life: A Needs-Based, Experience-Informed, Small-Group, Case-Based Curriculum for Pediatric Residents
Source: MedEdPORTAL. 2020 Apr 3;16:10895. doi: 10.15766/mep_2374-8265.10895 (PMC7187913; doi:10.15766/mep_2374-8265.10895)
Supplement: Supplementary file 1 — Medically Provided Fluids Nutrition PowerPoint.pptxMedically Provided Fluids Nutrition Instructor Guide.docxMedically Provided Fluids Nutrition Handout.docxMedically Provided Fluids Nutrition Assessment Questions.docxFutility and Goals of Care PowerPoint.pptxFutility and Goals of Care Instructor Guide.docxFutility and Goals of Care Handout.docxFutility and Goals of Care Assessment Questions.docxEthical Issues in Neonatology PowerPoint.pptxEthical Issues in Neonatology Instructor Guide.docxEthical Issues in Neonatology Assessment Questions.docx [file mep-16-10895-s001.zip › G. Futility and Goals of Care Handout.docx]

We want as much time with him as possible.

We want to take him home.

We want him to be comfortable.

We don’t want him hooked up to machines.
