# Supplementary material for: Ethical Dilemmas at the Beginning and End of Life: A Needs-Based, Experience-Informed, Small-Group, Case-Based Curriculum for Pediatric Residents
Source: MedEdPORTAL. 2020 Apr 3;16:10895. doi: 10.15766/mep_2374-8265.10895 (PMC7187913; doi:10.15766/mep_2374-8265.10895)
Supplement: Supplementary file 1 — Medically Provided Fluids Nutrition PowerPoint.pptxMedically Provided Fluids Nutrition Instructor Guide.docxMedically Provided Fluids Nutrition Handout.docxMedically Provided Fluids Nutrition Assessment Questions.docxFutility and Goals of Care PowerPoint.pptxFutility and Goals of Care Instructor Guide.docxFutility and Goals of Care Handout.docxFutility and Goals of Care Assessment Questions.docxEthical Issues in Neonatology PowerPoint.pptxEthical Issues in Neonatology Instructor Guide.docxEthical Issues in Neonatology Assessment Questions.docx [file mep-16-10895-s001.zip › H. Futility and Goals of Care Assessment Questions.docx]

Palliative Care and Medical Ethics Curriculum Evaluation
Futility and Goals of Care

**Level of Training:**

M3 M4/AI PGY1 PGY2 PGY3 PGY4 PGY5 Other _________________________

**Pre-Conference Evaluation:**

I am comfortable that I understand how the term futility is used in a clinical context.

1 = strongly disagree 2 = disagree 3 = neutral 4 = agree 5 = strongly agree

I am comfortable recommending a treatment plan based on a patient or family’s goals of care.

1 = strongly disagree 2 = disagree 3 = neutral 4 = agree 5 = strongly agree

Palliative Care and Medical Ethics Curriculum Evaluation
Futility and Goals of Care

**Post-Conference Evaluation:**

I am comfortable that I understand how the term futility is used in a clinical context.

1 = strongly disagree 2 = disagree 3 = neutral 4 = agree 5 = strongly agree

I am comfortable recommending a treatment plan based on a patient or family’s goals of care.

1 = strongly disagree 2 = disagree 3 = neutral 4 = agree 5 = strongly agree

What was most effective about this session?

What are areas for improvement of this session?
