# Supplementary material for: Ethical Dilemmas at the Beginning and End of Life: A Needs-Based, Experience-Informed, Small-Group, Case-Based Curriculum for Pediatric Residents
Source: MedEdPORTAL. 2020 Apr 3;16:10895. doi: 10.15766/mep_2374-8265.10895 (PMC7187913; doi:10.15766/mep_2374-8265.10895)
Supplement: Supplementary file 1 — Medically Provided Fluids Nutrition PowerPoint.pptxMedically Provided Fluids Nutrition Instructor Guide.docxMedically Provided Fluids Nutrition Handout.docxMedically Provided Fluids Nutrition Assessment Questions.docxFutility and Goals of Care PowerPoint.pptxFutility and Goals of Care Instructor Guide.docxFutility and Goals of Care Handout.docxFutility and Goals of Care Assessment Questions.docxEthical Issues in Neonatology PowerPoint.pptxEthical Issues in Neonatology Instructor Guide.docxEthical Issues in Neonatology Assessment Questions.docx [file mep-16-10895-s001.zip › I. Ethical Issues in Neonatology PowerPoint.pptx]

## Slide 1
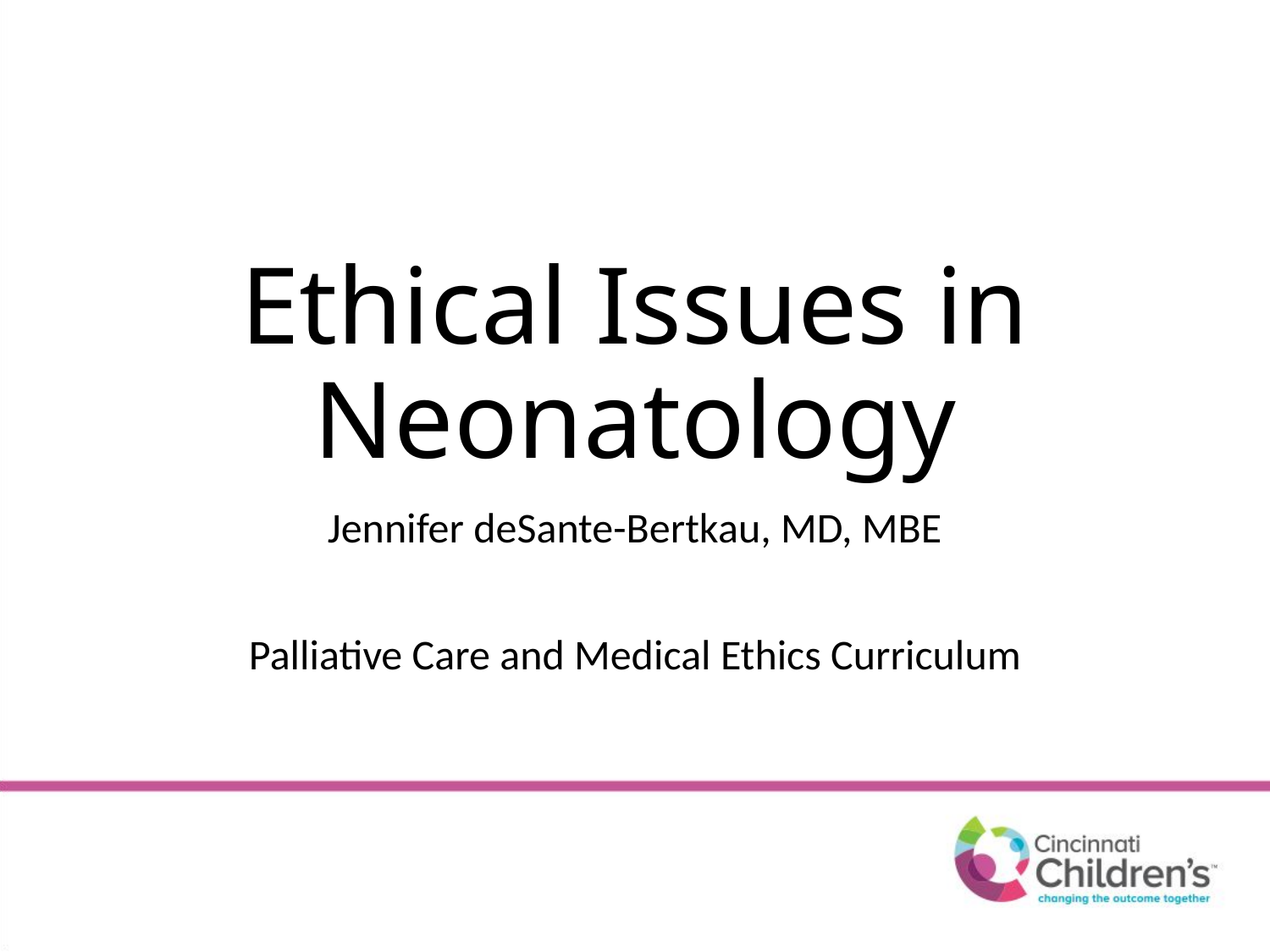

# Ethical Issues in Neonatology
Jennifer deSante-Bertkau, MD, MBE
Palliative Care and Medical Ethics Curriculum

## Slide 2
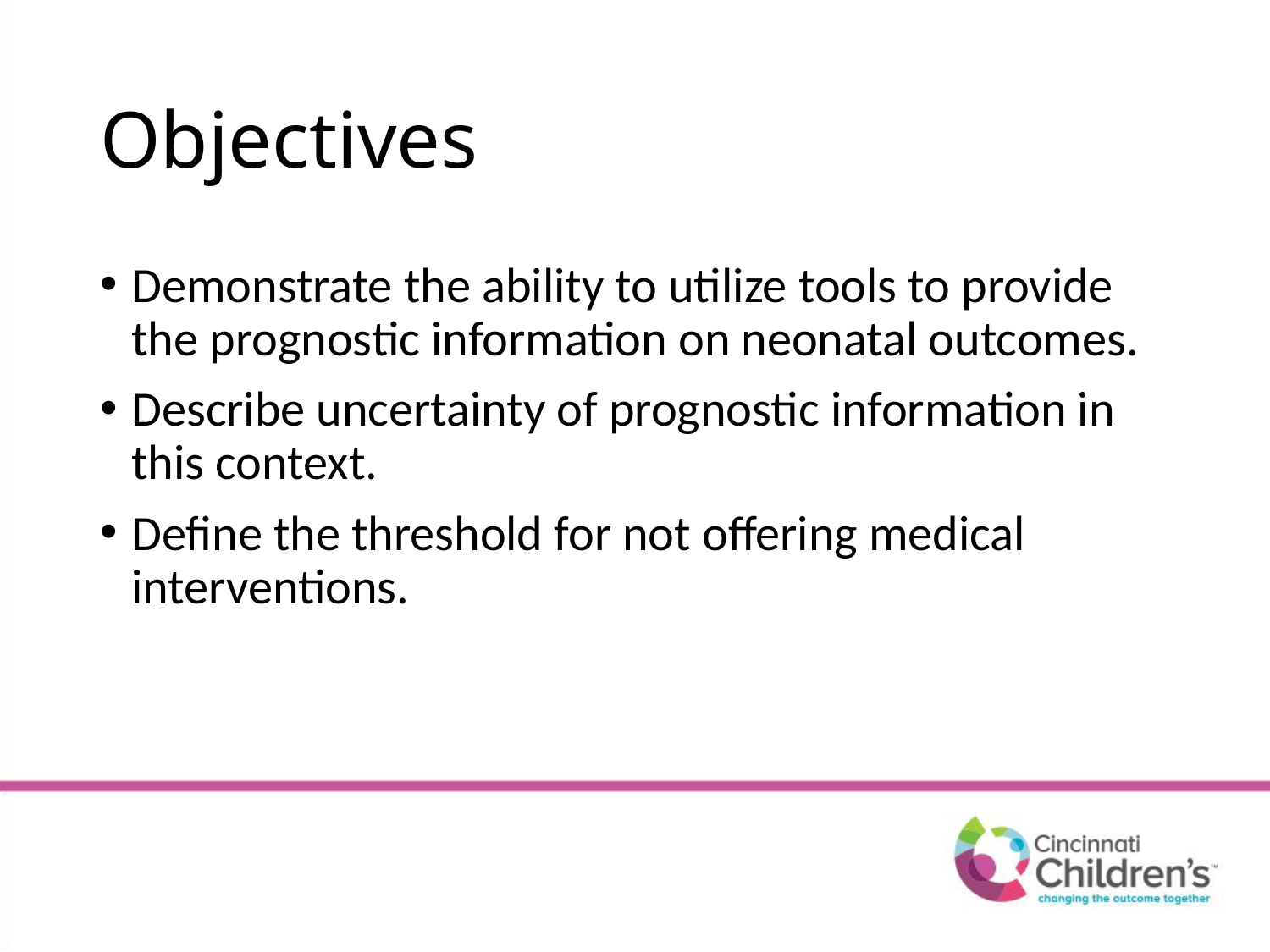

# Objectives
Demonstrate the ability to utilize tools to provide the prognostic information on neonatal outcomes.
Describe uncertainty of prognostic information in this context.
Define the threshold for not offering medical interventions.

## Slide 3
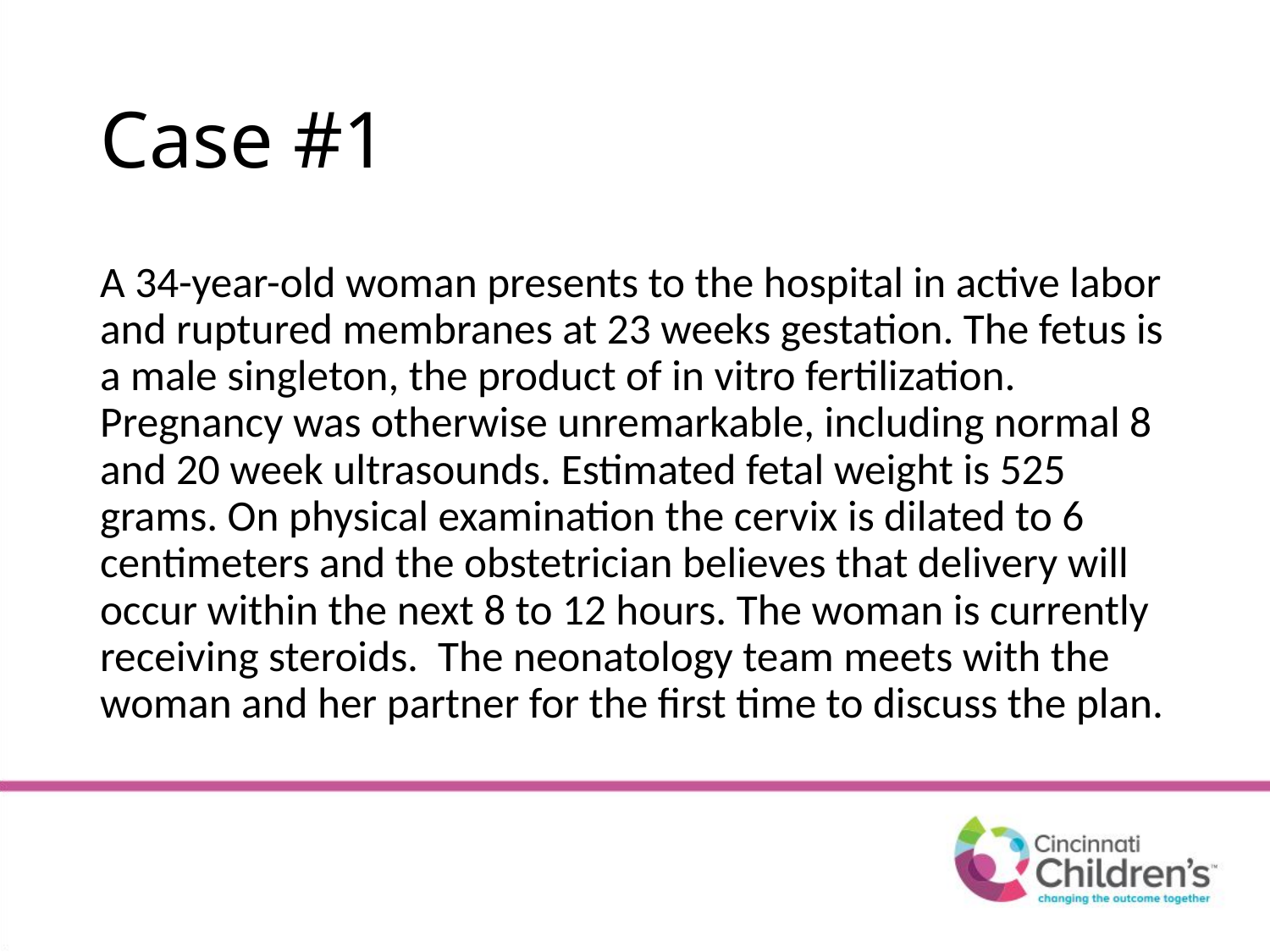

# Case #1
A 34-year-old woman presents to the hospital in active labor and ruptured membranes at 23 weeks gestation. The fetus is a male singleton, the product of in vitro fertilization. Pregnancy was otherwise unremarkable, including normal 8 and 20 week ultrasounds. Estimated fetal weight is 525 grams. On physical examination the cervix is dilated to 6 centimeters and the obstetrician believes that delivery will occur within the next 8 to 12 hours. The woman is currently receiving steroids. The neonatology team meets with the woman and her partner for the first time to discuss the plan.

## Slide 4
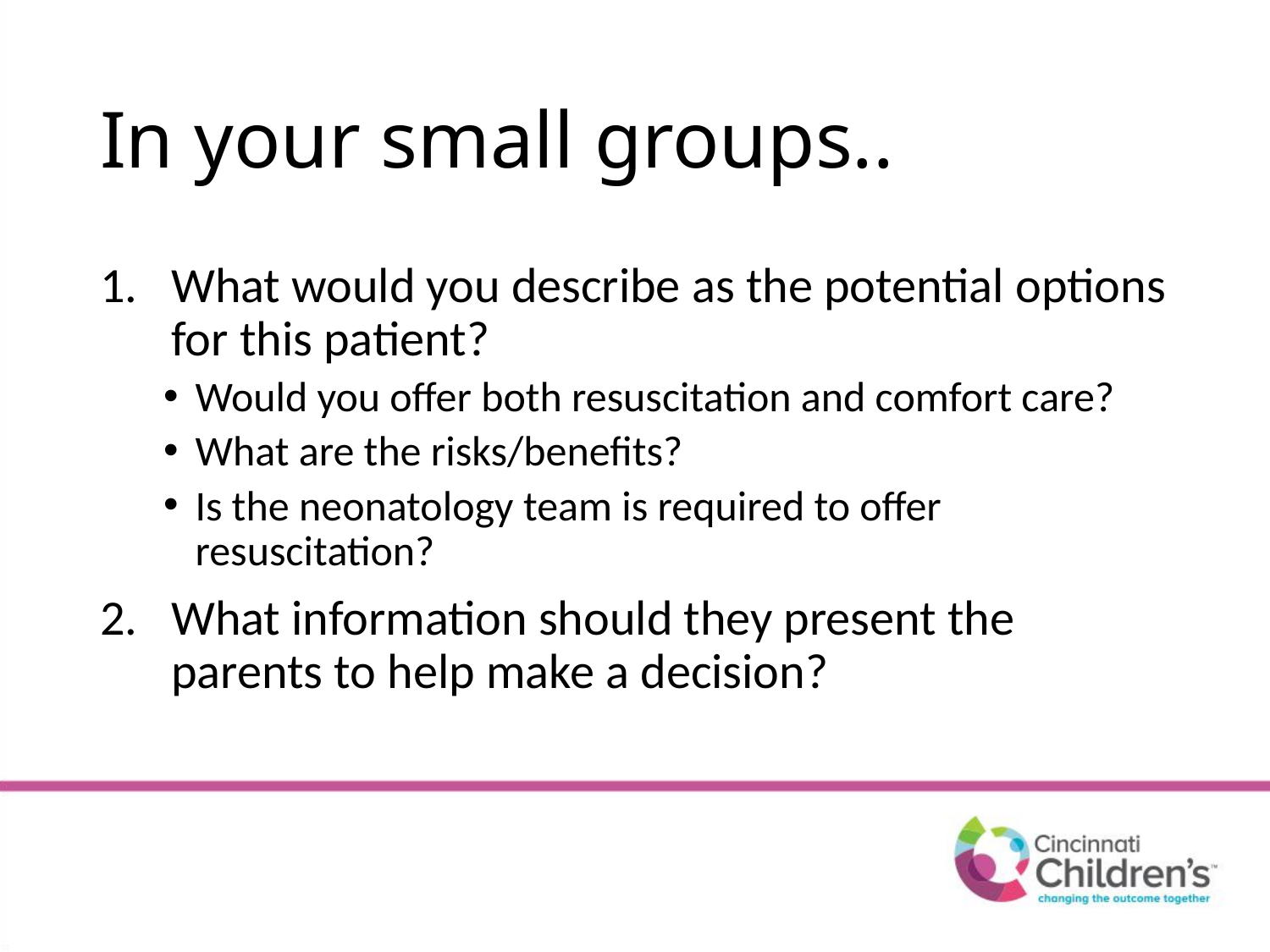

# In your small groups..
What would you describe as the potential options for this patient?
Would you offer both resuscitation and comfort care?
What are the risks/benefits?
Is the neonatology team is required to offer resuscitation?
What information should they present the parents to help make a decision?

## Slide 5
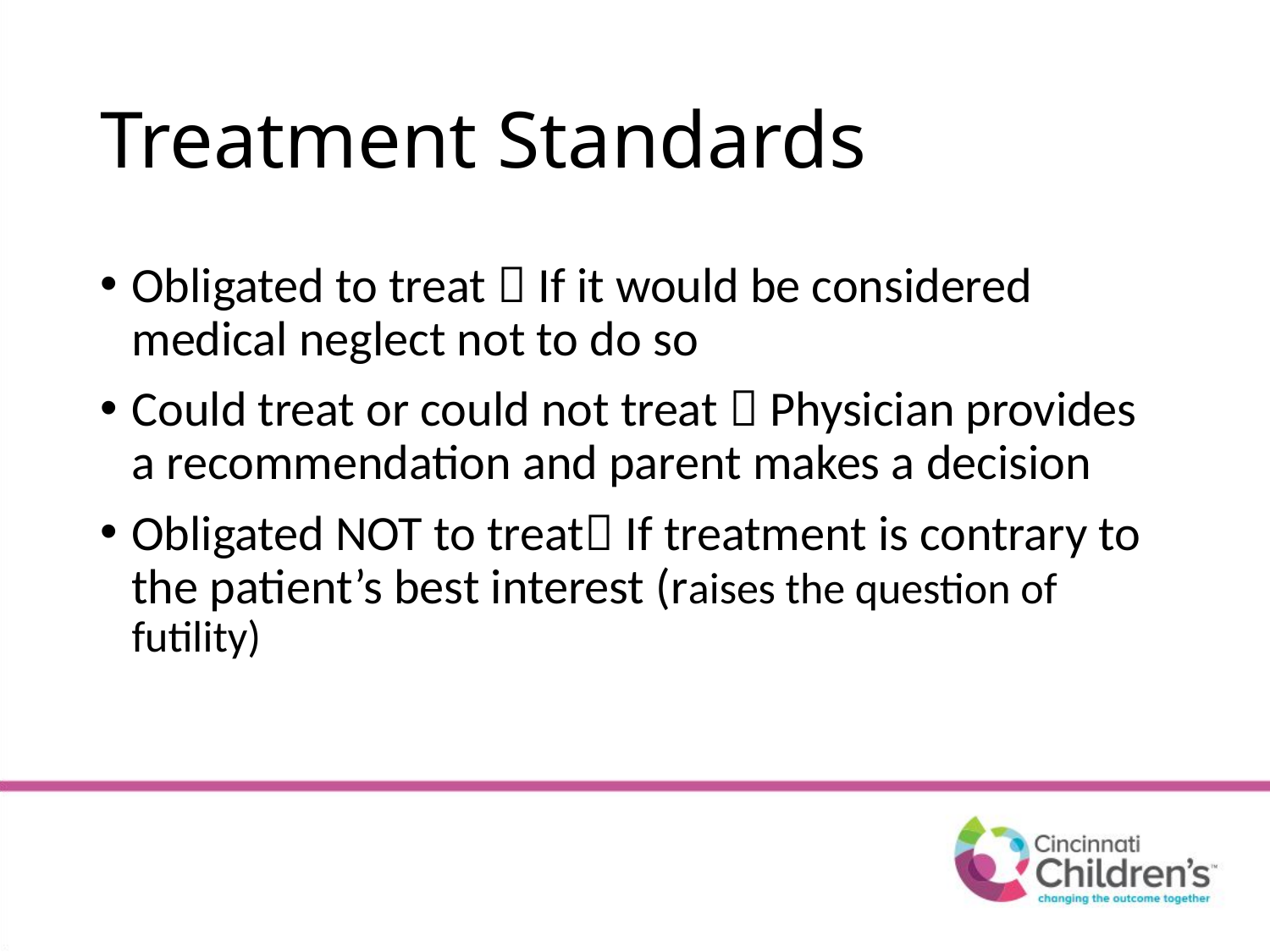

# Treatment Standards
Obligated to treat  If it would be considered medical neglect not to do so
Could treat or could not treat  Physician provides a recommendation and parent makes a decision
Obligated NOT to treat If treatment is contrary to the patient’s best interest (raises the question of futility)

## Slide 6
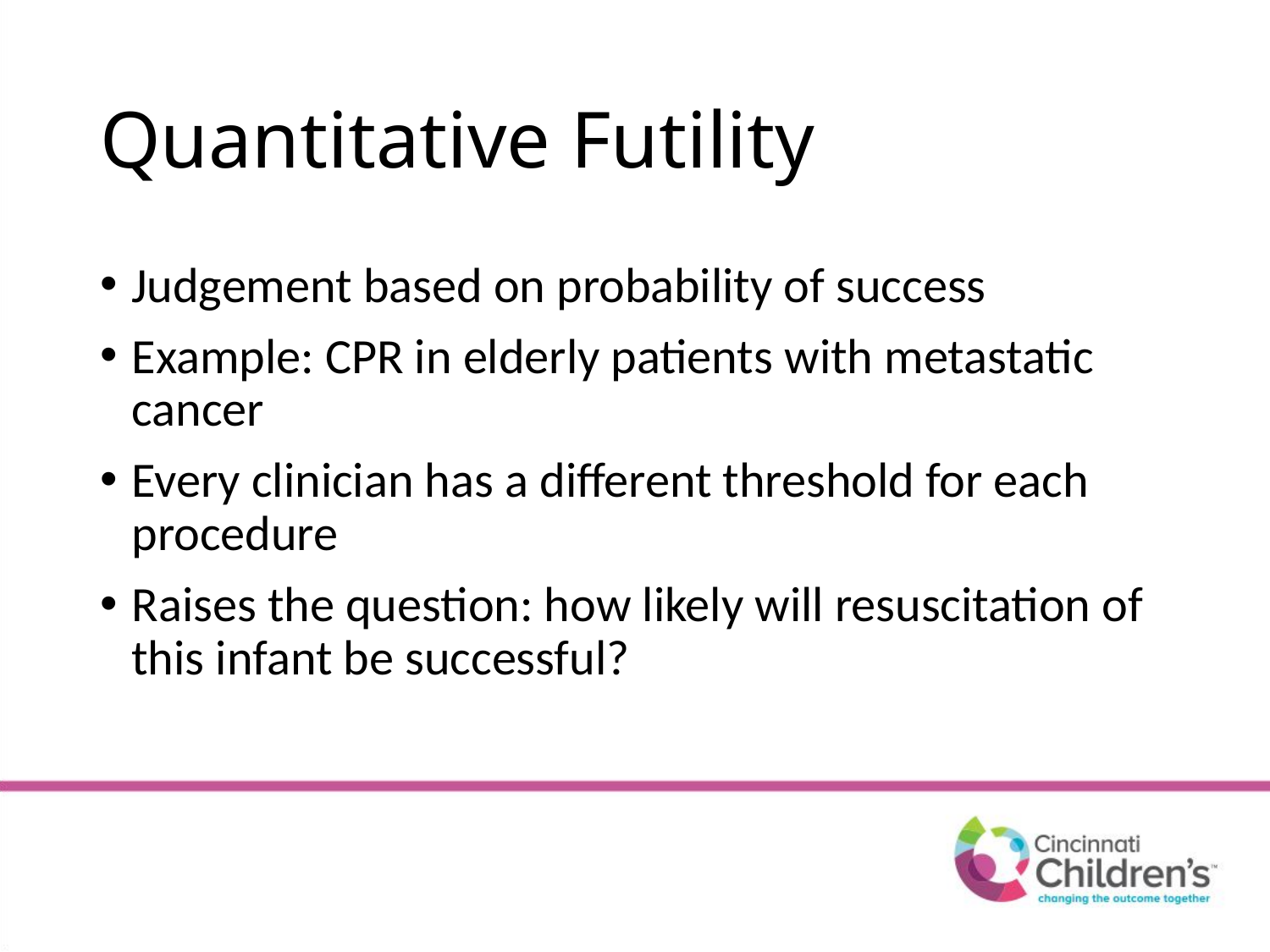

# Quantitative Futility
Judgement based on probability of success
Example: CPR in elderly patients with metastatic cancer
Every clinician has a different threshold for each procedure
Raises the question: how likely will resuscitation of this infant be successful?

## Slide 7
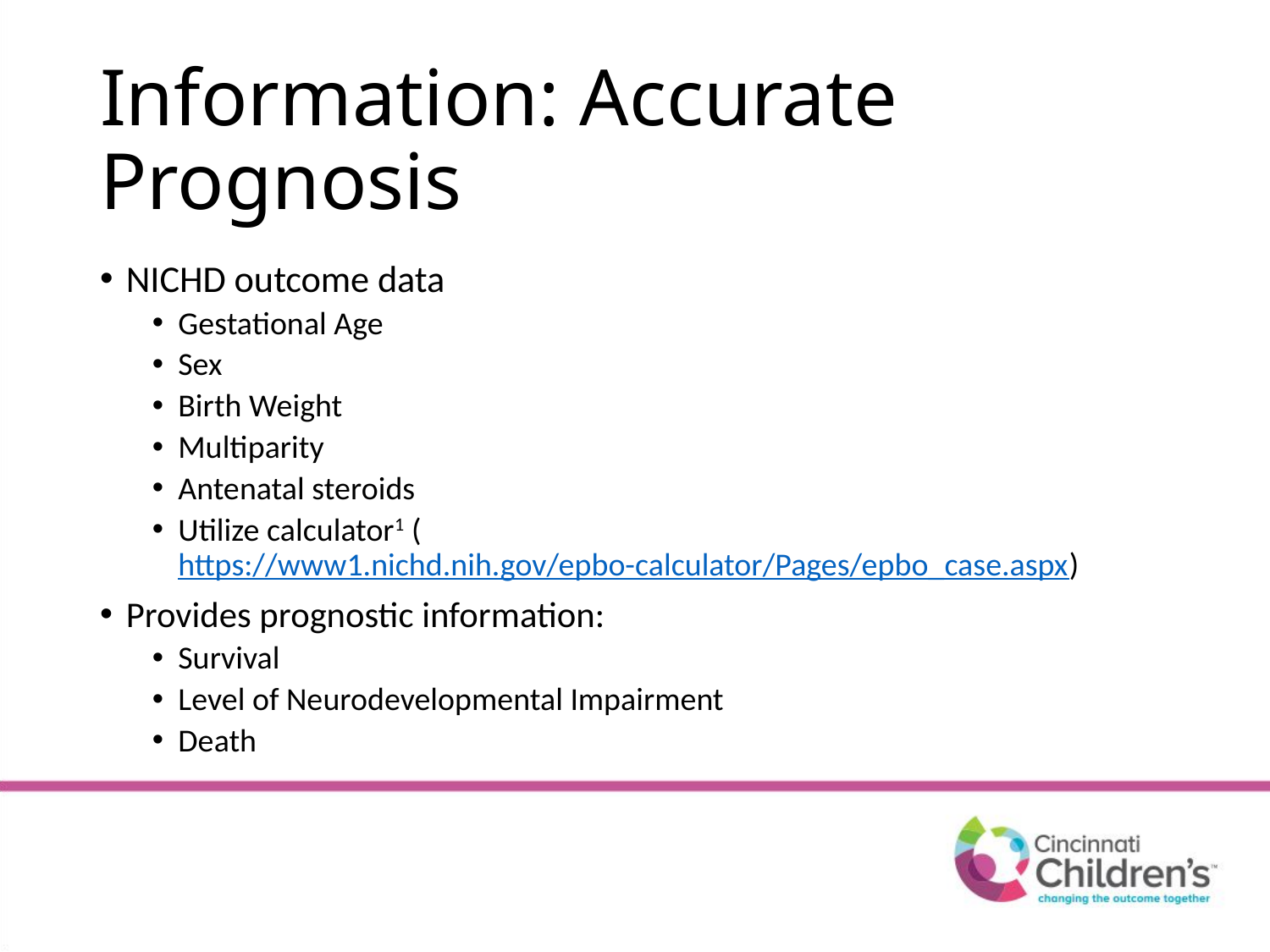

# Information: Accurate Prognosis
NICHD outcome data
Gestational Age
Sex
Birth Weight
Multiparity
Antenatal steroids
Utilize calculator1 (https://www1.nichd.nih.gov/epbo-calculator/Pages/epbo_case.aspx)
Provides prognostic information:
Survival
Level of Neurodevelopmental Impairment
Death

## Slide 8
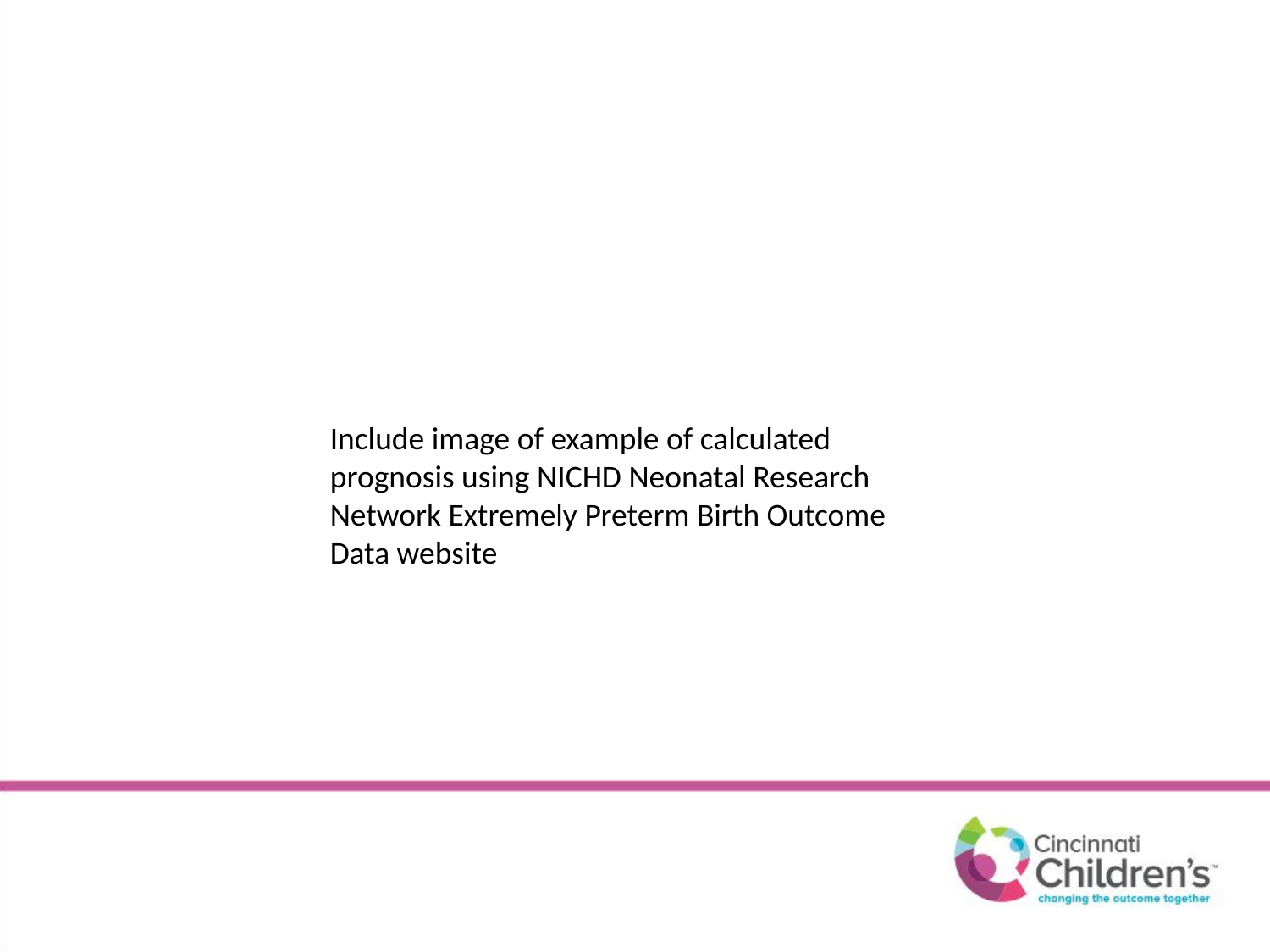

#
Include image of example of calculated prognosis using NICHD Neonatal Research Network Extremely Preterm Birth Outcome Data website

## Slide 9
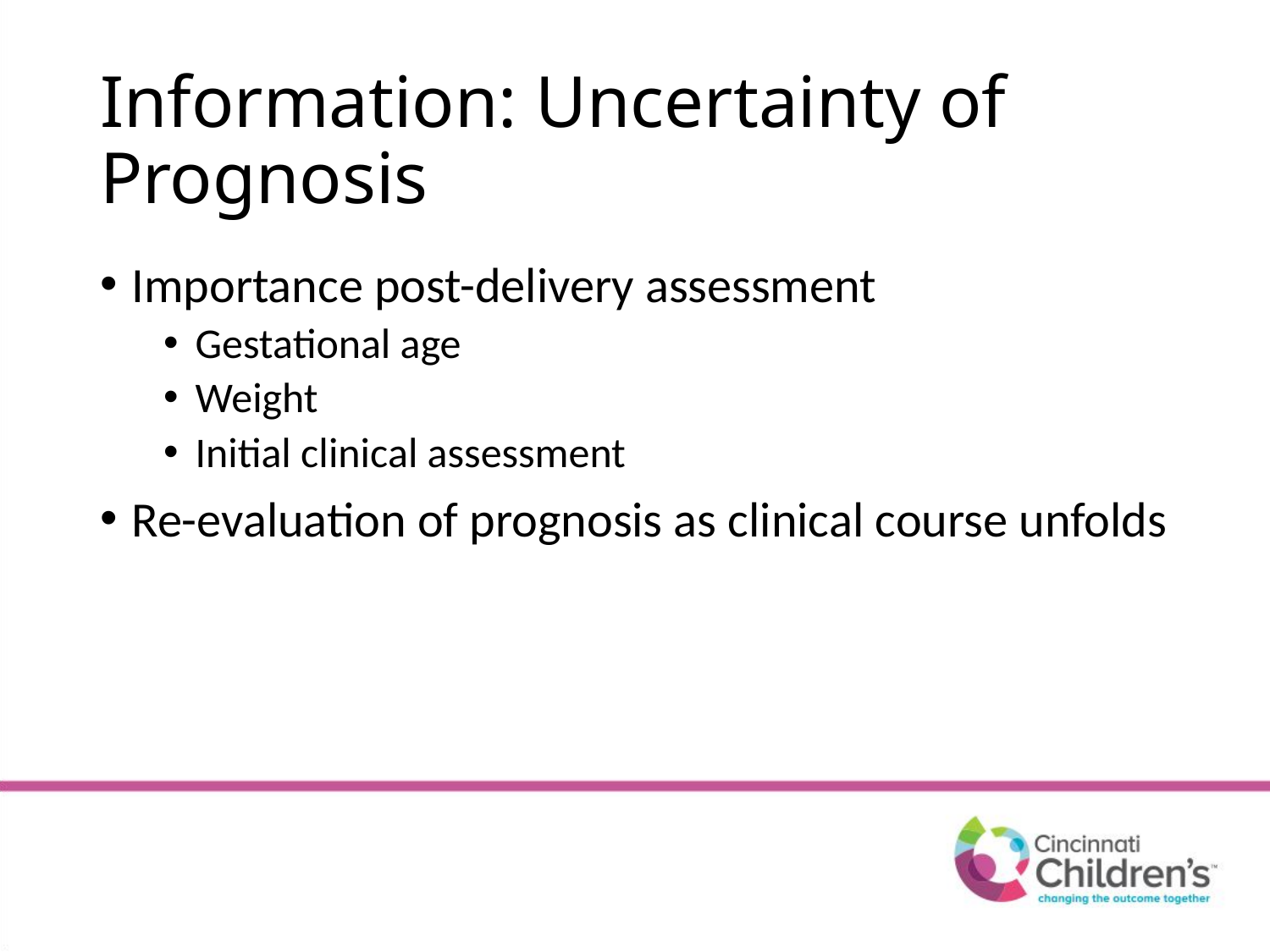

# Information: Uncertainty of Prognosis
Importance post-delivery assessment
Gestational age
Weight
Initial clinical assessment
Re-evaluation of prognosis as clinical course unfolds

## Slide 10
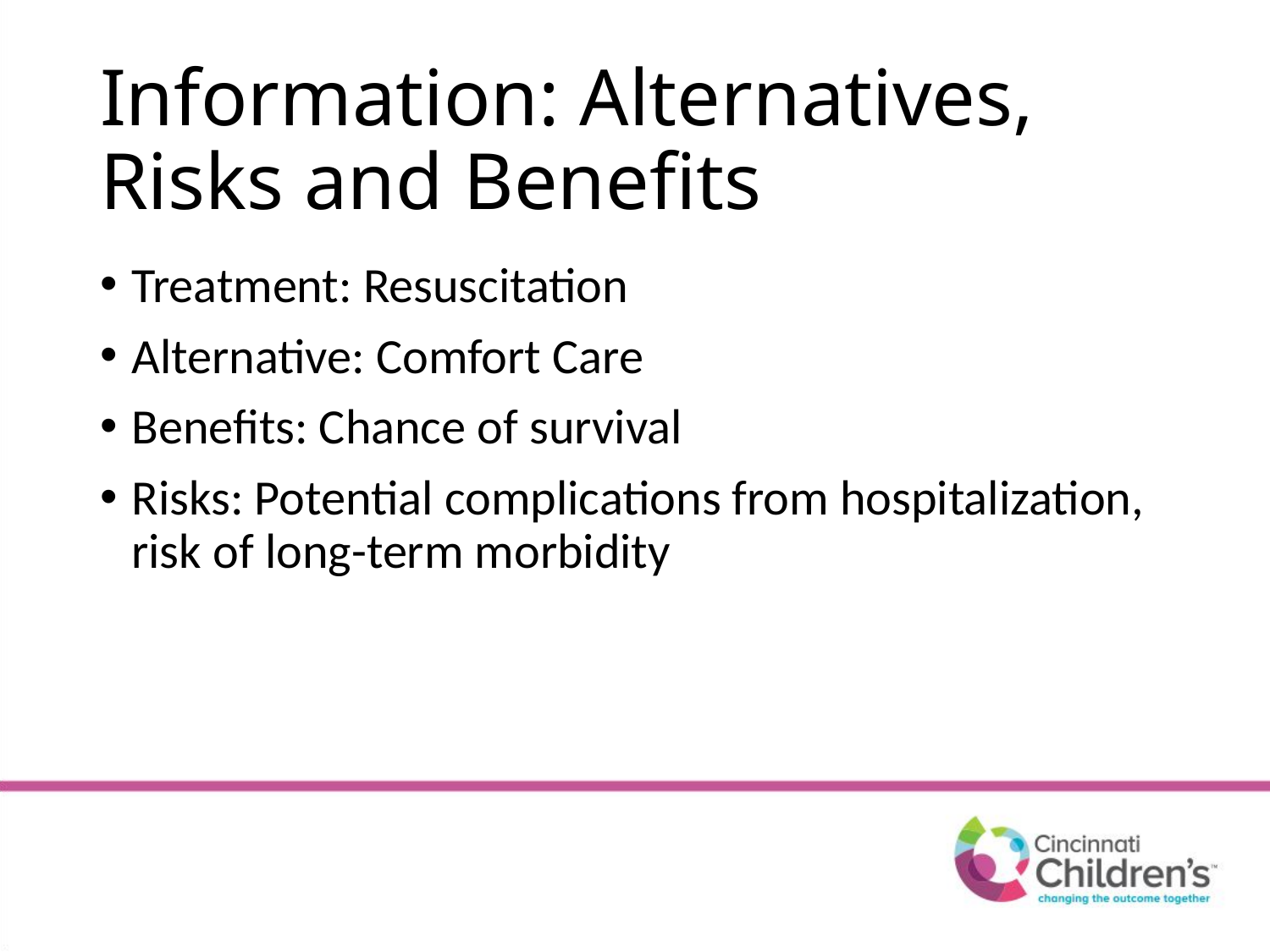

# Information: Alternatives, Risks and Benefits
Treatment: Resuscitation
Alternative: Comfort Care
Benefits: Chance of survival
Risks: Potential complications from hospitalization, risk of long-term morbidity

## Slide 11
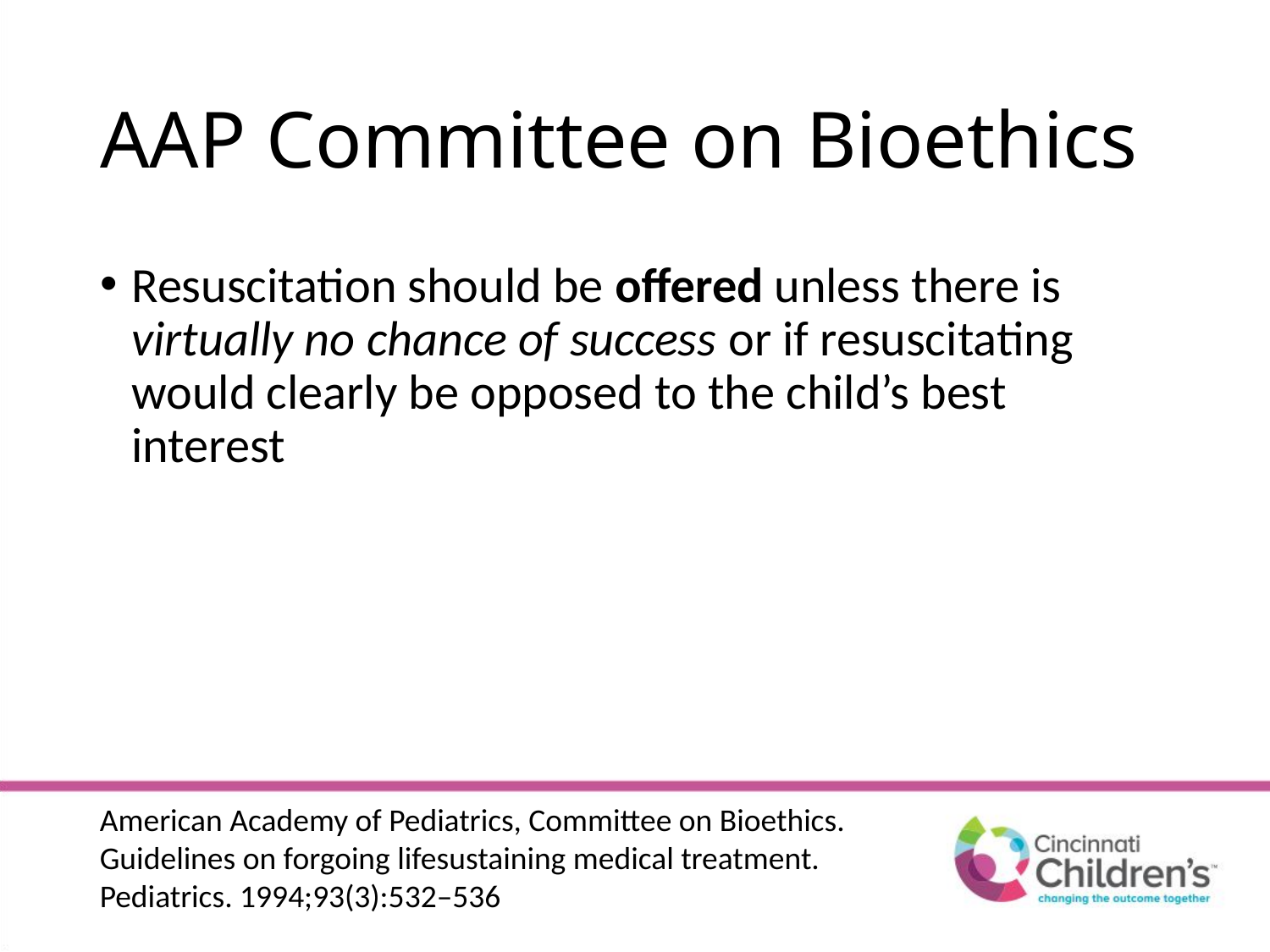

# AAP Committee on Bioethics
Resuscitation should be offered unless there is virtually no chance of success or if resuscitating would clearly be opposed to the child’s best interest
American Academy of Pediatrics, Committee on Bioethics. Guidelines on forgoing lifesustaining medical treatment. Pediatrics. 1994;93(3):532–536

## Slide 12
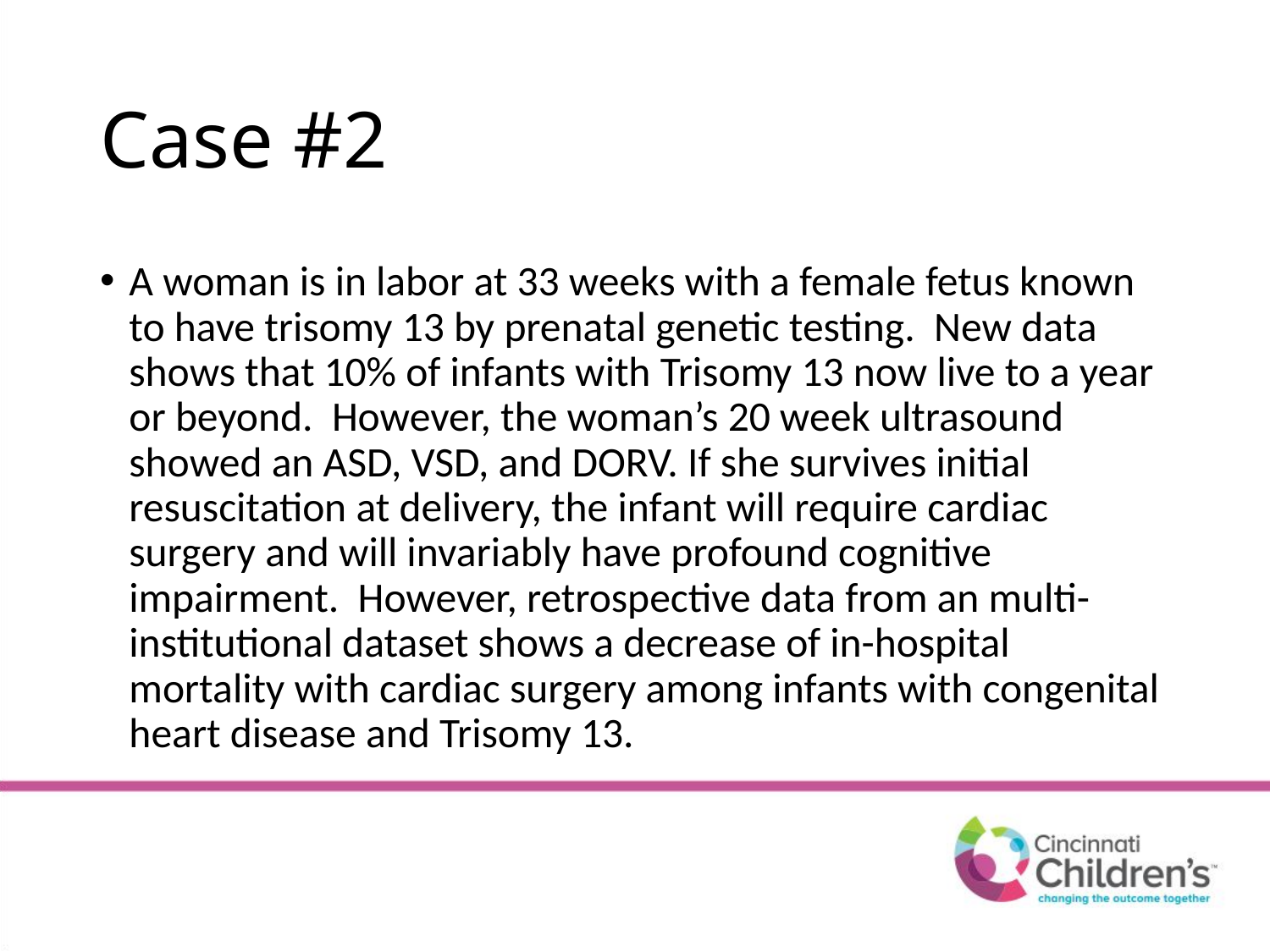

# Case #2
A woman is in labor at 33 weeks with a female fetus known to have trisomy 13 by prenatal genetic testing. New data shows that 10% of infants with Trisomy 13 now live to a year or beyond. However, the woman’s 20 week ultrasound showed an ASD, VSD, and DORV. If she survives initial resuscitation at delivery, the infant will require cardiac surgery and will invariably have profound cognitive impairment. However, retrospective data from an multi-institutional dataset shows a decrease of in-hospital mortality with cardiac surgery among infants with congenital heart disease and Trisomy 13.

## Slide 13
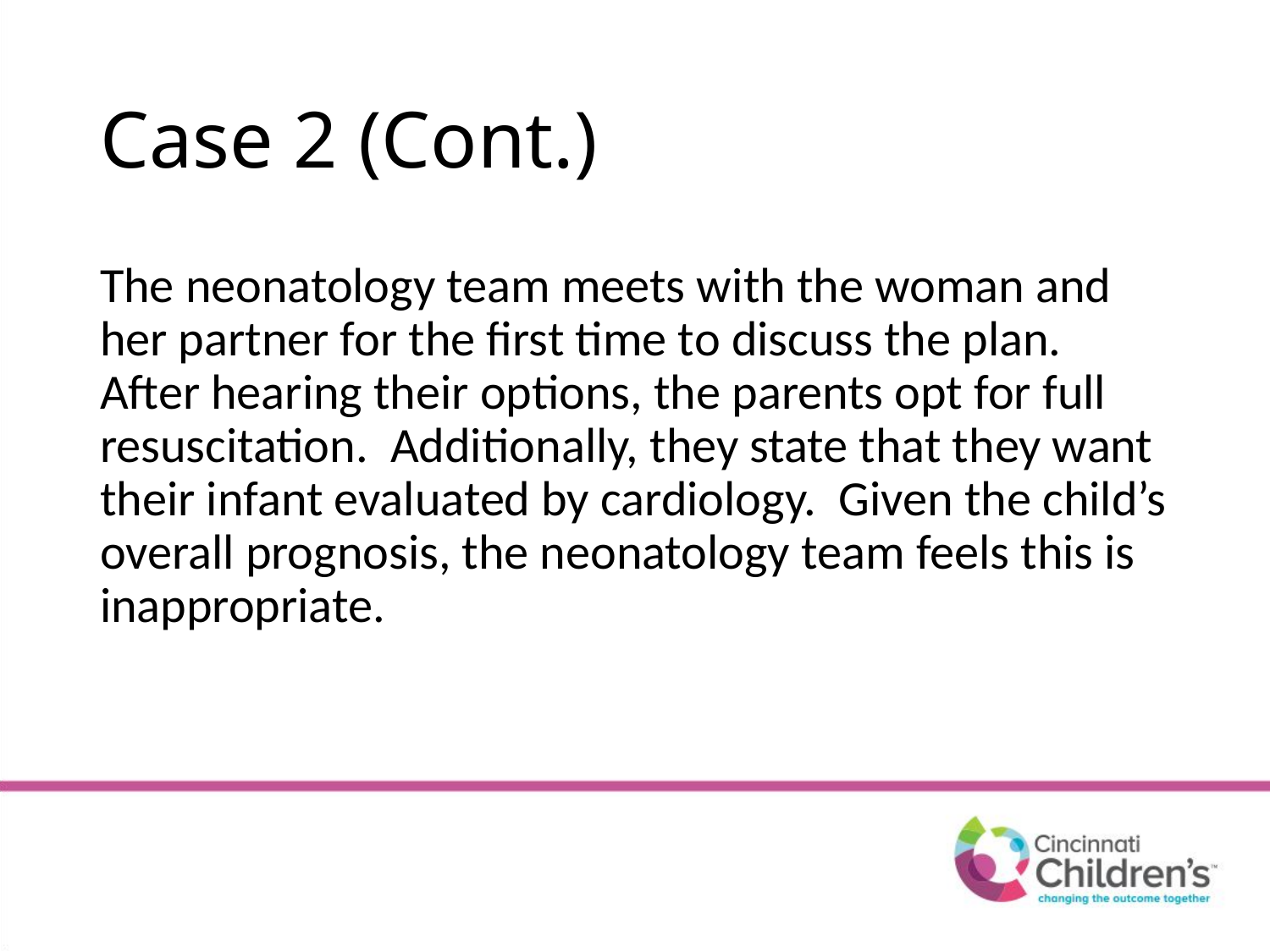

# Case 2 (Cont.)
The neonatology team meets with the woman and her partner for the first time to discuss the plan. After hearing their options, the parents opt for full resuscitation. Additionally, they state that they want their infant evaluated by cardiology. Given the child’s overall prognosis, the neonatology team feels this is inappropriate.

## Slide 14
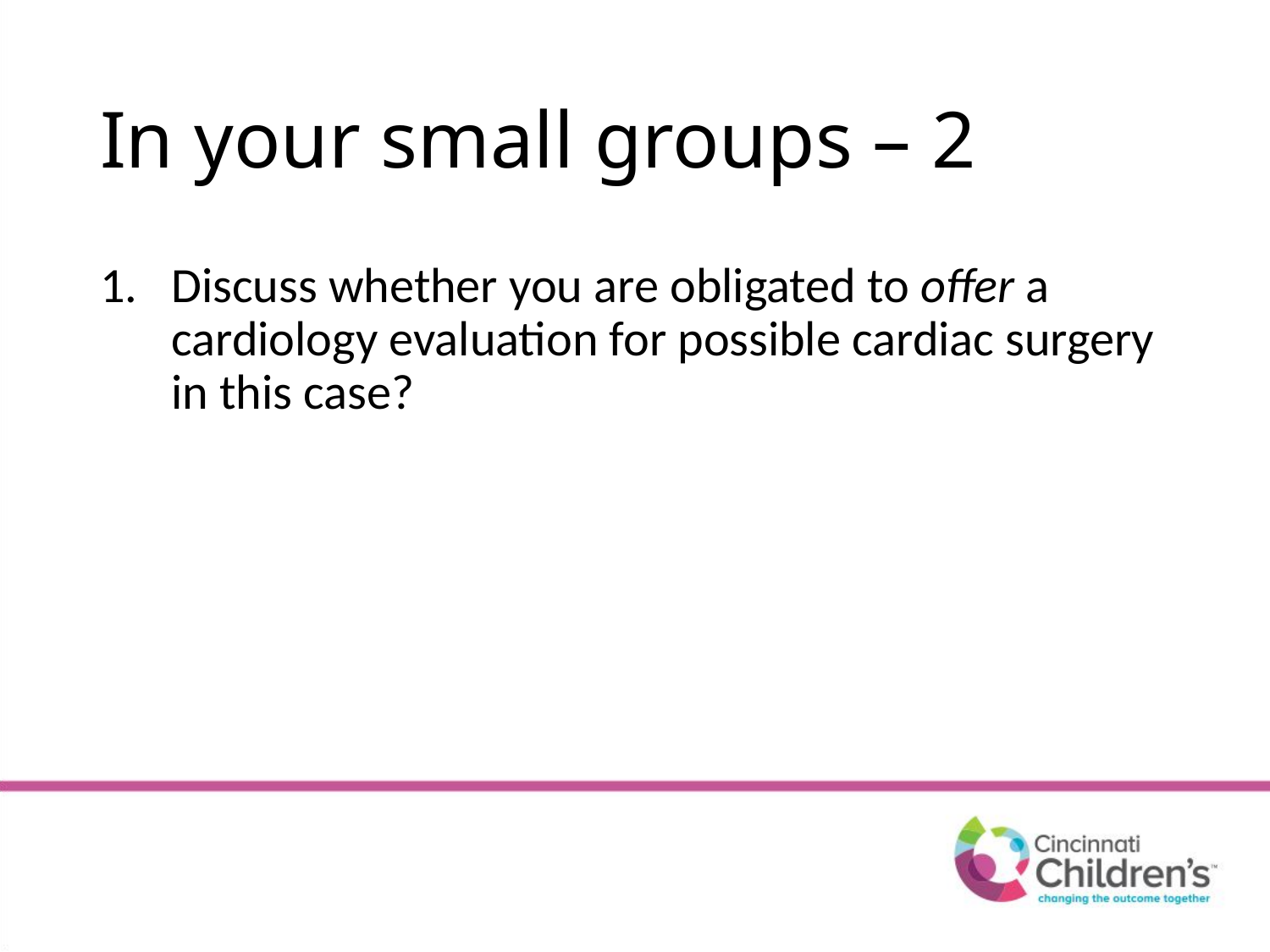

# In your small groups – 2
Discuss whether you are obligated to offer a cardiology evaluation for possible cardiac surgery in this case?

## Slide 15
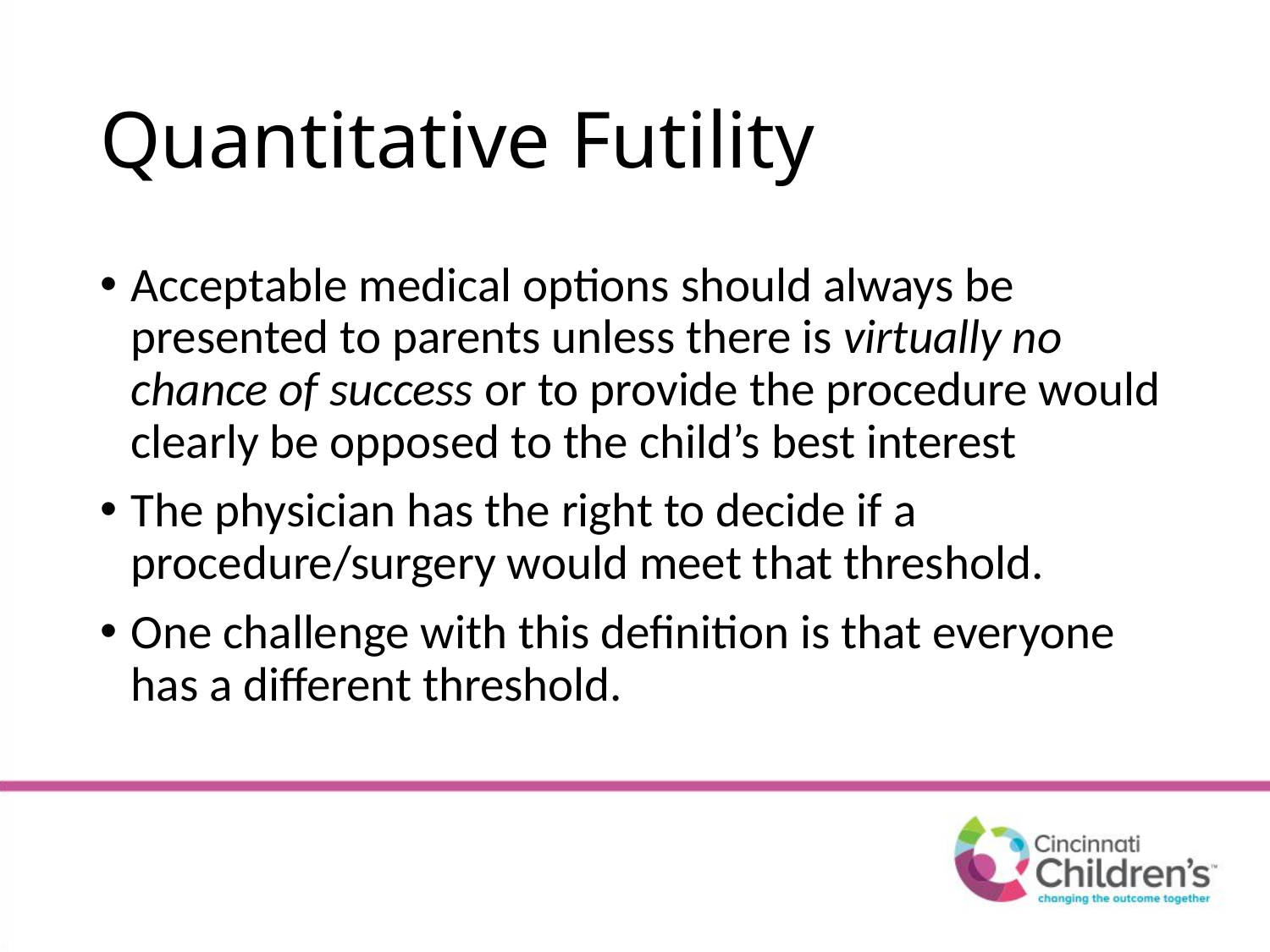

# Quantitative Futility
Acceptable medical options should always be presented to parents unless there is virtually no chance of success or to provide the procedure would clearly be opposed to the child’s best interest
The physician has the right to decide if a procedure/surgery would meet that threshold.
One challenge with this definition is that everyone has a different threshold.

## Slide 16
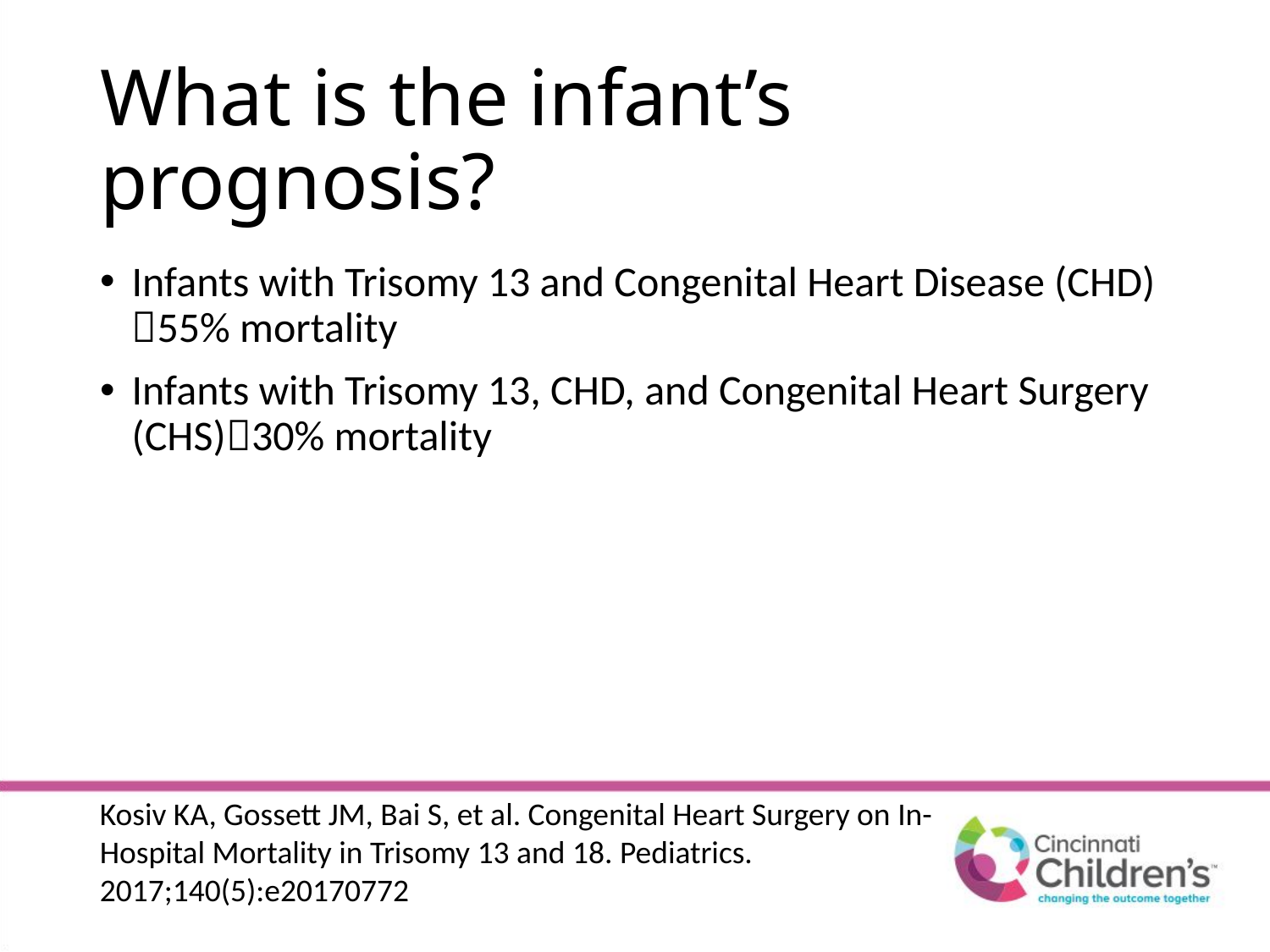

# What is the infant’s prognosis?
Infants with Trisomy 13 and Congenital Heart Disease (CHD) 55% mortality
Infants with Trisomy 13, CHD, and Congenital Heart Surgery (CHS)30% mortality
Kosiv KA, Gossett JM, Bai S, et al. Congenital Heart Surgery on In-Hospital Mortality in Trisomy 13 and 18. Pediatrics. 2017;140(5):e20170772

## Slide 17
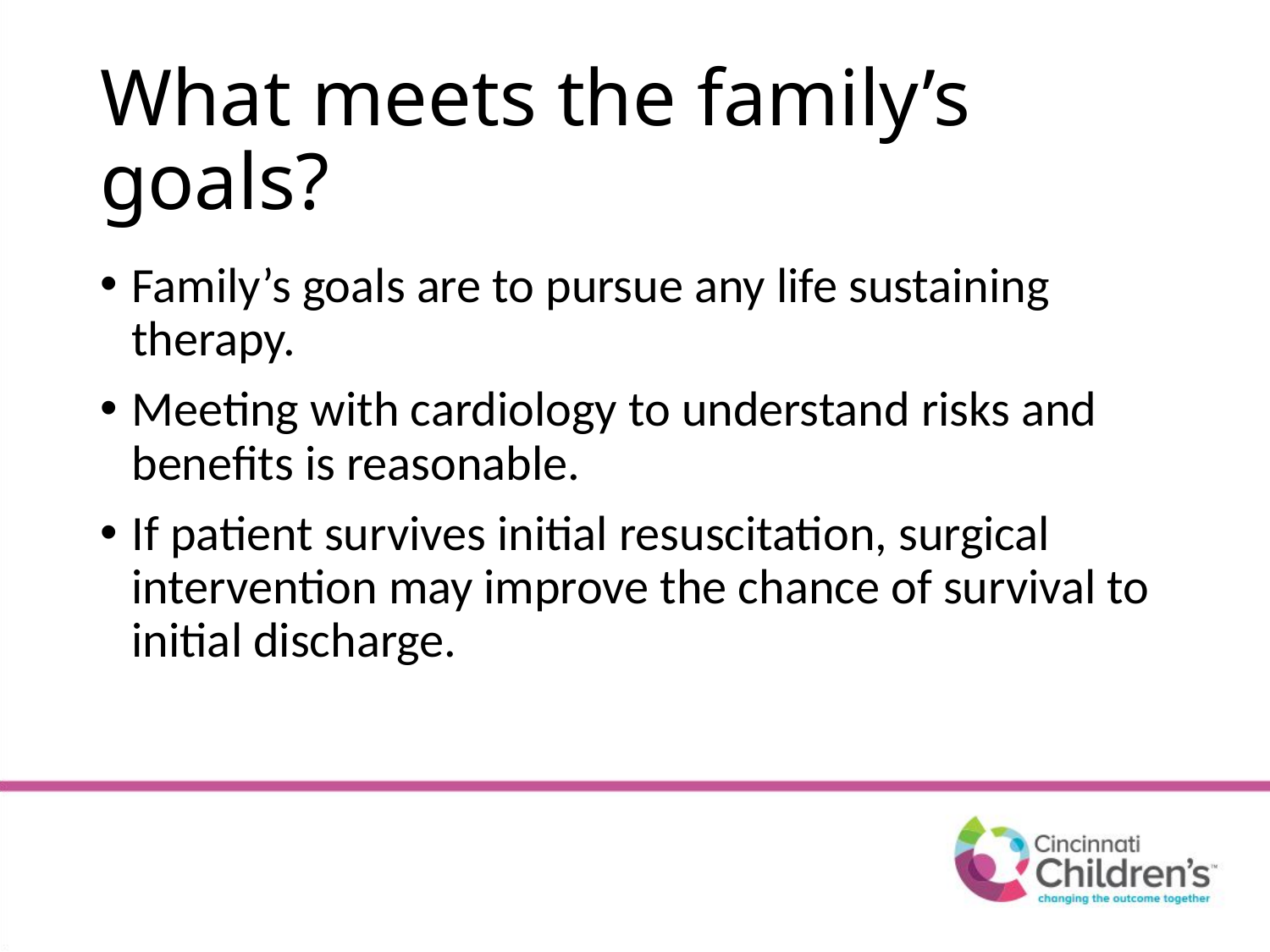

# What meets the family’s goals?
Family’s goals are to pursue any life sustaining therapy.
Meeting with cardiology to understand risks and benefits is reasonable.
If patient survives initial resuscitation, surgical intervention may improve the chance of survival to initial discharge.

## Slide 18
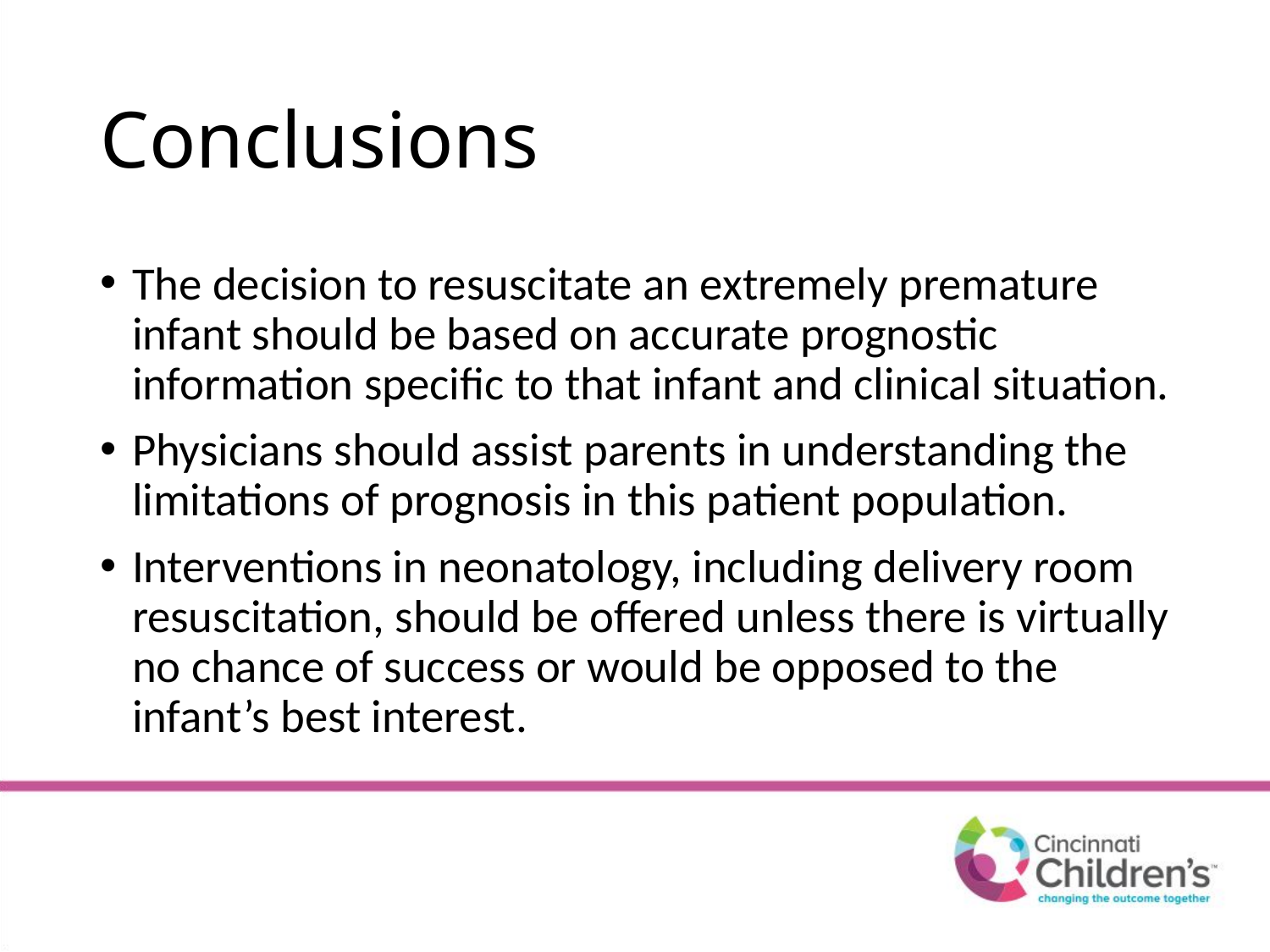

# Conclusions
The decision to resuscitate an extremely premature infant should be based on accurate prognostic information specific to that infant and clinical situation.
Physicians should assist parents in understanding the limitations of prognosis in this patient population.
Interventions in neonatology, including delivery room resuscitation, should be offered unless there is virtually no chance of success or would be opposed to the infant’s best interest.

## Slide 19
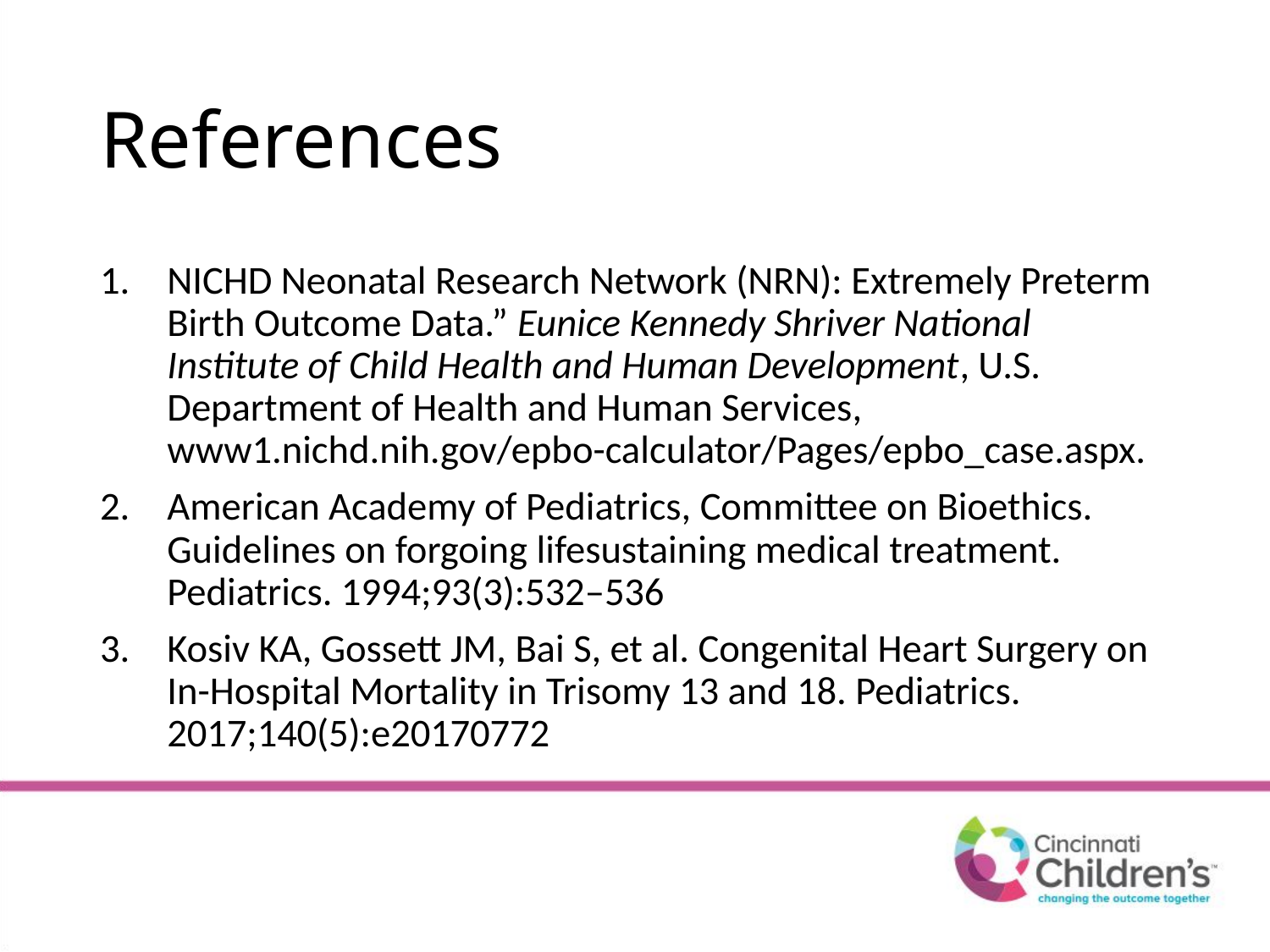

# References
NICHD Neonatal Research Network (NRN): Extremely Preterm Birth Outcome Data.” Eunice Kennedy Shriver National Institute of Child Health and Human Development, U.S. Department of Health and Human Services, www1.nichd.nih.gov/epbo-calculator/Pages/epbo_case.aspx.
American Academy of Pediatrics, Committee on Bioethics. Guidelines on forgoing lifesustaining medical treatment. Pediatrics. 1994;93(3):532–536
Kosiv KA, Gossett JM, Bai S, et al. Congenital Heart Surgery on In-Hospital Mortality in Trisomy 13 and 18. Pediatrics. 2017;140(5):e20170772
