# Supplementary material for: Ethical Dilemmas at the Beginning and End of Life: A Needs-Based, Experience-Informed, Small-Group, Case-Based Curriculum for Pediatric Residents
Source: MedEdPORTAL. 2020 Apr 3;16:10895. doi: 10.15766/mep_2374-8265.10895 (PMC7187913; doi:10.15766/mep_2374-8265.10895)
Supplement: Supplementary file 1 — Medically Provided Fluids Nutrition PowerPoint.pptxMedically Provided Fluids Nutrition Instructor Guide.docxMedically Provided Fluids Nutrition Handout.docxMedically Provided Fluids Nutrition Assessment Questions.docxFutility and Goals of Care PowerPoint.pptxFutility and Goals of Care Instructor Guide.docxFutility and Goals of Care Handout.docxFutility and Goals of Care Assessment Questions.docxEthical Issues in Neonatology PowerPoint.pptxEthical Issues in Neonatology Instructor Guide.docxEthical Issues in Neonatology Assessment Questions.docx [file mep-16-10895-s001.zip › J. Ethical Issues in Neonatology Instructor Guide.docx]

**Ethical Issues in Neonatology
Instructor Directions**

*Set-up:*

- Table Set-up: The group leader should arrange the classroom in multiple individual tables with seats for 6-10 learners.
- Learner Mix: If this is a mixed group of learners (i.e. medical students, interns, upper level residents), there should be a mix of all learners at each table. If possible, an attending physician who has encountered this or a similar challenge, should also be present at the table to provide additional guidance.
- Assessment Tools: Pre and Post Session evaluations should be printed double sided and placed on individual tables. Learners should be instructed to complete the pre-session evaluation when they arrive. They should be asked to complete the post-session evaluation after the session is completed.

*Instructor Overview:*

- Before Session Begins: Prior to the start of the session, the session leader should ask the learners to complete the pre-session assessment.
- Presentation: The session leader will read though the PowerPoint presentation specifically providing details about the case.
- Breakout Sessions: Each time the leader reaches a slide titled “In Your Small Groups” the leader will prompt the teams to discuss the questions on the slide and utilize a hand out if one is available for that section. During each breakout session allow 5-15 minutes for discussion as a small group and 3-5 minutes for groups to share their thoughts with the larger group. Ensure every group gets to report out at least once, but not necessarily for each breakout session. Time for each session should be geared towards the amount of time available to complete the module. For a 45 minute session, 10 minutes should be spent on each breakout session and 5 minutes should be spent to report out discussion highlights. Following residents/learners reporting out when they think/feel about each discussion questions, the leader should continue with the slides to provide didactic and context for the questions addressed.
- Conclusion of Session: As the learners to complete the post-session assessment at the end of the session.

*Additional Session-Specific Instructions and Content Information:*

**“In Your Small Groups – 1”:** The session leader should prompt the residents to break into small groups to discuss.

**Question 1:** Slide 5 describes treatment standards which explains situations when you should over therapy or should not. One consideration when describing whether to offer resuscitation is how effective will it be. This is sometimes framed as quantitative futility (Slide 6). The challenge is everyone has a different threshold of how likely success will be to offer a procedure.

**Question 2:** Accurate prognostic information is important to help patient/family make a decision. Slides 7-11 describe information that should be presented to parents to help determine if how they would weigh resuscitation vs comfort care. In general, resuscitation should be offered unless there is virtually no chance of survival. 22-24 weeks is frequently considered the “grey zone” where resuscitation and comfort care should both be offered. Of note, if providers feel it is appropriate to offer a treatment, they should support the family in their choice regardless if it is what they may personally choose for themselves.

**“In Your Small Groups – 2”:** The session leader should prompt the residents to break into small groups to discuss.

**Question 1:** Slides 19 and 20 provide didactic. When considering whether or not to offer surgery it is important to understand how that surgery would or would not help the baby. The concept of quantitative futility as well as understanding the prognosis with or without surgery can be utilized to decide whether or not this treatment should be offered.

*References:*

The session leader can utilize the references listed to obtain additional content expertise if needed prior to leading the session.
