# Supplementary material for: Ethical Dilemmas at the Beginning and End of Life: A Needs-Based, Experience-Informed, Small-Group, Case-Based Curriculum for Pediatric Residents
Source: MedEdPORTAL. 2020 Apr 3;16:10895. doi: 10.15766/mep_2374-8265.10895 (PMC7187913; doi:10.15766/mep_2374-8265.10895)
Supplement: Supplementary file 1 — Medically Provided Fluids Nutrition PowerPoint.pptxMedically Provided Fluids Nutrition Instructor Guide.docxMedically Provided Fluids Nutrition Handout.docxMedically Provided Fluids Nutrition Assessment Questions.docxFutility and Goals of Care PowerPoint.pptxFutility and Goals of Care Instructor Guide.docxFutility and Goals of Care Handout.docxFutility and Goals of Care Assessment Questions.docxEthical Issues in Neonatology PowerPoint.pptxEthical Issues in Neonatology Instructor Guide.docxEthical Issues in Neonatology Assessment Questions.docx [file mep-16-10895-s001.zip › K. Ethical Issues in Neonatology Assessment Questions.docx]

Palliative Care and Medical Ethics Curriculum Evaluation
Ethical Issues in Neonatology

**Level of Training:**

M3 AI PGY1 PGY2 PGY3 PGY4 PGY5 Other _________________________

**Pre-Conference Evaluation:**

Which of the following are ethical reasons to not offer medical interventions, such as resuscitation of periviable infants?

___ Contrary to the best interest of the patient ___ Physician does not recommend the intervention

___ Physician doesn’t think risk is worth potential benefit ___ Virtually no chance of success

___ Parents do not ask about medical intervention ___ Hospital guideline says to not offer intervention

The likelihood that delivery room resuscitation will be successful is an important factor in deciding whether to offer resuscitation.

True False

A physician’s assessment of the parent’s wishes should influence whether they even offer delivery room resuscitation as a medically acceptable option.

True False

I understand when it is appropriate to not offer or withhold certain medical interventions.

1 = strongly disagree 2 = disagree 3 = neutral 4 = agree 5 = strongly agree

I understand what information goes into a prognostic assessment in the periviable period.

1 = strongly disagree 2 = disagree 3 = neutral 4 = agree 5 = strongly agree

**Post-Conference Evaluation:**

Which of the following are important to predicting mortality outcomes in infants in the periviable period?

___ Gestational Age ___ Maternal Age

___ Hospital’s experience and outcomes ___ Birth weight

___ Latest Fetal Stress Test ___ Antenatal Corticosteroids

___ Infant gender ___ Multiparity vs Single Gestation

Infant outcomes in the periviable period vary by country and hospital.

True False

The NICHD outcome data is fairly accurate and leaves little uncertainty regarding the prognosis for an infant born in the periviable period.

True False

I understand when it is appropriate to not offer or withhold certain medical interventions.

1 = strongly disagree 2 = disagree 3 = neutral 4 = agree 5 = strongly agree

I understand what information goes into a prognostic assessment in the periviable period.

1 = strongly disagree 2 = disagree 3 = neutral 4 = agree 5 = strongly agree

**Prior to the session, I understood** when it is appropriate to not offer or withhold certain medical interventions.

1 = strongly disagree 2 = disagree 3 = neutral 4 = agree 5 = strongly agree

**Prior to the session, I understood** what information goes into a prognostic assessment in the periviable period.

1 = strongly disagree 2 = disagree 3 = neutral 4 = agree 5 = strongly agree
